# Fatty acid β‐oxidation and mitochondrial fusion are involved in cardiac microvascular endothelial cell protection induced by glucagon receptor antagonism in diabetic mice

**DOI:** 10.1111/1753-0407.13458

**Published:** 2023-08-19

**Authors:** Peng Wang, Rui Wei, Xiaona Cui, Zongzhe Jiang, Jin Yang, Lingyun Zu, Tianpei Hong

**Affiliations:** ^1^ Department of Endocrinology and Metabolism, Department of Cardiology and Institute of Vascular Medicine Peking University Third Hospital Beijing China; ^2^ NHC Key Laboratory of Cardiovascular Molecular Biology and Regulatory Peptides; Key Laboratory of Molecular Cardiovascular Science, Ministry of Education Beijing Key Laboratory of Cardiovascular Receptors Research Beijing China; ^3^ Department of Endocrinology and Metabolism The Affiliated Hospital of Southwest Medical University Luzhou China

**Keywords:** cardiac microvascular endothelial cells, fatty acid β‐oxidation, glucagon receptor, type 2 diabetes, 心脏微血管内皮细胞, 脂肪酸β‐氧化, 胰高血糖素受体, 2型糖尿病

## Abstract

**Introduction:**

The role of cardiac microvascular endothelial cells (CMECs) in diabetic cardiomyopathy is not fully understood. We aimed to investigate whether a glucagon receptor (GCGR) monoclonal antibody (mAb) ameliorated diabetic cardiomyopathy and clarify whether and how CMECs participated in the process.

**Research Design and Methods:**

The *db*/*db* mice were treated with GCGR mAb or immunoglobulin G (as control) for 4 weeks. Echocardiography was performed to evaluate cardiac function. Immunofluorescent staining was used to determine microvascular density. The proteomic signature in isolated primary CMECs was analyzed by using tandem mass tag‐based quantitative proteomic analysis. Some target proteins were verified by using western blot.

**Results:**

Compared with *db*/*m* mice, cardiac microvascular density and left ventricular diastolic function were significantly reduced in *db*/*db* mice, and this reduction was attenuated by GCGR mAb treatment. A total of 199 differentially expressed proteins were upregulated in *db*/*db* mice versus *db*/*m* mice and downregulated in GCGR mAb‐treated *db*/*db* mice versus *db*/*db* mice. The enrichment analysis demonstrated that fatty acid β‐oxidation and mitochondrial fusion were the key pathways. The changes of the related proteins carnitine palmitoyltransferase 1B, optic atrophy type 1, and mitofusin‐1 were further verified by using western blot. The levels of these three proteins were upregulated in *db*/*db* mice, whereas this upregulation was attenuated by GCGR mAb treatment.

**Conclusion:**

GCGR antagonism has a protective effect on CMECs and cardiac diastolic function in diabetic mice, and this beneficial effect may be mediated via inhibiting fatty acid β‐oxidation and mitochondrial fusion in CMECs.

## Graphical abstract

Highlights

- Glucagon receptor (GCGR) monoclonal antibody (mAb) improves cardiac function and attenuates microvascular damage in *db/db* mice.
- GCGR mAb ameliorates the oxidative stress of cardiac microvascular endothelial cells in diabetic mice.
- GCGR mAb counteracts the diabetes‐induced upregulation of carnitine palmitoyltransferase 1B, optic atrophy type 1, and mitofusin‐1.
- Fatty acid β‐oxidation and mitochondrial fusion are involved in GCGR mAb's effect.

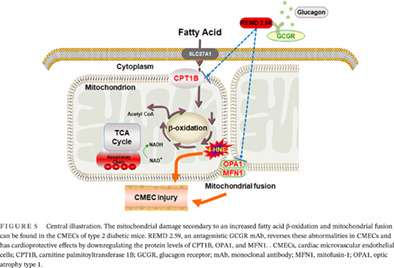

## 摘要

介绍:心脏微血管内皮细胞(CMECs)在糖尿病心肌病中的作用尚未完全清楚。我们的目的是研究胰高血糖素受体(GCGR)单克隆抗体(mAb)是否改善了糖尿病心肌病，并阐明CMECs是否以及如何参与这一过程

方法: GCGR mAb或免疫球蛋白G(作为对照)治疗db/db小鼠4周。超声心动图检查以评估心脏功能。免疫荧光染色测定微血管密度。串联质谱标签(TMT)定量蛋白质组学分析分离的原代CMECs中蛋白质组学特征。使用蛋白质印迹验证一些靶蛋白

结果:与db/m小鼠相比，db/db小鼠的心脏微血管密度和左心室舒张功能显著降低，GCGR mAb治疗可减轻这种降低。与db/m小鼠相比，db/db小鼠中共有199个差异表达蛋白(DEP)上调，与db/db小鼠相比，GCGR mAb治疗的db/db小鼠中共有199个差异表达蛋白下调。富集分析表明，脂肪酸β‐氧化和线粒体融合是关键途径。蛋白质印迹实验进一步确认存在相关蛋白CPT1B、OPA1和MFN1的变化。db/db小鼠中这三种蛋白质水平上调，而GCGR mAb治疗可减轻这种上调

结论:GCGR拮抗剂对糖尿病小鼠CMECs和心脏舒张功能具有保护作用，这种有益作用可能是通过抑制CMECs中脂肪酸β‐氧化和线粒体融合来介导的

## 1 INTRODUCTION

Diabetes has high prevalence worldwide. The weighted prevalence of diabetes and prediabetes diagnosed in the most recent national cross‐sectional study were 12.8% and 35.2%, respectively, among adults living in China.^1^ Diabetic complications are an urgent health problem currently. Cardiovascular complications, including diabetic cardiomyopathy, account for more than 80% of diabetic deaths.^2^ Myocardial stiffness and diastolic dysfunction in the early stage of diabetic cardiomyopathy are closely related to abnormality in the function of cardiac microvascular endothelial cells (CMECs).^3^ The decrease and dysfunction of CMECs in diabetic individuals may affect the material supply and energy metabolism of myocardial cells and ultimately cause structural heart abnormalities and cardiac dysfunction.^4^ However, the precise mechanism is not fully understood, and the effective therapy for diabetic cardiomyopathy is highly needed.

Traditionally, diabetes therapy mainly revolves around insulin and pancreatic β cells. Nowadays, the role of pancreatic α cells and the therapeutic potential of targeting glucagon and glucagon receptor (GCGR) have been gradually recognized.^5^ As a major catabolic hormone, glucagon stimulates the decomposition of liver glycogen into glucose and promotes gluconeogenesis and inhibits glycogen synthesis and glycolysis.^6^ GCGR is a member of the G protein‐coupled receptor family, which mainly functions by activating stimulatory G proteins.^7^ Elevation in circulating glucagon level can be observed in patients with type 1 and type 2 diabetes, and excessive activation of GCGR promotes the progression of diabetes.^5^, ^6^ Inhibition of glucagon secretion (eg, by glucagon‐like peptipe‐1‐based agents), or antagonism of the glucagon binding with GCGR (eg, by glucagon neutralizing antibody, GCGR antibody, and small molecular GCGR antagonists) is able to improve blood glucose control.^8^, ^9^ The current ongoing clinical trials of GCGR antagonists were summarized in a review.^10^ The representative GCGR antagonists were the GCGR antibodies RN909 and REMD‐477 (volagidemab). RN909 has completed the phase I clinical trials.^11^ REMD‐477 has now completed phase I and phase II clinical trials.^12^ REMD 2.59, a fully competitive antagonistic human GCGR monoclonal antibody (mAb) for GCGR,^13^ has only one amino acid difference with REMD‐477. This antibody shows a higher affinity to GCGR than the cognate ligand,^13^ which enables it to maintain long‐lasting efficacy even in hyperglucagonemia. Our previous study proved that GCGR mAb significantly lowered the fasting and random blood glucose and improved glucose tolerance in type 2 diabetic mice.^8^, ^14^, ^15^ Notably, GCGR mAb also has the other benefits. For instance, GCGR mAb treatment reduced the size of myocardial infarction, improved myocardial remodeling after coarctation of the aortic arch, and had a protective effect on cardiac function.^16^, ^17^, ^18^ Moreover, we found that GCGR mAb promoted intestinal L cell proliferation, increased pancreatic α cell, δ cell, and β cell mass in diabetic mice.^8^, ^9^, ^14^, ^15^, ^19^ However, the underling mechanisms of the organ protection (especially heart protection) induced by GCGR mAb are still blurred.

To investigate the cardioprotective effect of GCGR mAb and its possible mechanism, CMECs were isolated from *db*/*m* mice, *db*/*db* mice and GCGR mAb‐treated *db*/*db* mice, and tandem mass tag (TMT)‐based quantitative proteomic analysis was used to identify the proteomic signature of CMECs associated with the development and amelioration of diabetic cardiomyopathy. Furthermore, we chose some candidate proteins for further verification to clarify the involvement of CMECs in the development and amelioration of diabetic cardiomyopathy.

## 2 RESEARCH DESIGN AND METHODS

### 2.1 Animal models and intervention

All animal experiments were performed at Peking University Health Science Center, and all protocols were approved by the Institutional Animal Care and Use Committee (the approval number: LA2018316). After 1‐week acclimatization, 8‐week‐old male *db/db* mice (GemPharmatech Co. Ltd., Nanjing, China), a type 2 diabetic model, were randomized into two groups (13 mice per group). The diabetic mice were treated for 4 weeks by weekly intraperitoneal administration of GCGR mAb REMD 2.59 (5 mg/kg; REMD Biotherapeutics, Camarillo, CA, USA) or immunoglobulin G (IgG, as control). The age‐matched male *db*/*m* mice treated with IgG served as normal controls. All mice were maintained at 22°C under a 12‐h day/night cycle with free access to food and water. After the intervention, 13 mice per group were evaluated by cardiac ultrasound. Subsequently, nine mice per group were used to isolate primary CMECs, and four mice per group were subjected to histological analysis.

### 2.2 Blood glucose and insulin measurement

The body weight and blood glucose were measured every week. Blood samples for glucose detection were collected from the tail vein and measured by the glucose oxidase method using a hand‐held OneTouch Ultra glucometer (LifeScan, Milpitas, CA, USA). If the glucose level was greater than 33.3 mmol/L (the upper detection limit of the glucometer), the value was recorded as 33.3 mmol/L.

Blood samples for insulin detection were collected from the orbital vein. Aprotinin (1 μg/mL; Sigma–Aldrich, St. Louis, MO, USA) and heparin sodium (1000 IU/mL; Qianhong Bio‐pharma, Changzhou, China) were added to each blood sample. Insulin levels were evaluated with specific enzyme‐linked immunosorbent assay kits (Merck Millipore, Billerica, MA, USA) following the manufacturer's instructions.

### 2.3 Echocardiography evaluation

Echocardiography was performed to evaluate the cardiac geometry, systolic and diastolic function as previously described.^20^ The detailed methods are in Data S1.

### 2.4 Histological analysis

The mice were perfused with cold phosphate buffer solution. Hearts were harvested, fixed with 4% paraformaldehyde for 24 h, and embedded in paraffin. Serial sections were obtained at 6 μm intervals for the paraffin‐embedded tissue. The detailed methods are in Data S1.

### 2.5 Isolation of primary CMECs from mouse heart tissues

Primary mouse CMECs were isolated as previously described^21^ with slightly adjusted. The detailed methods are in Data S1.

### 2.6 Proteomic analysis

To obtain enough cell number and eliminate individual difference, primary CMECs used for proteomic analysis were isolated from the heart tissues of every three mice belonging to the same treatment group. There were three independent cell preparations in each group. The detailed steps of proteomic analysis are in supplementary information. The mass spectrometry proteomic data have been deposited to the ProteomeXchange Consortium (http://proteomecentral.proteomexchange.org) via the iProX partner repository^22^ with the data set identifier PXD027916.

### 2.7 Western blot

The denatured protein (approximately 30 μg) was separated by 10% sodium dodecyl sulfate‐polyacrylamide gel electrophoresis and transferred to a nitrocellulose membrane. The membranes were blocked with 5% bovine serum albumin, incubated with primary antibodies at 4°C overnight and IRDye 800CW‐conjugated goat anti‐rabbit or anti‐mouse secondary antibodies (1:10000; Rockland, Waltham, MA, USA) for 1 h, and visualized with an Odyssey 290 infrared imaging system (LI‐COR Biosciences, Lincoln, NE, USA). The primary antibodies and their dilutions are as follows: rabbit anti‐carnitine palmitoyltransferase 1B (CPT1B, 1:1000; Proteintech, Rosemont, IL, USA), rabbit anti‐dynamin‐like 120 kDa protein (optic atrophy type 1 [OPA1], 1:1000; Abcam, Cambridge, UK), rabbit anti‐mitofusin‐1 (MFN1, 1:1000; Abcam), rabbit anti‐4‐hydroxynonenal (4‐HNE, 1:1000; Abcam), and mouse anti‐β‐actin (1:1000; Abcam). β‐actin was used as a loading control. The gray value of each band was analyzed using Image J software (National Institutes of Health, Bethesda, MD, USA).

### 2.8 Statistical analysis

Data are expressed as mean ± SD. Statistical differences were assessed by one‐way or two‐way analysis of variance (ANOVA) followed by the post‐hoc Tukey–Kramer test. Statistical analyses were carried out using Prism 7.0 (GraphPad Software Inc., La Jolla, CA, USA). *p* < .05 was considered statistically significant.

## 3 RESULTS

### 3.1 GCGR mAb ameliorates hyperglycemia, improves cardiac function, and attenuates cardiac microvascular damage in *db*/*db* mice

**FIGURE 1:**
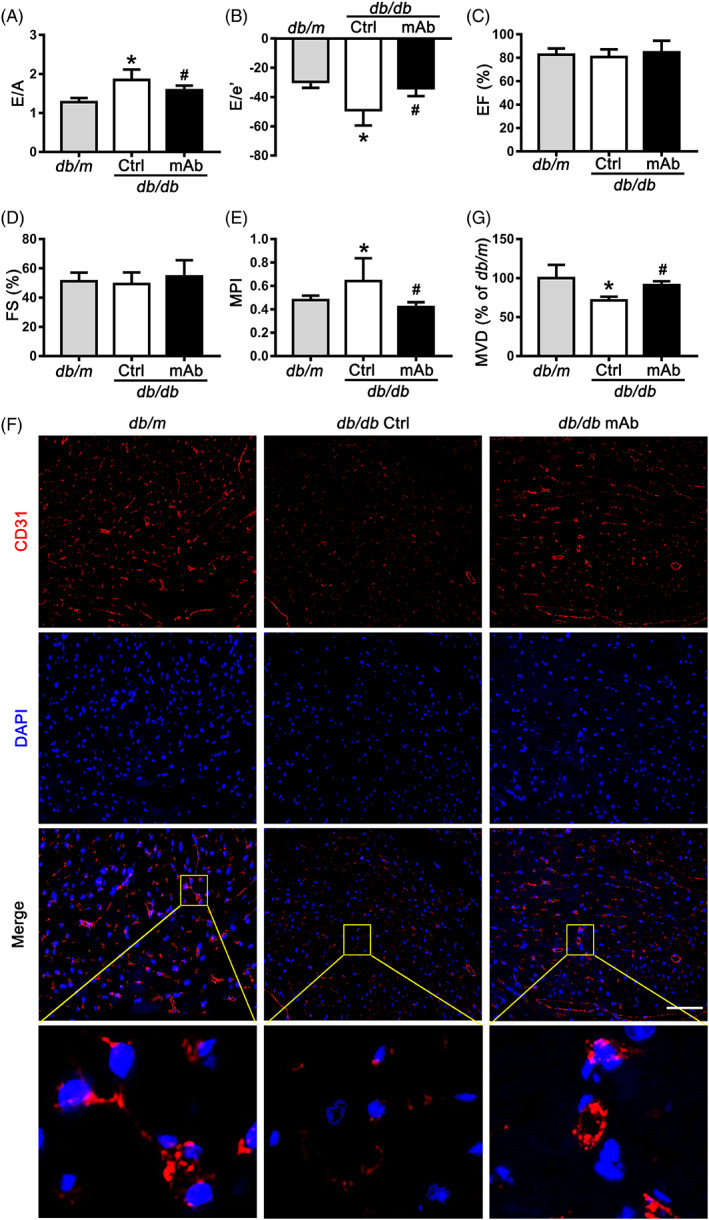
Echocardiography and histological analysis in *db*/*db* mice treated with GCGR mAb or IgG (as control) for 4 weeks. Age‐matched *db*/*m* mice served as the normal controls. (A) E/A; (B) E/e′; (C) EF; (D) FS; and (E) MPI as determined by echocardiography. (F) Immunofluorescent staining of CD31 to determine the cardiac microvascular density in the heart tissues. Scale bar = 50 μm. (G) Quantification of the microvascular density as measured by the CD31‐positive cells in (F). *n* = 13 mice per group in (A–E) and *n* = 4 mice per group in (F and G ). Data are shown as mean ± S.D. Statistical analysis was conducted by one‐way analysis of variance followed by the post hoc Tukey–Kramer test. *p < .05 vs. *db*/*m*; ^#^ *p* < .05 vs. *db*/*db* Ctrl. A, the peak flow velocities during late diastole; Ctrl, control; DAPI, 4′, 6‐diamidino‐2‐phenylindole; E, the peak flow velocities during early diastole; e′, early‐diastolic peak velocity; EF, ejection fraction; FS, fractional shortening; GCGR, glucagon receptor; IgG, immunoglobulin G; mAb, monoclonal antibody; MPI, myocardial performance index; MVD, microvascular density.

During the 4‐week treatment, there was no significant difference between GCGR mAb and IgG control treatment groups in terms of body weight in *db*/*db* mice (Figure S1A). Treatment with GCGR mAb for 4 weeks in *db*/*db* mice significantly lowered the random blood glucose to the level of the normal control *db*/*m* mice (Figure S1B). Plasma insulin level in GCGR mAb‐treated *db*/*db* mice was modestly but significantly higher than that in the control group (Figure S1C). Echocardiography was performed after the 4‐week treatment. A significant increase in E/A ratio and a remarkable decrease in E/e′ ratio were observed in *db*/*db* mice as compared to *db*/*m* mice, which indicated left ventricular diastolic dysfunction in *db*/*db* mice. The increased E/A ratio and decreased E/e′ ratio in the diabetic mice were significantly attenuated by GCGR mAb treatment (Figure 1A,B; Table S1). No significant difference in cardiac systolic function, as indicated by left ventricular ejection fraction and fractional shortening, was observed among the three groups (Figure 1C,D; Table S1). Myocardial performance index, a Doppler index reflecting a combined diastolic and systolic myocardial performance, was significantly increased in *db*/*db* mice compared with *db*/*m* mice, and this increment was eliminated by GCGR mAb treatment (Figure 1E; Table S1).

In histological analysis, the myocardial structure among the three groups showed no difference (Figure S2A–D). To investigate the possible reason for the protection of cardiac diastolic function induced by GCGR mAb, we also detected the cell apoptosis and inflammation in heart tissues. Results showed that there was no significant difference among the three groups in terms of the percentage of TUNEL staining‐positive apoptotic cells (Figure S2E,F) and intercellular adhesion molecule 1 or vascular cell adhesion molecule 1 ‐positive cells (Figure S3), further supporting the concept that no significant cardiac structural abnormalities can be found in the early stage of diabetic cardiomyopathy. Because diastolic dysfunction is closely associated with abnormality in the CMEC function,^3^ we detected cardiac microvascular density using immunofluorescent staining. Results showed that cardiac microvascular density was significantly reduced in *db*/*db* mice, whereas this reduction was attenuated by GCGR mAb treatment (Figure 1F,G). These results implied that the change in CMECs might be involved in the cardiac protection induced by GCGR mAb.

### 3.2 Proteomic signature of CMECs in *db*/*m* mice, *db*/*db* mice, and GCGR mAb‐treated *db*/*db* mice

**FIGURE 2:**
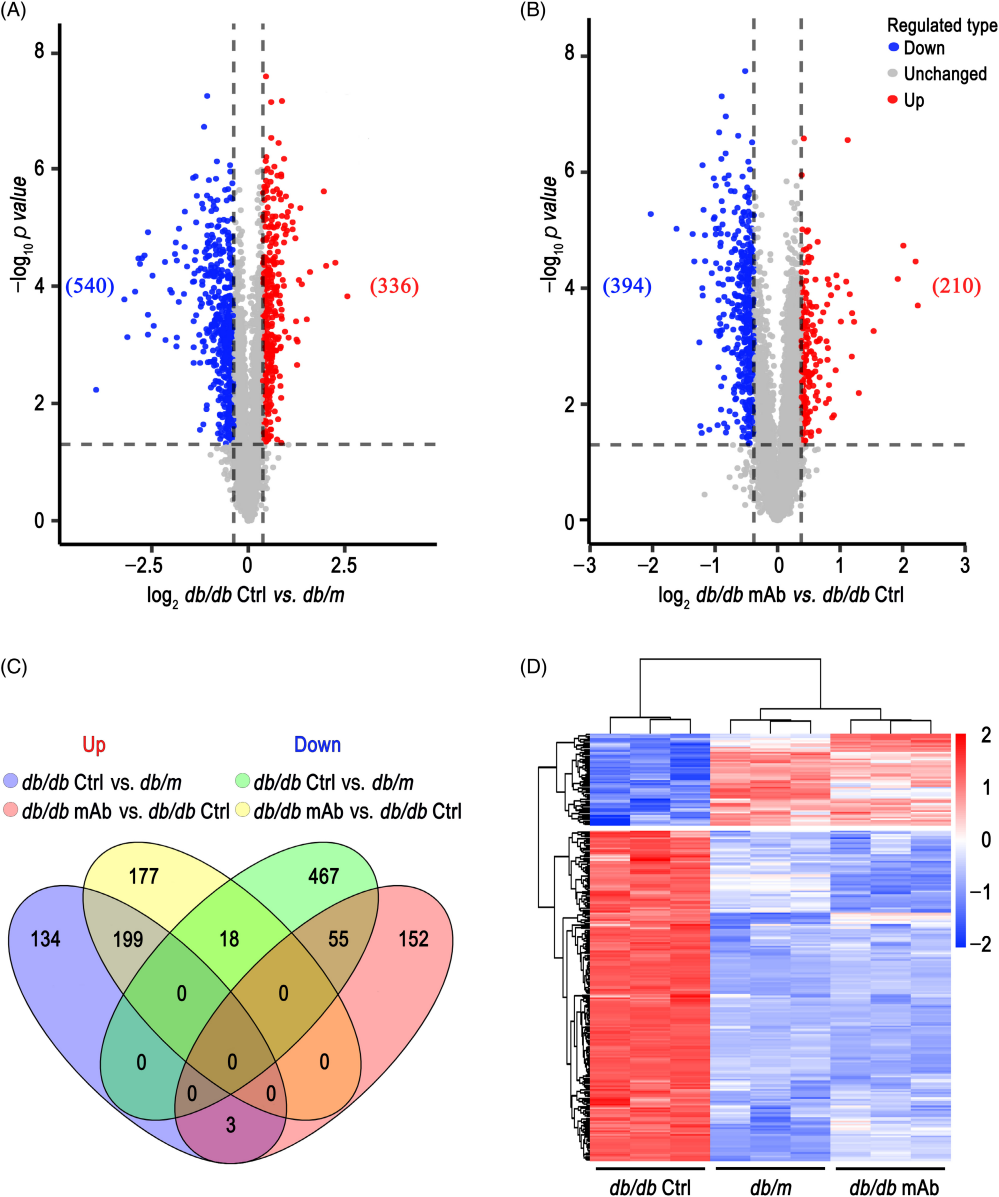
Identification of differentially expressed proteins (DEPs). (A) Volcano plot showing the quantitative protein expression between *db*/*db* mice and *db*/*m* mice. (B) Volcano plot exhibiting the quantitative protein expression between GCGR mAb‐treated *db*/*db* mice and *db*/*db* mice. (C) Venn diagram showing the overlapped DEPs in *db*/*db* mice vs. *db*/*m* mice and in GCGR mAb‐treated *db*/*db* mice vs. *db*/*db* mice. (D) The heatmap of hierarchical clustering analysis on the DEPs. Rows represent proteins and columns represent different samples. The proteins in CMECs isolated from every three mice were used as an independent sample. *n* = 3 samples per group. CMECs, cardiac microvascular endothelial cells; Ctrl, control; GCGR, glucagon receptor; mAb, monoclonal antibody.

To investigate the possible mechanism, we isolated primary mouse CMECs for proteomic analysis. A total of 43 545 peptides and 5685 proteins were identified, of which 4909 proteins were quantifiable. A total of 876 differentially expressed proteins (DEPs) were quantified in *db*/*db* mice compared with *db*/*m* mice, among which 336 DEPs were upregulated and 540 DEPs were downregulated. A total of 604 DEPs were quantified in GCGR mAb‐treated *db*/*db* mice compared with *db*/*db* mice, among which 210 DEPs were upregulated and 394 DEPs were downregulated (Figure 2A,B; Tables S2 and S3). Between 336 upregulated DEPs (*db*/*db* mice vs. *db*/*m* mice) and 394 downregulated DEPs (GCGR mAb‐treated *db*/*db* mice vs. *db*/*db* mice), there were 199 DEPs overlapped. Between 540 downregulated DEPs (*db*/*db* mice vs. *db*/*m* mice) and 210 upregulated DEPs (GCGR mAb‐treated *db*/*db* mice vs. *db*/*db* mice), 55 DEPs overlapped (Figure 2C). The hierarchical clustering for the overlapped 254 (199 + 55) DEPs among the three groups revealed that the protein profile in GCGR mAb‐treated *db*/*db* mice was very similar to that in *db*/*m* mice, but had a significant difference from that in *db/db* mice (Figure 2D).

**FIGURE 3:**
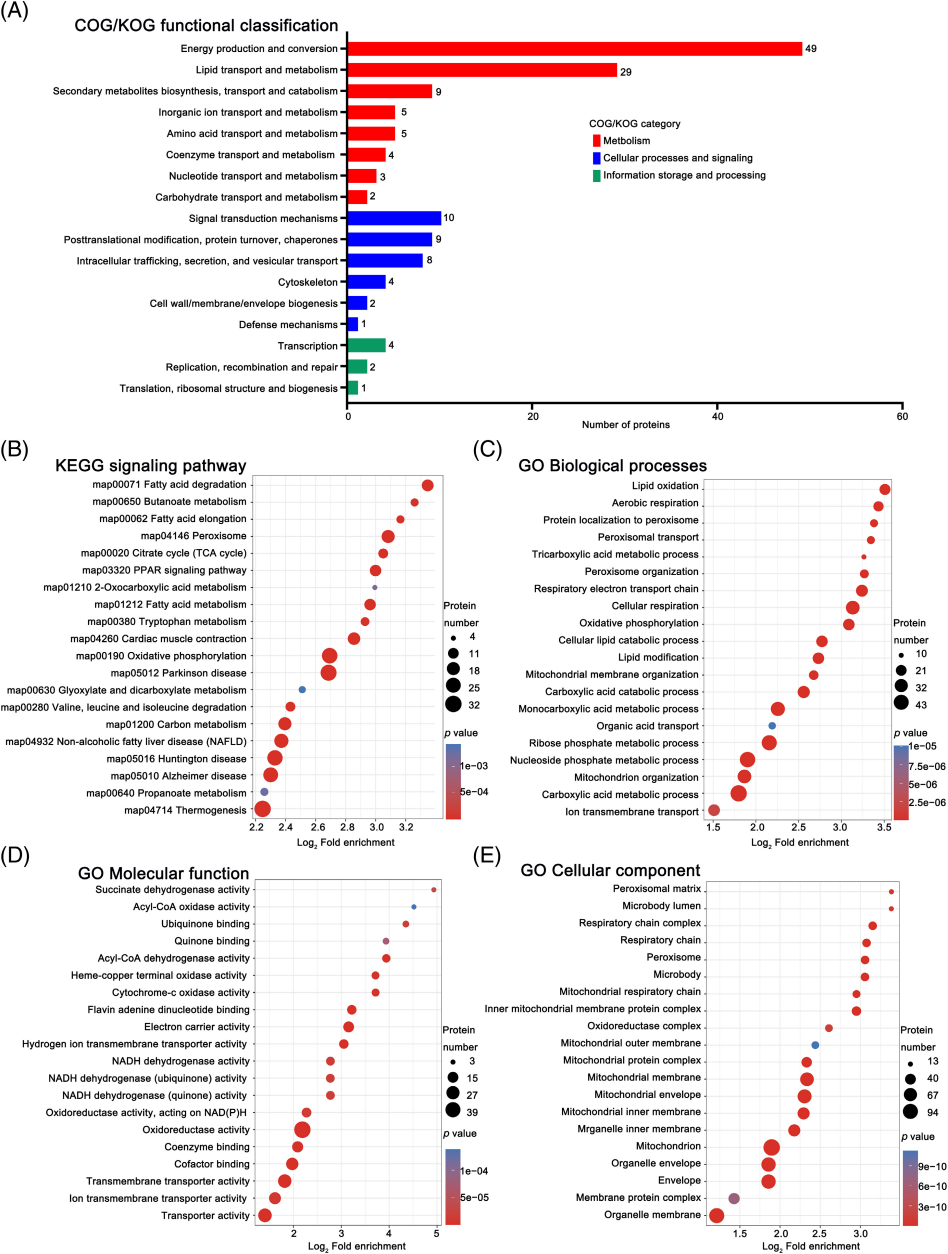
Bioinformatic analysis of differentially expressed proteins (DEPs). All the overlapped 199 DEPs, which were upregulated in *db*/*db* mice vs. *db*/*m* mice and downregulated in GCGR mAb‐treated *db*/*db* mice vs. *db*/*db* mice, underwent the enrichment analyses of COG/KOG functional classification (A), KEGG signaling pathway (B), and GO annotation of biological processes (C), molecular function (D), and cellular component (E). COG/KOG, clusters of orthologous groups of proteins; Ctrl, control; GCGR, glucagon receptor; GO, gene ontology; KEGG, Kyoto Encyclopedia of Genes and Genomes; mAb, monoclonal antibody.

To gain insight into the functions of the overlapped 199 DEPs, we performed clusters of orthologous groups of proteins (COG/KOG) analysis, Kyoto Encyclopedia of Genes and Genomes (KEGG) pathway enrichment analysis, and Gene Ontology (GO) annotation enrichment analysis. COG/KOG analysis showed that the DEPs were mainly involved in energy production and conversion (49 proteins), and lipid transport and metabolism (29 proteins) (Figure 3A). Among the enriched KEGG pathways, the fatty acid degradation was most significantly affected, followed by the several other pathways pertaining to energy metabolism (eg, butanoate metabolism, fatty acid elongation, peroxisome, oxidative phosphorylation, tricarboxylic acid (TCA) cycle, fatty acid metabolism, and tryptophan metabolism) (Figure 3B). These results suggested that reprograming of energy metabolism was likely to serve as a key mediator of the GCGR mAb‐induced cardioprotective effects in *db*/*db* mice. GO annotation analysis also demonstrated the enrichment of biological processes involved in energy metabolism, such as lipid oxidation, aerobic respiration, and protein localization to peroxisome (Figure 3C). Moreover, the GO analysis of molecular function showed that the protein functions related to acyl‐CoA dehydrogenase activity, cytochrome c oxidase activity, and electron carrier activity were highly enriched (Figure 3D). These results indicated that the fatty acid β‐oxidation process in energy metabolism would be important for the GCGR mAb‐induced cardioprotective effects. Moreover, the GO analysis of cellular components emphasized the mitochondria (Figure 3E), where most of cellular energy metabolism processes occur. These results suggested that GCGR mAb had cardioprotective effects by regulating the energy metabolism processes, especially fatty acid β‐oxidation, in the CMECs of type 2 diabetic mice.

### 3.3 Involvement of fatty acid β‐oxidation and mitochondrial fusion in the damage and protection of CMECs in *db/db* mice

**FIGURE 4:**
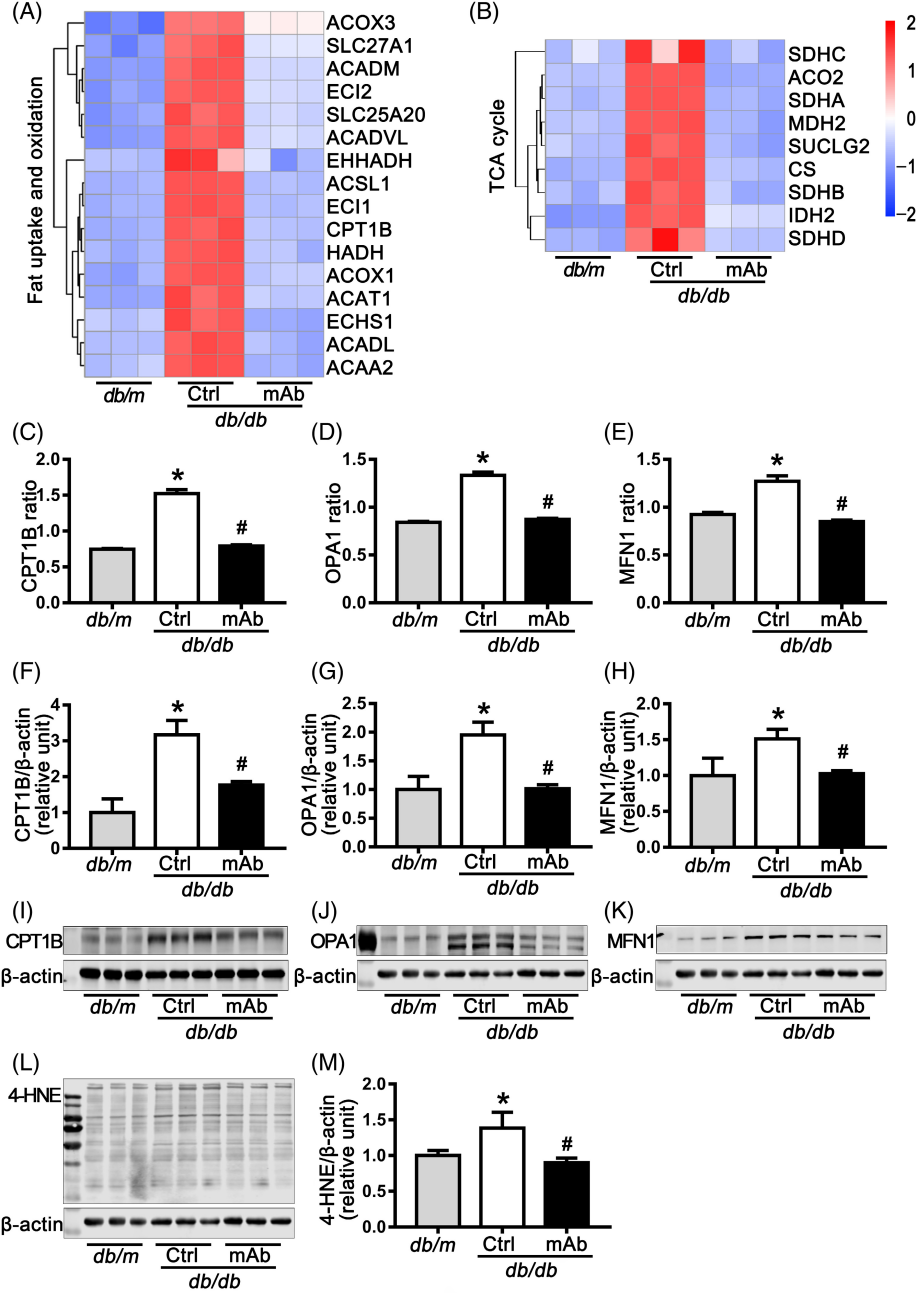
Selection and identification of differentially expressed proteins (DEPs). (A) Heatmaps depicting relative expression levels of DEPs involved in fat uptake and oxidation. (B) Heatmaps showing relative expression levels of DEPs involved in tricarboxylic acid (TCA) cycle. (C–E) Mass spectrometry intensities of CPT1B, OPA1, and MFN1. (F–K) Quantification (F–H) and image (I–K) of western blot analysis for the protein levels of CPT1B, OPA1, and MFN1 in *db*/*m* mice, *db*/*db* mice and GCGR mAb‐treated *db*/*db* mice. (L and M) The level of 4‐hydroxynonenal (4‐HNE) modified protein, an oxidative stress marker, was detected by western blot. The proteins in CMECs isolated from every three mice were used as an independent sample. *n* = 3 samples per group. Data are shown as mean ± SD. Statistical analysis was conducted by one‐way analysis of variance followed by the post hoc Tukey–Kramer test. *p < .05 vs. *db*/*m*; ^#^ *p* < .05 vs. *db*/*db* Ctrl. CMECs, cardiac microvascular endothelial cells; CPT1B, carnitine palmitoyltransferase 1B; Ctrl, control; GCGR, glucagon receptor; mAb, monoclonal antibody; MFN1, mitofusin‐1; OPA1, optic atrophy type 1.

Subsequently, we focused on the fatty acid β‐oxidation process. Heatmaps depicting the relative levels of proteins involved in fat uptake and oxidation were presented (Figure 4A). Results revealed that CMECs in *db*/*db* mice increased fat utilization, as indicated by upregulation of the transporters and proteins involved in mitochondrial fatty acid β‐oxidation (SLC27A1, SLC25A20, ACSL1, CPT1B, ACOX1, ACOX3, ACADM, ACADL, ACADVL, ECHS1, HADH, ACAA2, ACAT1, ECI1, ECI2, and EHHADH), whereas this upregulation was attenuated by GCGR mAb treatment (Figure 4A). There was no obvious change in the proteins involved in fat synthesis (data not shown). In addition, the levels of several TCA cycle‐related proteins were upregulated in *db*/*db* mice, and this effect was attenuated by GCGR mAb treatment (Figure 4B).

We selected CPT1B, which is located in outer mitochondrial membrane and is a rate‐limiting enzyme of mitochondrial fatty acid β‐oxidation by controlling mitochondrial entry of long chain fatty acids,^23^ for further validation. Our proteomic analysis showed that CPT1B level was 2.04‐fold higher in *db*/*db* mice than in *db*/*m* mice, and 1.92‐fold lower in GCGR mAb‐treated *db*/*db* mice than in *db*/*db* mice (both *p* < .001) (Figure 4C). In addition, western blot analysis confirmed the identical change pattern in CPT1B level (Figure 4F,I).

Because most of cellular energy metabolism processes occur in mitochondria, we explored the change of proteins involved in mitochondrial function. We found that the protein levels of OPA1 and MFN1, which participate in mitochondrial fusion, were significantly upregulated in *db*/*db* mice compared with *db*/*m* mice; whereas this upregulation was attenuated by GCGR mAb treatment in *db*/*db* mice (Figure 4D–K).

Enhancement of the fatty acid β‐oxidation process and imbalance between the mitochondrial fission and fusion in endothelial cells may be associated with increased oxidative stress.^24^, ^25^, ^26^, ^27^ The molecule 4‐hydroxynonenal (4‐HNE) has been implicated as a key mediator of oxidative stress‐induced cell death. It is a stable product of lipid peroxidation and may contribute to the cytotoxic effects of oxidative stress.^28^ In this study, the results showed that the level of 4‐HNE modified protein, an oxidative damage product, was higher in *db/db* mice than in *db/m* mice; whereas GCGR mAb treatment significantly reduced the level of 4‐HNE modified protein in *db/db* mice (Figure 4L,M).

**FIGURE 5:**
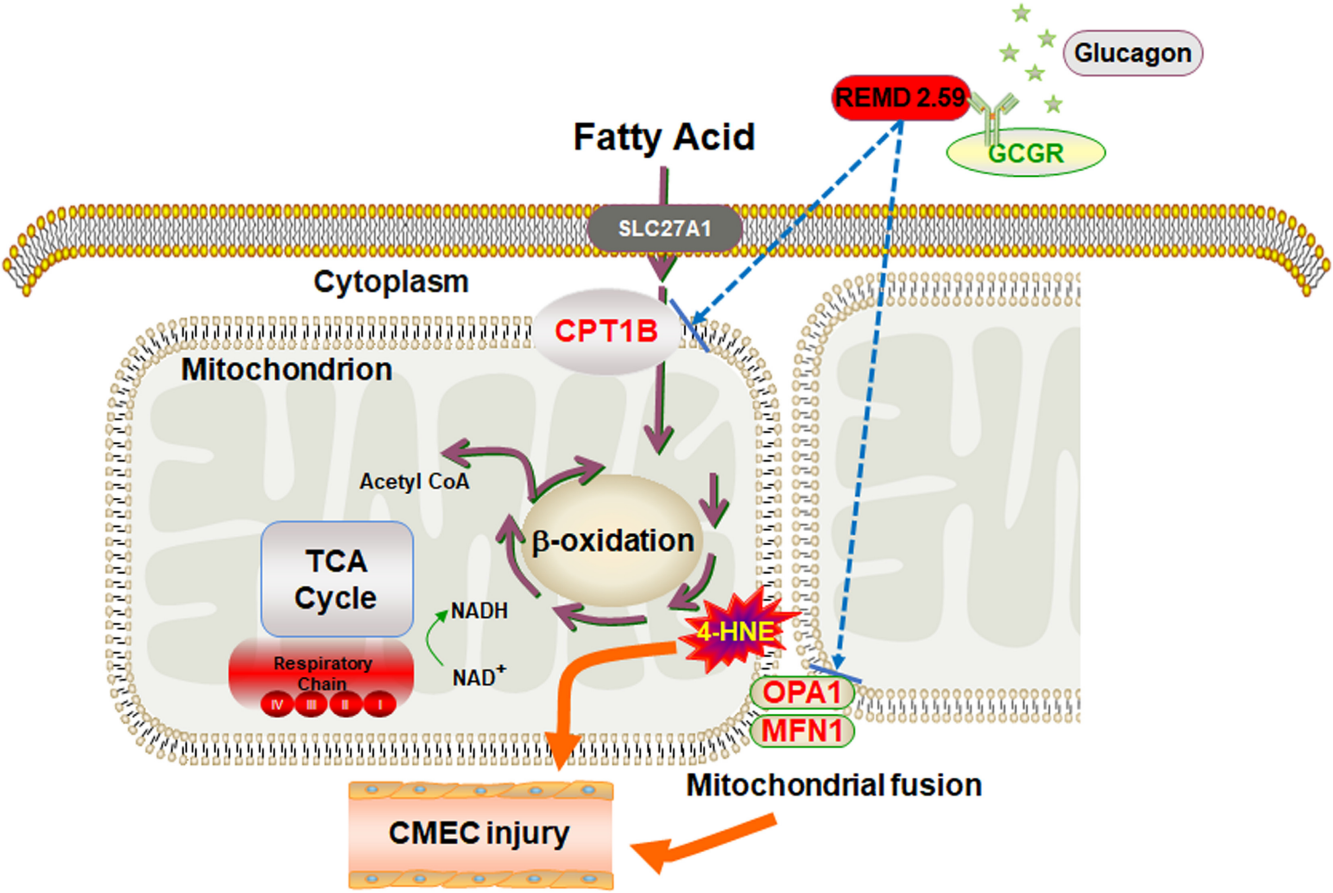
Central illustration. The mitochondrial damage secondary to an increased fatty acid β‐oxidation and mitochondrial fusion can be found in the CMECs of type 2 diabetic mice. REMD 2.59, an antagonistic GCGR mAb, reverses these abnormalities in CMECs and has cardioprotective effects by downregulating the protein levels of CPT1B, OPA1, and MFN1. CMECs, cardiac microvascular endothelial cells; CPT1B, carnitine palmitoyltransferase 1B; GCGR, glucagon receptor; mAb, monoclonal antibody; MFN1, mitofusin‐1; OPA1, optic atrophy type 1.

Taken together, these results suggested that treatment with GCGR mAb could effectively attenuate the diabetes‐induced upregulation of CPT1B, OPA1, and MFN1, and oxidative stress in CMECs (Figure 5).

## 4 DISCUSSION

GCGR blockage can improve cardiac function following a myocardial infarction.^17^, ^18^ However, the underling mechanism remains unclear. In this study, we first showed that REMD 2.59, a fully competitive antagonistic human GCGR mAb, prevented cardiac diastolic dysfunction in *db/db* mice. Second, we found that GCGR mAb significantly increased the number of CMECs in the diabetic mice, indicating that improvement of cardiac microvascular function by GCGR mAb might account for the amelioration of the diabetes‐induced cardiac diastolic dysfunction. Third, we performed TMT‐based quantitative proteomic analysis in isolated primary CMECs to investigate the possible mechanism of this beneficial effect. Bioinformatic analysis on DEPs showed that GCGR mAb downregulated the levels of the proteins enriched in fatty acid β‐oxidation, TCA cycle, and mitochondrial fusion in *db/db* mice. These results suggested that the cardioprotective effects of GCGR mAb might be achieved by improving the energy metabolism‐related process and mitochondrial dynamics in CMECs. Subsequently, we used western blot analysis to confirm that GCGR mAb attenuated the diabetes‐induced upregulation of CPT1B, OPA1, and MFN1 (the proteins that play a key role in energy metabolism and mitochondrial fusion^29^) in CMECs. In addition, we found that GCGR mAb ameliorated oxidative stress, as indicated by a significant reduction in the levels of the 4‐HNE modified protein, in the CMECs of the diabetic mice.

Cardiovascular complications, including diabetic cardiomyopathy, account for more than 80% of diabetic deaths.^2^ Therefore, the cardioprotective effect of novel glucose‐lowering drugs is a hot topic in the field of diabetes therapy. Recently, large‐scale cardiovascular outcomes trials have shown that several glucagon‐like peptide‐1 receptor agonists and sodium‐glucose cotransporter‐2 inhibitors have cardioprotective benefits.^30^, ^31^, ^32^, ^33^ Therefore, the international guidelines recommend use of these two classes of drugs in patients with type 2 diabetes and atherosclerotic cardiovascular disease to reduce the risk of death or cardiovascular events.^34^ However, the effective therapies of diabetic cardiomyopathy are limited and need to be further explored. In this study, we observed a significant abnormality of E/A, E/e′, and myocardial performance index as determined by echocardiography in *db*/*db* mice, whereas the impairment in these cardiac function indices was markedly improved by GCGR mAb treatment. Several recent studies have demonstrated that GCGR mAb improves cardiac function. For instance, GCGR mAb improved cardiac function in diabetic mice via activation of AMP‐activated protein kinase, which was independent of its glucose‐lowering effect.^18^ GCGR mAb enhanced insulin signaling and branched chain amino acid catabolism but inhibited the signaling pathway target of rapamycin to improve cardiac function after myocardial infarction.^17^ GCGR mAb inhibited cardiac hypertrophy and fibrotic remodeling and attenuated contractile dysfunction in the mouse models of myocardial infarction or pressure overload.^16^ In those studies, however, the cardiac function in animal models was severely injured. In this study, we found that in the early stage of diabetic cardiomyopathy, diastolic dysfunction, and metabolic disturbances (such as hyperglycemia) were not accompanied by substantial changes in myocardial structure and systolic function. These results suggested that GCGR mAb treatment could have early protective effects, which might move the treatment window of the disease forward.

Furthermore, our study demonstrated that diabetic mice exhibited a marked reduction in cardiac microvascular density, whereas GCGR mAb treatment significantly attenuated this reduction. Because the decrease and dysfunction of CMECs are closely related to cardiac function,^4^ GCGR mAb may have cardioprotective effects by regulating CMECs. Proteomics is a powerful and versatile tool to elucidate mechanisms dysregulated in disease and contributes to the discovery and validation of novel therapeutic targets.^35^, ^36^, ^37^, ^38^ To investigate the possible mechanism of the GCGR mAb action on CMECs, we performed TMT‐based quantitative proteomic analysis in isolated primary CMECs. The results showed that the fatty acid β‐oxidation process was upregulated in diabetic CMECs. Under normal physiological condition, glycolysis provides most of the adenosine triphosphate in endothelial cells, while fatty acid β‐oxidation only accounts for 5%.^39^ However, the fatty acid β‐oxidation process can be modulated by different conditions. Exposure of the human umbilical vein endothelial cell line to high glucose resulted in an increased palmitate oxidation and elevated reactive oxygen species.^40^ Elevated leptin levels and insulin resistance increased fatty acid β‐oxidation in aortic endothelial cells.^41^, ^42^ This unique metabolic mode can protect endothelial cells from damage caused by mitochondrial electron leakage and reactive chemicals.^24^, ^25^, ^26^ We supposed that restoration of the metabolic mode might be a therapeutic strategy for improving endothelial cell function.

Dysregulated mitochondrial dynamics is crucial in the development of diabetes and its vascular complications.^43^ For instance, the changes in mitochondrial function may precede the reduction in contractility,^44^ and mitochondrial hormesis can prevent the development of vascular complications in type 2 diabetes.^45^ We found that the protein levels of OPA1 and MFN1, which are involved in mitochondrial fusion, were increased in diabetic CMECs. MFN1 and MFN2 are responsible for outer mitochondrial membrane fusion. MFN1 acts inter‐mitochondrially through its HR2 domains,^29^ whereas MFN2 interacts with MFN1 to promote the fusion of outer mitochondrial membrane.^46^ OPA1 orchestrates the fusion of inner mitochondrial membrane and is involved in the maintenance of mitochondrial cristae structure.^47^ Mitochondrial dysfunction is closely related to oxidative stress. When a damaged mitochondrion fuses with a healthy one, the result is not a larger healthy organelle but rather a larger damaged mitochondrion, which could expand the damage by releasing high level of reactive oxygen species.^48^ Therefore, our findings indicated that the abnormal mitochondrial fusion in CMECs might lead to oxidative stress, and the fusion of damaged mitochondria with healthy ones might expand the scope of mitochondrial damage, thereby resulting in the decreased microvascular density and cardiac dysfunction. Of course, it is better to measure the mitochondrial structural features and support the concept of abnormal mitochondrial fusion by using transmission electron microscope.

Abnormal cellular energy metabolism and oxidative stress in diabetes can lead to endoplasmic reticulum stress, cardiomyocyte death or hypertrophy, endothelial cell damage, microvascular dysfunction, and profibrotic responses in inflammatory cells and fibroblasts. Furthermore, these pathological processes can affect each other. This in turn mediates cardiac hypertrophy, fibrosis, and ischemia, thereby resulting in diastolic and systolic dysfunction in diabetic cardiomyopathy.^3^ Although the fine‐tune mechanisms leading to diabetic cardiomyopathy remain partially opaque, restoring cardiac energy metabolism seems to be a cornerstone.^49^ Therefore, our observations in this study may be helpful to find a potential therapeutic strategy for correcting the imbalance between glycolysis and fatty acid β‐oxidation.

One limitation of this study is that we did not include insulin‐treated *db/db* mice as an active control group. Therefore, we could not exclude the possibility that protection of CMECs by GCGR mAb was secondary to its glucose‐lowing effect. We also did not include *db/m* control mice treated with GCGR mAb. Besides, we could not ascertain whether the glucagon signaling had a direct effect on diabetic hearts or CMECs. The in vitro assay on cardiomyocytes or CMECs with GCGR activation or blockage will help answer the question. Another limitation is that we only focused on the role of CMECs in diabetic cardiomyopathy. We believe that other cell types may also play the important roles, and cell–cell interactions are worthy of further investigation. In addition, endothelial cells from different niches exhibit distinct molecular and functional properties, which lead to the heterogeneity of vascular tree. In this study, we investigated only the effect of GCGR mAb on CMECs. Therefore, we could not extent the conclusion directly to other types of endothelial cells. The other limitation is that the proteomic analysis showed the downregulation of many proteins involved in fatty acid β‐oxidation and mitochondrial fusion in GCGR mAb‐treated mice, but the protein expression pattern was confirmed by western blot only for 3 proteins, CPTIB, OPA 1, and MFN‐1. Also, only one oxidative stress marker 4‐HNE was used. Therefore, we could not exclude the possibility that the other important proteins and pathways might be involved in the protective effects of GCGR mAb on CMECs.

In summary, we found that GCGR antagonism had cardioprotective effects possibly through improving energy metabolism and mitochondrial dynamics in CMECs in type 2 diabetic mice. Our findings might provide a rationale for the future clinical development of GCGR mAb in the prevention and treatment of diabetic cardiomyopathy.

## AUTHOR CONTRIBUTIONS

Lingyun Zu and Tianpei Hong designed the research and reviewed the manuscript. Peng Wang and Rui Wei performed the research experiments and statistical analyses and prepared the manuscript. Xiaona Cui and Jin Yang helped perform the research experiments. Zongzhe Jiang helped analyze data.

## DISCLOSURE

The authors have nothing to disclose.

## Supporting information


**DATA S1:** Supporting Information.

**DATA S2:** Supporting Information.

## Data Availability

The data used to support the findings of this study are provided as supplementary files. Any further data can be made available from the corresponding authors upon request.
