# Supplementary material for: Fatty acid β‐oxidation and mitochondrial fusion are involved in cardiac microvascular endothelial cell protection induced by glucagon receptor antagonism in diabetic mice
Source: J Diabetes. 2023 Aug 19;15(12):1081–94. doi: 10.1111/1753-0407.13458 (PMC10755618; doi:10.1111/1753-0407.13458)
Supplement: Supplementary file 1 — DATA S1: Supporting Information. [file JDB-15-1081-s002.pdf]

Fatty acid β-oxidation and mitochondrial fusion are involved in glucagon receptor antagonism induced protection on cardiac microvascular endothelial cells in diabetic mice

Supplementary Methods

1. Echocardiography evaluation

Thirteen mice per group were evaluated by cardiac ultrasound. The VisualSonics high-resolution Vevo 2100 ultrasound system (VisualSonics Inc., Toronto, Canada) with a 30-MHz linear array ultrasound transducer (MS-400, VisualSonics Inc.) was used. Briefly, mice were anesthetized with 2.0% isoflurane (Abbott, Chicago, IL, USA) until the heart rate stabilized at 400 to 500 beats per minute. Parasternal long-axis images were acquired in B-mode with appropriate position of the probe to identify the maximum left ventricular (LV) length. In this view, the M-mode cursor was positioned perpendicular to the maximum and minimum LV dimension in end-diastole and end-systole respectively, and M-mode images were obtained for measuring wall thickness and chamber dimensions. LV ejection fraction (EF) and fractional shortening (FS) were calculated as follows: EF (%) = $100 \times [(LVIDd^3 - LVIDs^3)/LVIDd^3]$; FS (%) = $100 \times [(LVIDd - LVIDs)/LVIDd]$ (LVIDd, left ventricular internal dimension diastolic; LVIDs, left ventricular internal dimension systolic). The apical four-chamber view was acquired and the peak flow velocities during early diastole (E wave) and late diastole (A wave) across the mitral valve, as well as early-diastolic peak velocity (e') of the mitral valve ring were measured. E/e' and E/A ratios, which reflected the LV diastolic

function, were calculated. The myocardial performance index (MPI) was calculated by the formula: ([ICT + IRT]/ET), where ICT represents isovolumic contraction time, IRT denotes isovolumic relaxation time, and ET represents ejection time.

2. Histological analysis

Four mice per group were subjected to histological analysis. Sections were stained with Masson's and Sirius red staining (Sevicebio, Wuhan, China) for detection of cardiac fibrosis, and stained with Hematoxylin & Eosin (HE) staining for measurement of cardiomyocyte size ex vivo. Images were captured using a Nikon microscope (Eclipse E100; Nikon, Tokyo, Japan). To quantify cardiac fibrosis, 10 fields were randomly selected per section with 3 cardiac sections per mouse. Cardiac fibrosis was calculated as the percentage of Masson's and Sirius red staining-positive area to total myocardial area. Similar methods were used to evaluate cardiac hypertrophy by calculation of the average cardiomyocyte area in HE staining heart sections.

For immunofluorescent staining, sections were prepared and incubated with primary antibodies at 4°C overnight and secondary antibodies for 1 h at room temperature, followed by staining with 1 μg/mL of 4',6-diamidino-2-phenylindole (Sigma-Aldrich, St. Louis, MO, USA). The antibodies were as follows: rabbit polyclonal anti-CD31 (1:50; Abcam, Cambridge, UK), rabbit polyclonal anti-intercellular cell adhesion molecule-1 (ICAM-1, 1:400; Sevicebio), rabbit monoclonal anti-vascular cell adhesion molecule-1 (VCAM-1, 1:2,000; Abcam); 594-conjugated goat polyclonal anti-rabbit secondary antibody or 488-conjugated goat polyclonal anti-

rabbit secondary antibody (both 1:400; Sevicebio). Images were captured by a Nikon microscope (Eclipse C1; Nikon). Ten fields were randomly selected per section with 3 cardiac sections per mouse. The relative microvascular density was evaluated by calculating the CD31-positive capillary numbers in the cardiac sections. Inflammatory responses were indicated by the proportion of ICAM-1- or VCAM-1-positive cells.

Cell apoptosis was determined by using terminal deoxynucleotidyl transferase-mediated deoxyuridine triphosphate-nick end labelling (TUNEL) assay (Roche, Basel, Switzerland) according to the manufacturer's instructions. The images were obtained using an inverted microscope (Eclipse C1; Nikon) equipped with a digital imaging camera (Nikon). To quantify the cell apoptosis, 10 fields were randomly selected per section with 3 cardiac sections per mouse. The cell apoptosis was calculated as the percentage of TUNEL staining-positive cell number to total cell number.

3. Isolation of primary CMECs from mouse heart tissues

Briefly, mice were anesthetized, and heart was excised aseptically and transferred to ice-cold D-Hank's solution (free of Ca^{2+}, Mg^{2+} and phenol red) (coolaber, Beijing, China). The tissues were minced finely, followed by digestion in 150 U/mL warm collagenase II (Worthington Biochemical Corp., Lakewood, NJ, USA), 150 U/mL collagenase IV (Worthington Biochemical Corp.), 1.2 U/mL Dispase (Worthington Biochemical Corp.) and 50 U/ml DNase I (Roche) at 37°C for 45 min with gentle agitation. The single cell suspension was passed through a 70-μm cell strainer and subjected to centrifugation. The cell pellet was resuspended in red blood cell lysis buffer,

lysed for 5 min and subjected to centrifugation. Subsequently, the single cell suspension was passed through a 40-μm cell strainer and subjected to centrifugation. The number of cells was approximately 2×10^7 from every 3 mice. The cell pellet was resuspended in buffer and incubated with anti-mouse CD31 MicroBeads (Miltenyi Biotec, Bergisch Gladbach, Germany) for 15 min at 4°C. The buffer was prepared following the manufacturer's instructions. After separation in a magnetic separator (Miltenyi Biotec), the cells were collected and resuspended in phosphate buffer solution for the following studies. The number of CMECs was approximately 2.5×10^6 from every 3 mice. The primary CMECs were directly used for subsequent proteomic analysis and western blot, without being cultured and passaged.

4. Proteomic analysis

Protein extraction:

Sample was sonicated three times on ice using a high intensity ultrasonic processor (Scientz, Ningbo, China) in lysis buffer (8 mol/L urea and 1% protease inhibitor cocktail; Merck Millipore). The remaining debris was removed by centrifugation at 12,000 g at 4 °C for 10 min. Subsequently, the supernatant was collected, and the protein concentration was determined by bicinchoninic acid protein assay method (Beyotime Biotechnology, Shanghai, China).

Trypsin digestion and TMT labeling:

For digestion, the protein solution was reduced with 5 mmol/L dithiothreitol (Sigma-Aldrich) for 30 min at 56°C and alkylated with 11 mmol/L iodoacetamide (Sigma-

Aldrich) for 15 min at room temperature in darkness. The protein sample was then diluted by adding 100 mmol/L tetraethyl ammonium bromide (Sigma-Aldrich) to urea concentration less than 2 mol/L. Subsequently, trypsin (Promega, Madison, WI, USA) was added at 1:50 trypsin-to-protein mass ratio for the first digestion overnight and 1:100 trypsin-to-protein mass ratio for a second 4-h digestion. After trypsin digestion, peptide was processed by using TMT kit (6 plex) (Thermo Fisher Scientific, Waltham, MA, USA) according to the manufacturer's protocol. Briefly, one unit of TMT reagent were thawed and reconstituted in acetonitrile (Thermo Fisher Scientific). The peptide mixtures were then incubated for 2 h at room temperature, and pooled, desalted and dried by vacuum centrifugation.

Liquid chromatography tandem mass spectrometry analysis:

The column was Agilent 300Extend C18 (4.6 mm x 250 mm, particle size 5 μm) (Agilent, Santa Clara, CA, USA). The peptides were dissolved in phase A of liquid chromatography mobile phase and separated using EASY-nLC 1000 Ultra High-Performance Liquid Chromatography (UHPLC) (Thermo Fisher Scientific) system. Mobile phase A was an aqueous solution containing 0.1% formic acid (Sigma-Aldrich) and 2% acetonitrile (Thermo Fisher Scientific), and mobile phase B was an aqueous solution containing 0.1% formic acid and 90% acetonitrile. Liquid Gradient Settings: 38 min, 8-23B; 14 min, 23-35B; 4 min, 35-80B; 4 min, 80B; flow maintained at 550 nL/min. The peptides were separated by an UHPLC system and injected into the nano-electrospray ion NSI ion source for ionization and then analyzed by Q exactive plus (Thermo Fisher Scientific) Mass Spectrometry (MS). The ion source voltage is set to

2.2 kV, peptide precursor ions and their secondary fragments were analyzed using high-resolution Orbitrap (Thermo Fisher Scientific). The scanning range of the primary mass spectrometer is set to 400-1,500 m/z, and the scanning resolution is set to 70,000. The data acquisition mode uses the data-dependent scanning program, that is, after the first-level scan, the first 20 peptide precursor ions with the highest signal intensity are selected and sequentially entered into the higher collisional dissociation collision cell for fragmentation using 28% of the fragmentation energy MS analysis. The automatic gain control was set to 5E4, the signal threshold was set to 3.8E4 ions/s, the maximum injection time was set to 50 ms, and the dynamic exclusion time of tandem MS scanning was set to 30 s to avoid repeated scans of precursor ions.

Database Search:

The resulting tandem MS data were processed using Maxquant search engine (v.1.5.2.8; Max-Planck-Institute of Biochemistry, Martinsried, Germany). Tandem mass spectra were searched against human UniProt database concatenated with reverse decoy database. Trypsin/P was specified as cleavage enzyme allowing up to 4 missing cleavages. The mass tolerance for precursor ions was set as 20 ppm in first search and 5 ppm in main search, and the mass tolerance for fragment ions was set as 0.02 Da. Carbamidomethyl on cysteine was specified as fixed modification and acetylation modification and oxidation on methionine were specified as variable modifications. The false discovery rate was adjusted to < 1% and minimum score for modified peptides was set > 40. The differentially expressed proteins (DEPs) between two groups were analyzed by unpaired student *t*-test. The DEPs were filtered with criteria of $P < .05$, and

cut-off fold change > 1.3 with at least three biological replicates.

Gene Ontology (GO) annotation proteome was derived from the UniProt-GOA database (http://www.ebi.ac.uk/GOA/). Kyoto Encyclopedia of Genes and Genomes (KEGG) pathway annotation: KEGG connects known information on molecular interaction networks, such as pathways and complexes (the “Pathway” database), information about genes and proteins generated by genome projects (including the gene database) and information about biochemical compounds and reactions (including compound and reaction databases). These databases are different networks, known as the “protein network” and “chemical universe”, respectively. There are efforts in progress to add to the knowledge of KEGG, including information regarding ortholog clusters in the KEGG Orthology database. For each category, a two-tailed Fisher’s exact test was employed to test the enrichment of DEPs, and a corrected $P < .05$ was considered significant. For further hierarchical clustering of functional classification (such as signaling pathway, biological process, molecular function, and cellular component), we first collated all the categories obtained after enrichment along with their P values, and then filtered for those categories which were at least enriched in one of the clusters with P value < .05. This filtered P value matrix was transformed by the function $X = -\log_{10}$ (P value). Subsequently, these X values were z-transformed for each functional category. These z scores were clustered by one-way hierarchical clustering (Euclidean distance, average linkage clustering) in Genesis. Cluster membership was visualized by a heat map using the “heatmap.2” function from the “gplots” R-package.

Supplementary Figures

Fig. S1

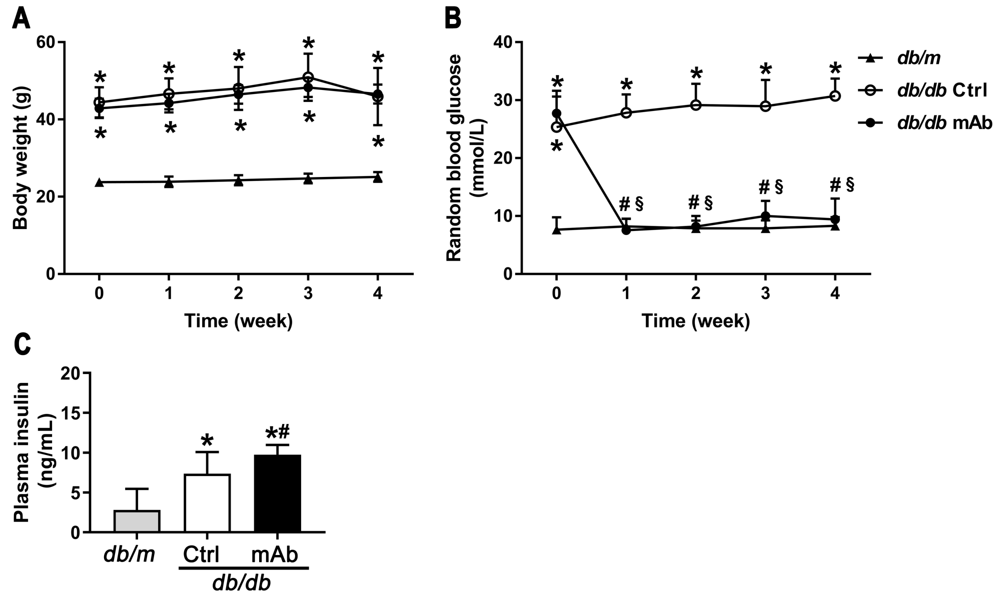

Supplementary Fig. 1. Metabolic parameters in *db/db* mice treated with GCGR mAb or IgG (as control) for 4 weeks. An antagonistic GCGR mAb, REMD 2.59 (5 mg/kg), or IgG was intraperitoneally administrated once a week in male *db/db* mice. Age-matched male *db/m* mice were included as the normal controls. (A) Body weight. (B) Random blood glucose. (C) Fasting plasma insulin. n = 13 mice per group. Data are expressed as the mean ± S.D. Statistical analysis was conducted by two-way or one-way ANOVA followed by the *post-hoc* Tukey-Kramer test. **P* < .05 *vs. db/m*; $^{\#}P$ < .05 *vs. db/db* Ctrl; $^{\S}P$ < .05 *vs.* pretreatment in the same group.

Abbreviations: Ctrl, control; GCGR, glucagon receptor; mAb, monoclonal antibody; IgG, immunoglobulin G.

Fig. S2

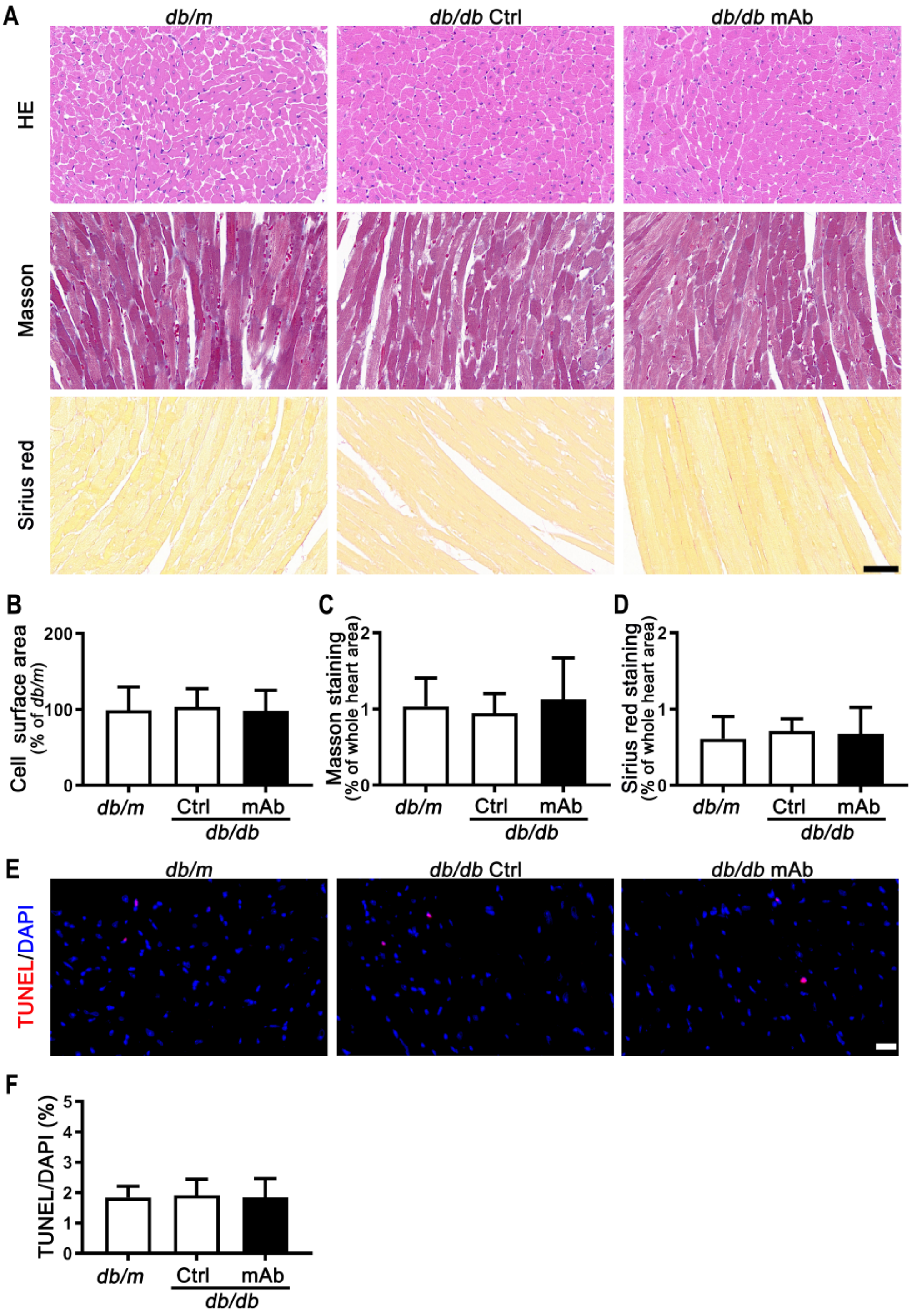

Supplementary Fig. 2. Histological analysis in *db/db* mice treated with GCGR mAb or IgG (as control) for 4 weeks. GCGR mAb (5 mg/kg) or IgG was intraperitoneally administrated once a week in male *db/db* mice. Age-matched male *db/m* mice were included as the normal controls. (A) Representative photograph of

HE staining, Masson's staining and Sirius red staining. Scale bar = 50 μm. (B-C) Quantification of cardiomyocyte size in HE staining (B), and myocardial fibrosis in Masson's staining (C) and Sirius red staining (D). (E) Representative photograph of TUNEL staining. Scale bar = 20 μm. (F) Quantification of TUNEL staining-positive apoptotic cells. n = 4 mice per group. Data are expressed as the mean ± S.D. Statistical analysis was conducted by one-way ANOVA followed by the *post-hoc* Tukey-Kramer test.

Abbreviations: Ctrl, control; GCGR, glucagon receptor; mAb, monoclonal antibody; IgG, immunoglobulin G; HE, Hematoxylin & Eosin; TUNEL, terminal deoxynucleotidyl transferase-mediated deoxyuridine triphosphate-nick end labelling; DAPI, 4',6-diamidino-2-phenylindole.

Fig. S3

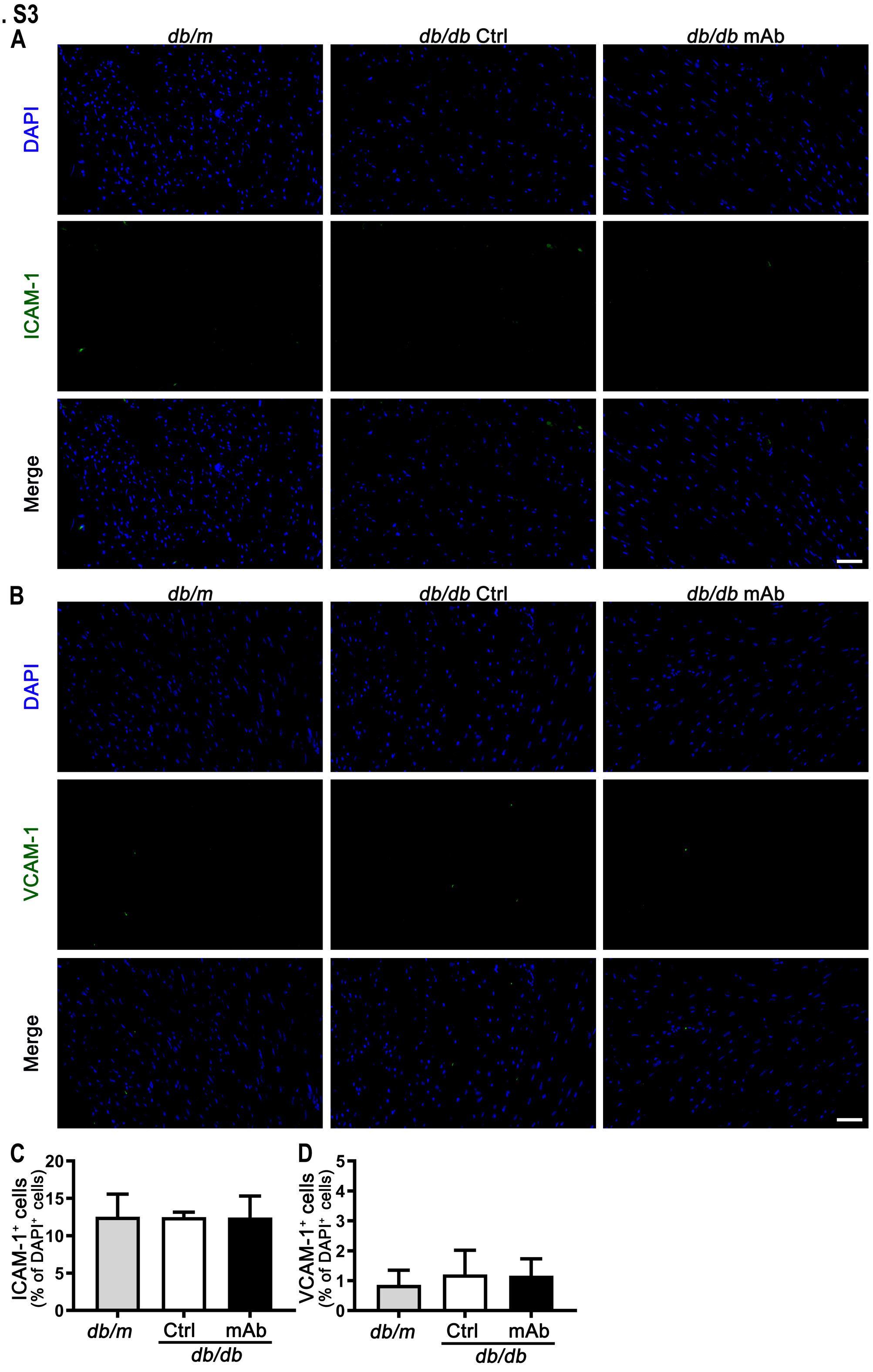

Supplementary Fig. 3. Evaluation of inflammatory responses in the heart tissues of *db/db* mice treated with GCGR mAb or IgG (as control) for 4 weeks. GCGR mAb (5 mg/kg) or IgG was intraperitoneally administrated once a week in male *db/db*

mice. Age-matched male *db/m* mice were included as the normal controls. (A) Representative images of ICAM-1 positive cells. (B) Representative images of VCAM-1 positive cells. Scale bar = 50 μm. (C) Quantification of ICAM-1 positive cells. (D) Quantification of VCAM-1 positive cells. n = 4 mice per group. Data are expressed as the mean ± S.D. Statistical analysis was conducted by one-way ANOVA followed by the *post-hoc* Tukey-Kramer test.

Abbreviations: Ctrl, control; GCGR, glucagon receptor; mAb, monoclonal antibody; IgG, immunoglobulin G; DAPI, 4',6-diamidino-2-phenylindole; ICAM-1, intercellular cell adhesion molecule-1; VCAM-1, vascular cell adhesion molecule-1.
