# Supplementary material for: Fatty acid β‐oxidation and mitochondrial fusion are involved in cardiac microvascular endothelial cell protection induced by glucagon receptor antagonism in diabetic mice
Source: J Diabetes. 2023 Aug 19;15(12):1081–94. doi: 10.1111/1753-0407.13458 (PMC10755618; doi:10.1111/1753-0407.13458)
Supplement: Supplementary file 2 — DATA S2: Supporting Information. [file JDB-15-1081-s001.pdf]

Supplementary Table 1. Echocardiographic characteristics *in db/db* mice treated with GCGR mAb or IgG (as control) for 4 weeks.

Parameter	*db/m*	*db/db* Ctrl	*db/db* mAb
E (mm/s)	573.7 ± 6.37	652.1 ± 15.3	593.2 ± 8.18
A (mm/s)	449.9 ± 5.43	353.9 ± 6.81*	374.6 ± 3.97
e' (mm/s)	−19.1 ± 0.31	−13.5 ± 0.28*	−17.7 ± 0.24#
E/A	1.28 ± 0.10	1.85 ± 0.27*	1.58 ± 0.12#
E/e'	−30.6 ± 4.28	−49.0 ± 10.4*	−33.9 ± 5.51#
MPI	0.48 ± 0.04	0.64 ± 0.19*	0.42 ± 0.04#
LVIDd (mm)	3.63 ± 0.31	3.71 ± 0.44	3.38 ± 0.48
LVIDs (mm)	1.78 ± 0.30	1.90 ± 0.45	1.58 ± 0.58
Ves (µl)	9.92 ± 0.34	12.3 ± 0.71	8.85 ± 0.81
Ved (µl)	56.1 ± 0.93	59.7 ± 1.91	48.9 ± 1.66
EF (%)	82.7 ± 0.44	80.6 ± 0.74	84.6 ± 1.01
FS (%)	51.1 ± 0.50	49.2 ± 0.88	54.4 ± 0.11

Data are expressed as the mean ± S.D. *P < 0.05 *vs. db/m*; #P < 0.05 *vs. db/db* Ctrl. EF (%) = 100 × [($LVIDd^3$ – $LVIDs^3$)/$LVIDd^3$]; FS (%) = 100 × [(LVIDd – LVIDs)/LVIDd]. Abbreviations: E, the peak flow velocities during early diastole; A, the peak flow velocities during late diastole; e', early-diastolic peak velocity; MPI, myocardial performance index; LVIDd, left ventricular internal dimension diastolic; LVIDs, left ventricular internal dimension systolic; Ves, end-systolic volume; Ved, end-diastolic volume; EF, ejection fraction; FS, fractional shortening; GCGR, glucagon receptor; mAb, monoclonal antibody; IgG, immunoglobulin G; Ctrl, control.

Supplementary Table 2. Differentially expressed proteins in CMECs between *db/db* mice and *db/m* mice

Protein accession	Protein description	Gene name	Ratio (*db/db* vs. *db/m*)	P value (*db/db* vs. *db/m*)	Regulated type	*db/db* #1	*db/db* #2	*db/db* #3	*db/m* #1	*db/m* #2	*db/m* #3
Q8R0Y6	Cytosolic 10-formyltetrahydrofolate dehydrogenase	Aldh1l1	0.695	1.6566E-05	Down	0.79	0.758	0.785	1.134	1.105	1.118
P28843	Dipeptidyl peptidase 4	Dpp4	1.306	0.000629927	Up	1.022	1.086	1.023	0.825	0.799	0.773
Q8BTC1	Uncharacterized protein C12orf73 homolog	--	0.605	0.025543095	Down	0.818	1.006	0.68	1.537	1.462	1.14
Q60952	Centrosome-associated protein CEP250	Cep250	1.361	0.003880141	Up	1.27	1.28	1.11	0.925	0.86	0.905
Q8K3W3	Protein CASC3	Casc3	1.333	0.011902906	Up	1.135	1.17	1.281	0.936	0.801	0.953
Q8BIF0	CD99 antigen-like	Cd99l2	0.493	0.000193929	Down	0.763	0.83	0.69	1.557	1.518	1.553
Q99LB2	Dehydrogenase/reductase SDR family member 4	Dhrs4	1.306	4.11811E-05	Up	1.207	1.182	1.232	0.921	0.915	0.936
Q8BHG9	CGG triplet repeat-binding protein 1	Cggbp1	0.703	0.00030619	Down	0.916	0.843	0.868	1.284	1.246	1.207
Q8BJF9	Charged multivesicular body protein 2b	Chmp2b	0.731	0.004343739	Down	0.912	0.787	0.915	1.15	1.238	1.186
P01029	Complement C4-B	C4b	2.379	0.000816035	Up	1.321	1.202	1.471	0.48	0.582	0.617
O08791	Transcription factor	Ebf3	0.755	0.008102351	Down	0.91	0.842	0.817	1.097	1.241	1.063
Q61908	Cx9C motif-containing protein 4	Cmc4	0.737	0.00400039	Down	1.036	0.877	0.987	1.347	1.31	1.279
Q8BMS1	Trifunctional enzyme subunit alpha,	Hadha	1.511	2.97271E-07	Up	1.221	1.236	1.235	0.807	0.819	0.818
P55096	ATP-binding cassette sub-family D member 3	Abcd3	1.53	0.000436169	Up	1.338	1.232	1.374	0.871	0.822	0.885
E9Q4F7	Ankyrin repeat domain-containing protein 11	Ankrd11	1.326	0.001081115	Up	1.287	1.173	1.28	0.96	0.946	0.914
P05125	Natriuretic peptides A	Nppa	1.642	0.000134088	Up	1.289	1.25	1.312	0.74	0.825	0.78
Q64444	Carbonic anhydrase 4	Ca4	1.374	7.21188E-06	Up	1.18	1.149	1.165	0.859	0.838	0.846
P43023	Cytochrome c oxidase subunit 6A2,	Cox6a2	1.481	5.35536E-05	Up	1.267	1.294	1.333	0.849	0.888	0.892
Q9QYR9	Acyl-coenzyme A thioesterase 2, mitochondrial	Acot2	1.529	1.78136E-05	Up	1.265	1.31	1.331	0.836	0.861	0.858
Q3V132	ADP/ATP translocase 4	Slc25a31	1.344	0.000314218	Up	1.15	1.198	1.187	0.916	0.854	0.86
Q6PIE5	Sodium/potassium-transporting ATPase subunit alpha-2	Atp1a2	1.421	3.60266E-05	Up	1.013	1.022	1.037	0.71	0.708	0.744
P45952	Medium-chain specific acyl-CoA dehydrogenase,	Acadm	1.575	3.21027E-06	Up	1.265	1.309	1.309	0.816	0.829	0.821
Q9CR21	Acyl carrier protein, mitochondrial	Ndufab1	1.331	0.000121649	Up	1.17	1.226	1.188	0.883	0.922	0.888
Q99NB1	Acetyl-coenzyme A synthetase 2-like, mitochondrial	Acss1	1.702	2.58745E-05	Up	1.247	1.312	1.296	0.758	0.778	0.729
P63254	Cysteine-rich protein 1	Crip1	0.653	0.001059914	Down	0.896	0.773	0.881	1.284	1.27	1.352
P08121	Collagen alpha-1(III)	Col3a1	2.425	0.000887969	Up	1.223	1.475	1.418	0.639	0.576	0.482
P21460	Cystatin-C	Cst3	1.64	0.002833986	Up	1.204	1.409	1.237	0.84	0.812	0.695
P62897	Cytochrome c, somatic	Cycs	1.831	6.39084E-06	Up	1.49	1.461	1.461	0.79	0.833	0.787
P24788	Cyclin-dependent kinase 11B	Cdk11b	0.718	0.003459997	Down	0.764	0.871	0.809	1.197	1.054	1.152
Q924X2	Carnitine O-palmitoyltransferase 1, muscle isoform	Cpt1b	2.037	5.45814E-06	Up	1.518	1.473	1.581	0.742	0.742	0.76
Q569Z5	Probable ATP-dependent RNA helicase	Ddx46	0.717	0.000499348	Down	0.868	0.857	0.789	1.153	1.155	1.197
O70133	ATP-dependent RNA helicase A	Dhx9	0.56	1.4793E-06	Down	0.745	0.755	0.749	1.352	1.305	1.356
Q78IK2	ATP synthase membrane subunit DAPIT, mitochondrial	Atp5md	1.324	0.000848637	Up	1.28	1.152	1.219	0.92	0.929	0.908
P97929	Breast cancer type 2 susceptibility protein homolog	Brca2	2.403	0.002217477	Up	1.454	1.696	1.526	0.517	0.78	0.649
Q91WN1	DnaJ homolog subfamily C member 9	Dnajc9	0.591	0.005591905	Down	0.66	0.878	0.883	1.343	1.422	1.329
Q9DCR2	AP-3 complex subunit sigma-1	Ap3s1	0.748	0.000300948	Down	0.846	0.824	0.868	1.099	1.174	1.12
A2BH40	AT-rich interactive domain-containing	Arid1a	0.745	0.005131633	Down	0.882	0.749	0.865	1.131	1.132	1.086
P53996	Cellular nucleic acid-binding protein	Cnbp	0.667	0.000236909	Down	0.888	0.83	0.817	1.319	1.239	1.245

Q8K009	Mitochondrial 10-formyltetrahydrofolate dehydrogenase	Aldh1l2	1.405	0.003036257	Up	1.068	1.208	1.24	0.826	0.801	0.875
Q9CY21	Probable 18S rRNA (guanine-N(7))-methyltransferase	Bud23	0.606	0.000977629	Down	0.835	0.89	0.759	1.273	1.423	1.4
Q9JLV1	BAG family molecular chaperone regulator 3	Bag3	0.726	0.000104194	Down	0.872	0.903	0.891	1.268	1.195	1.209
Q9Z0X1	Apoptosis-inducing factor 1, mitochondrial	Aifm1	1.857	7.88256E-05	Up	1.402	1.416	1.534	0.769	0.819	0.756
Q8C033	Rho guanine nucleotide exchange factor 10	Arhgef1(	0.738	0.017123499	Down	0.852	0.836	0.849	1.31	1.118	1.01
Q8BGS2	BolA-like protein 2	Bola2	0.707	0.002016431	Down	0.858	0.775	0.736	1.127	1.078	1.148
D3Z6Q9	Bridging integrator 2	Bin2	0.333	4.6421E-05	Down	0.652	0.552	0.564	1.845	1.777	1.687
P05202	Aspartate aminotransferase,	Got2	1.368	7.39836E-07	Up	1.24	1.22	1.238	0.898	0.906	0.899
Q8VCT3	Aminopeptidase B	Rnpep	0.749	0.000679881	Down	0.862	0.912	0.84	1.205	1.151	1.134
Q8BW75	Amine oxidase [flavin-containing] B	Maob	1.655	1.41212E-05	Up	1.362	1.338	1.38	0.834	0.793	0.838
Q8BGN9	Uncharacterized protein C1orf115 homolog	--	1.394	0.01426633	Up	0.999	1.016	1.108	0.641	0.813	0.787
Q64152	Transcription factor	Btf3	0.714	0.001272341	Down	0.84	0.866	0.758	1.152	1.172	1.127
Q8CDM4	Coiled-coil domain-containing protein 73	Ccdc73	1.763	0.000145613	Up	1.202	1.35	1.303	0.756	0.704	0.727
P23198	Chromobox protein homolog 3	Cbx3	0.75	0.010784104	Down	0.891	0.895	0.796	1.262	1.063	1.119
E9Q7D5	Rho guanine nucleotide exchange factor 5	Arhgef5	0.766	0.002836999	Down	0.822	0.837	0.93	1.095	1.143	1.142
Q921H9	Cytochrome c oxidase assembly factor 7	Coa7	1.332	0.002253847	Up	1.216	1.257	1.137	0.903	0.859	0.949
P08122	Collagen alpha-2(IV)	Col4a2	0.765	0.001205572	Down	0.873	0.809	0.816	1.041	1.108	1.116
P63154	Crooked neck-like protein 1	Crnkl1	0.725	0.000538893	Down	0.828	0.843	0.831	1.085	1.159	1.208
P07758	Alpha-1-antitrypsin 1-1	Serpina1	0.631	0.001626534	Down	0.904	0.872	0.811	1.348	1.254	1.497
Q9EPL9	Peroxisomal acyl-coenzyme A oxidase 3	Acox3	2.329	1.53328E-05	Up	1.408	1.408	1.446	0.605	0.647	0.578
Q9Z0X0	Cdc42 effector protein 5	Cdc42ep	0.669	0.047820902	Down	0.629	1.012	0.917	1.268	1.358	1.199
Q8BH59	Calcium-binding mitochondrial carrier protein Aralar1	Slc25a12	1.332	4.38164E-05	Up	1.229	1.224	1.247	0.934	0.943	0.901
Q9R0L7	A-kinase anchor protein 8-like	Akap8l	0.763	0.006804225	Down	0.923	0.913	0.841	1.116	1.12	1.274
P08074	Carbonyl reductase [NADPH] 2	Cbr2	0.592	1.64514E-05	Down	0.732	0.684	0.72	1.197	1.218	1.193
Q80ZM8	Cardiolipin synthase (CMP-forming)	Crls1	0.765	0.023914904	Down	1.007	0.929	0.819	1.126	1.163	1.311
Q99L13	3-hydroxyisobutyrate dehydrogenase, mitochondrial	Hibadh	1.462	6.49761E-05	Up	1.27	1.297	1.248	0.903	0.856	0.851
Q9JIX8	Apoptotic chromatin condensation inducer in the nucleus	Acin1	0.719	0.000378731	Down	0.85	0.854	0.845	1.204	1.114	1.227
P62737	Actin, aortic smooth	Acta2	1.417	0.011111064	Up	1.104	1.126	1.244	0.71	0.849	0.893
D3YVF0	A-kinase anchor protein	Akap5	0.512	4.86448E-05	Down	0.724	0.793	0.71	1.417	1.464	1.469
Q06185	ATP synthase subunit e, mitochondrial	Atp5me	1.332	0.003290842	Up	1.206	1.338	1.209	0.887	0.946	0.984
O70305	Ataxin-2	Atxn2	0.645	0.000488643	Down	0.847	0.794	0.894	1.283	1.272	1.376
Q8BJE2	Butyrophilin-like protein	Btnl9	1.956	1.31866E-05	Up	1.31	1.378	1.325	0.658	0.707	0.687
P46414	Cyclin-dependent kinase inhibitor 1B	Cdkn1b	0.639	0.000841999	Down	0.805	0.918	0.82	1.253	1.369	1.358
O89053	Coronin-1A	Coro1a	0.456	5.13925E-05	Down	0.644	0.611	0.565	1.279	1.361	1.35
Q9WVG6	Histone-arginine methyltransferase	Carm1	0.708	0.018499608	Down	0.752	0.921	0.813	1.049	1.322	1.141
Q8VHY0	Chondroitin sulfate proteoglycan 4	Cspg4	1.322	0.016539599	Up	1.038	1.227	1.003	0.774	0.854	0.844
Q9CXX9	CUE domain-containing protein 2	Cuedc2	1.647	0.000314414	Up	1.62	1.403	1.482	0.924	0.915	0.897
P70677	Caspase-3	Casp3	0.687	0.000396062	Down	0.79	0.792	0.729	1.08	1.161	1.122
Q8VDP2	UPF0428 protein CXorf56 homolog	--	0.681	0.001590161	Down	0.764	0.889	0.849	1.281	1.212	1.183
Q9R0H0	Peroxisomal acyl-coenzyme A oxidase 1	Acox1	1.901	1.80746E-05	Up	1.41	1.428	1.439	0.737	0.724	0.789
P41216	Long-chain-fatty-acid--CoA ligase 1	Acsl1	1.831	7.02186E-08	Up	1.453	1.449	1.443	0.789	0.784	0.8
Q9CZS1	Aldehyde dehydrogenase X, mitochondrial	Aldh1b1	1.651	0.001260791	Up	1.144	1.308	1.384	0.749	0.812	0.762
P29452	Caspase-1	Casp1	0.626	0.002494395	Down	0.655	0.73	0.794	1.103	1.258	1.12

O08532	Voltage-dependent calcium channel subunit alpha-2/delta-1	Cacna2d	1.359	1.08567E-06	Up	1.09	1.094	1.096	0.811	0.795	0.808
Q9CQI6	Coactosin-like protein	Cotl1	0.719	0.000333112	Down	0.695	0.722	0.656	0.947	0.972	0.966
Q9D385	ADP-ribosylation factor-like protein 2-binding protein	Arl2bp	0.703	0.002060596	Down	0.776	0.786	0.838	1.099	1.238	1.078
Q8R2Q8	Bone marrow stromal antigen 2	Bst2	0.426	0.000224093	Down	0.739	0.774	0.631	1.62	1.768	1.641
Q8VIH7	Cysteine and tyrosine-rich protein 1	Cyyr1	1.474	0.011435542	Up	1.212	1.341	1.024	0.866	0.767	0.793
Q8BXV2	BRI3-binding protein	Bri3bp	1.793	0.001078611	Up	1.528	1.488	1.574	0.848	0.956	0.756
Q00898	Alpha-1-antitrypsin 1-5	Serpina1	0.165	1.2084E-05	Down	0.382	0.415	0.33	2.32	2.222	2.286
P50544	Very long-chain specific acyl-CoA dehydrogenase,	Acadvl	1.687	1.31179E-06	Up	1.336	1.305	1.346	0.778	0.792	0.794
Q9CZW4	Long-chain-fatty-acid--CoA ligase 3	Acsl3	1.452	0.006051837	Up	1.124	1.356	1.162	0.898	0.826	0.785
B2RRD7	Peregrin	Brpf1	0.644	0.002466343	Down	0.879	0.741	0.841	1.259	1.371	1.194
P01900	H-2 class I histocompatibility antigen, D-D alpha	H2-D1	0.483	1.80588E-05	Down	0.737	0.81	0.744	1.566	1.592	1.583
Q61881	DNA replication licensing factor MCM7	Mcm7	0.705	0.001426086	Down	0.819	0.908	0.836	1.192	1.157	1.286
Q3KNM2	E3 ubiquitin-protein ligase MARCHF5	Marchf5	1.465	0.037178093	Up	1.032	1.514	1.138	0.831	0.79	0.893
Q99ME9	Nucleolar GTP-binding protein 1	Gtpbp4	0.475	0.010756071	Down	0.872	0.583	0.58	1.54	1.581	1.162
O88491	Histone-lysine N-methyltransferase, H3 lysine-36 specific	Nsd1	0.756	0.033903842	Down	0.755	0.907	0.68	1.028	0.977	1.094
P68134	Actin, alpha skeletal	Acta1	0.557	2.93116E-05	Down	0.845	0.829	0.796	1.417	1.509	1.512
P70180	Atrial natriuretic peptide receptor 3	Npr3	1.416	0.011121845	Up	1.196	1.166	1.014	0.818	0.86	0.707
P18242	Cathepsin D	Ctsd	1.316	7.82667E-06	Up	1.083	1.078	1.078	0.834	0.808	0.819
P22907	Porphobilinogen	Hmbs	0.744	0.02571706	Down	0.917	0.706	0.844	1.101	1.182	1.032
P01837	Immunoglobulin kappa constant	Igkc	0.537	0.004782413	Down	0.885	0.633	0.851	1.559	1.472	1.381
Q91VC3	Eukaryotic initiation factor 4A-III	Eif4a3	0.708	0.000292985	Down	0.808	0.893	0.861	1.201	1.209	1.211
Q8CAS9	Protein mono-ADP-ribosyltransferase	Parp9	0.639	0.000220094	Down	0.854	0.879	0.806	1.346	1.366	1.262
Q8BSQ9	Protein polybromo-1	Pbrm1	0.751	0.001721365	Down	0.911	0.837	0.808	1.133	1.106	1.163
Q08857	Platelet glycoprotein 4	Cd36	1.493	1.66769E-06	Up	1.208	1.188	1.182	0.809	0.793	0.794
Q99M07	Cytochrome c oxidase assembly factor 5	Coa5	0.719	0.000748128	Down	0.883	0.939	0.999	1.303	1.315	1.308
O09172	Glutamate--cysteine ligase regulatory subunit	Gclm	0.736	0.00275691	Down	0.855	0.81	0.748	1.064	1.065	1.151
O54824	Pro-interleukin-16	Il16	0.686	0.000746922	Down	0.883	0.913	0.819	1.209	1.311	1.29
Q91YJ5	Translation initiation factor IF-2,	Mtif2	0.744	0.014389864	Down	0.986	0.904	0.802	1.254	1.112	1.254
O35368	Interferon-activable protein 203	Ifi203	0.553	0.000493459	Down	0.766	0.837	0.726	1.467	1.451	1.296
Q69ZQ2	Pre-mRNA-splicing factor ISY1 homolog	Isy1	0.688	0.020724055	Down	0.932	0.959	0.714	1.302	1.324	1.16
Q9Z2D6	Methyl-CpG-binding protein 2	Mecp2	0.495	2.14427E-05	Down	0.77	0.8	0.724	1.567	1.516	1.554
A2AJI0	MAP7 domain-containing protein 1	Map7d1	0.642	0.001768959	Down	0.862	0.876	0.77	1.222	1.27	1.415
Q9CPU4	Microsomal glutathione S-transferase 3	Mgst3	1.827	0.000361566	Up	1.239	1.457	1.388	0.725	0.781	0.729
D0QMC3	Myeloid cell nuclear differentiation antigen-like protein	Mndal	0.712	0.000222156	Down	0.955	0.954	0.892	1.33	1.331	1.274
Q91VN4	MICOS complex subunit Mic25	Chchd6	1.364	0.003670902	Up	1.219	1.244	1.251	0.986	0.827	0.909
Q3TEW6	Myelin protein zero-like protein 1	Mpzl1	1.616	0.002453046	Up	1.053	0.963	1.194	0.635	0.705	0.646
P15532	Nucleoside diphosphate kinase A	Nme1	0.724	0.001393146	Down	0.8	0.896	0.791	1.133	1.134	1.17
Q6P5H2	Nestin	Nes	0.659	2.29748E-06	Down	0.773	0.765	0.751	1.156	1.148	1.172
A2AMM0	Caveolae-associated protein 4	Cavin4	1.768	2.59844E-06	Up	1.265	1.26	1.297	0.705	0.724	0.733
Q9CXS4	Centromere protein V	Cenpv	0.632	1.18115E-05	Down	0.899	0.936	0.924	1.464	1.482	1.421
Q8R1M2	Histone H2A.J	H2aj	0.131	0.000124495	Down	0.39	0.346	0.246	2.609	2.465	2.44
Q9JM52	Misshapen-like kinase 1	Mink1	0.663	0.008834681	Down	0.744	0.928	0.901	1.419	1.188	1.272
Q9JJW5	Myozenin-2	Myoz2	1.406	6.98806E-05	Up	1.292	1.306	1.308	0.896	0.924	0.959

P32020	Non-specific lipid-transfer protein	Scp2	1.376	0.000185583	Up	1.199	1.214	1.132	0.845	0.876	0.856
O35216	Histone H3-like centromeric protein A	Cenpa	0.165	0.000672571	Down	0.441	0.466	0.259	2.279	2.556	2.231
Q32NY4	Metal transporter	Cnnm3	0.629	0.002710452	Down	0.81	0.877	0.733	1.25	1.195	1.4
Q91YN0	Protein C12orf4	D6Wsu1	0.732	0.023595834	Down	0.887	0.908	0.803	1.155	1.038	1.356
Q7TNV0	Protein DEK	Dek	0.748	0.001078992	Down	0.903	0.849	0.811	1.115	1.139	1.174
Q2VPQ9	Chromatin modification-related protein MEAF6	Meaf6	0.59	8.32757E-05	Down	0.789	0.75	0.788	1.255	1.381	1.307
Q99MU3	Double-stranded RNA-specific adenosine deaminase	Adar	0.7	0.00320089	Down	0.962	0.828	0.815	1.232	1.288	1.2
P49710	Hematopoietic lineage cell-specific protein	Hcls1	0.611	3.73706E-05	Down	0.769	0.793	0.742	1.245	1.23	1.295
Q61646	Haptoglobin	Hp	2.002	6.60098E-06	Up	1.596	1.558	1.554	0.754	0.81	0.788
Q9CWS4	Integrator complex subunit 11	Ints11	0.743	0.04390953	Down	0.928	0.803	0.877	1.307	1.232	0.971
O35343	Importin subunit alpha-3	Kpna4	0.733	0.016333607	Down	0.885	0.704	0.863	1.116	1.176	1.054
Q60766	Immunity-related GTPase family M	Irgm1	0.761	0.000481023	Down	0.97	0.985	0.908	1.255	1.272	1.236
Q61771	Kinesin-like protein	Kif3b	0.732	0.019372492	Down	0.823	0.689	0.898	1.092	1.16	1.042
Q61827	Transcription factor	Mafk	0.676	0.001233607	Down	0.948	0.907	0.972	1.369	1.509	1.302
Q9R0Q4	Mortality factor 4-like protein 2	Morf4l2	0.608	0.007643396	Down	0.807	0.875	0.719	1.111	1.411	1.425
Q6WKZ7	Nostrin	Nostrin	0.623	0.001229649	Down	0.722	0.836	0.872	1.332	1.291	1.28
Q9JJY3	Sphingomyelin phosphodiesterase 3	Smpd3	0.065	0.005873477	Down	0.251	0.235	0.046	2.857	2.619	2.716
Q9QXE2	DNA polymerase	Poll	0.502	1.65924E-05	Down	0.722	0.666	0.716	1.367	1.423	1.405
P54869	Hydroxymethylglutaryl-CoA synthase, mitochondrial	Hmgcs2	4.049	4.53084E-05	Up	1.393	1.607	1.746	0.4	0.366	0.406
Q9Z2B9	Ribosomal protein S6 kinase alpha-4	Rps6ka4	0.717	0.000421179	Down	0.846	0.9	0.818	1.218	1.192	1.164
Q9WUV0	Origin recognition complex subunit 5	Orc5	0.644	0.003451109	Down	0.717	0.783	0.907	1.271	1.194	1.272
Q9EP73	Programmed cell death 1 ligand 1	Cd274	0.673	0.00047363	Down	1.007	0.993	0.903	1.404	1.483	1.425
Q8VE95	UPF0598 protein C8orf82 homolog	--	1.301	0.000226469	Up	1.231	1.27	1.198	0.936	0.971	0.936
P36552	Oxygen-dependent coproporphyrinogen-III oxidase, mitochondrial	Cpox	1.342	0.003098163	Up	1.157	1.109	1.262	0.888	0.833	0.908
Q64112	Interferon-induced protein with tetratricopeptide repeats	Ifit2	0.38	6.09729E-05	Down	0.693	0.574	0.625	1.678	1.633	1.671
P51125	Calpastatin	Cast	0.725	0.000553879	Down	0.937	0.879	0.896	1.294	1.186	1.26
P21812	Mast cell protease 4	Mcpt4	0.268	2.84462E-05	Down	0.587	0.481	0.516	1.953	1.908	2.045
P19258	Protein Mpv17	Mpv17	1.47	0.00924037	Up	1.277	1.401	1.193	0.832	0.999	0.802
Q9QVP4	Myosin regulatory light chain 2, atrial isoform	Myl7	1.729	6.28623E-05	Up	1.352	1.259	1.305	0.791	0.739	0.735
Q9CQ91	NADH dehydrogenase [ubiquinone] 1 alpha subcomplex subunit 3	Ndufa3	1.537	0.001478983	Up	1.219	1.204	1.371	0.766	0.835	0.867
Q61937	Nucleophosmin	Npm1	0.763	0.000606762	Down	0.852	0.845	0.898	1.164	1.148	1.09
O35143	ATPase inhibitor, mitochondrial	ATP5IF1	1.393	0.001079814	Up	1.172	1.272	1.221	0.88	0.923	0.828
Q9QZQ8	Core histone macro-	Macroh2	0.277	3.67462E-05	Down	0.59	0.548	0.475	1.965	1.946	1.906
Q9D168	Integrator complex subunit 12	Ints12	0.761	0.043993021	Down	0.823	1.007	0.741	1.198	1.063	1.117
P09541	Myosin light chain 4	Myl4	1.645	0.000141282	Up	1.273	1.248	1.391	0.806	0.778	0.794
E9Q634	Unconventional myosin-	Myo1e	0.584	0.000357465	Down	0.816	0.695	0.761	1.329	1.279	1.283
Q9DCN1	Peroxisomal NADH pyrophosphatase	Nudt12	1.315	0.00242153	Up	1.131	1.263	1.158	0.884	0.94	0.877
P03888	NADH-ubiquinone oxidoreductase chain 1	Mtnd1	1.301	0.001867755	Up	1.202	1.243	1.16	0.894	0.98	0.897
Q5U458	DnaJ homolog subfamily C member 11	Dnajc11	1.307	6.716E-05	Up	1.226	1.202	1.223	0.908	0.932	0.954
P31001	Desmin	Des	1.315	3.2532E-05	Up	1.184	1.165	1.208	0.888	0.904	0.913
O08528	Hexokinase-2	Hk2	1.328	0.000620661	Up	1.207	1.175	1.294	0.93	0.92	0.918
P40936	Indolethylamine N-methyltransferase	Inmt	0.436	3.93538E-06	Down	0.686	0.707	0.693	1.595	1.655	1.534
Q3TN34	MICAL-like protein 2	Micall2	0.621	0.006131632	Down	0.925	0.863	0.73	1.509	1.264	1.282
Q9D6Z1	Nucleolar protein 56	Nop56	0.577	0.000147334	Down	0.825	0.863	0.762	1.375	1.44	1.428
Q61324	Aryl hydrocarbon receptor nuclear	Arnt2	1.437	0.015251808	Up	1.363	1.136	1.254	0.75	0.949	0.912
O55143	Sarcoplasmic/endoplasmic reticulum calcium	Atp2a2	1.635	1.90968E-05	Up	1.444	1.402	1.493	0.891	0.867	0.896

Q8K363	ATP-dependent RNA helicase DDX18	Ddx18	0.71	0.000105371	Down	0.928	0.956	0.9	1.344	1.286	1.292
Q9CXY6	Interleukin enhancer-binding factor 2	Ilf2	0.761	0.002105059	Down	0.822	0.889	0.861	1.183	1.133	1.063
A2ASA8	Inositol 1,4,5-trisphosphate receptor-interacting protein-like 1	Itpripl1	1.331	0.001373168	Up	1.086	1.172	1.113	0.8	0.881	0.852
Q6ZQ58	La-related protein 1	Larp1	0.614	0.004489109	Down	0.868	0.789	0.842	1.441	1.478	1.153
Q6KCD5	Nipped-B-like protein	Nipbl	0.687	6.71597E-05	Down	0.874	0.856	0.89	1.289	1.224	1.299
Q8BGN5	NIPA-like protein 3	Nipal3	0.697	0.000658471	Down	0.799	0.825	0.876	1.245	1.202	1.138
Q9DCV4	Regulator of microtubule dynamics	Rmdn1	1.531	7.12295E-05	Up	1.309	1.347	1.343	0.849	0.912	0.851
Q9CQA3	Succinate dehydrogenase [ubiquinone] iron-sulfur	Sdhb	1.352	8.53636E-05	Up	1.252	1.205	1.232	0.919	0.883	0.927
Q62141	Paired amphipathic helix protein Sin3b	Sin3b	0.609	0.031412363	Down	0.983	0.738	0.685	1.055	1.41	1.484
Q8QZY9	Splicing factor 3B	Sf3b4	0.675	0.036082222	Down	0.706	0.789	0.928	0.98	1.351	1.256
Q8BHL3	TBC1 domain family member 10B	Tbc1d10	0.694	0.015969275	Down	1.018	0.775	0.793	1.217	1.19	1.318
Q9ERP3	Tripartite motif-containing protein 54	Trim54	1.514	0.006193812	Up	1.215	1.555	1.403	0.899	0.882	0.975
A2AR02	Peptidyl-prolyl cis-trans isomerase G	Ppig	0.751	0.036310516	Down	0.851	0.792	0.758	1.242	1.018	0.936
Q8BWF0	Succinate-semialdehyde dehydrogenase, mitochondrial	Aldh5a1	1.34	5.73255E-06	Up	1.258	1.236	1.249	0.942	0.918	0.934
P21981	Protein-glutamine gamma-	Tgm2	1.306	2.56987E-06	Up	1.029	1.02	1.006	0.777	0.78	0.782
Q8K093	Thyrotropin-releasing hormone-degrading ectoenzyme	Trhde	0.447	0.001308387	Down	0.612	0.571	0.445	1.189	1.287	1.166
Q9Z126	Platelet factor 4	Pf4	0.226	0.000832029	Down	0.526	0.558	0.337	1.893	2.235	2.165
Q8JZX4	Splicing factor 45	Rbm17	0.717	0.003010222	Down	0.797	0.902	0.813	1.184	1.229	1.092
P62317	Small nuclear ribonucleoprotein Sm	Snrpd2	0.753	0.000528197	Down	0.912	0.852	0.833	1.152	1.162	1.136
Q99PP6	Tripartite motif-containing protein 34A	Trim34a	0.656	0.003611354	Down	0.947	0.936	0.774	1.312	1.415	1.325
Q91ZJ5	UTP--glucose-1-phosphate	Ugp2	0.734	0.000447587	Down	0.846	0.844	0.821	1.199	1.092	1.128
Q9R1C7	Pre-mRNA-processing factor 40 homolog A	Prpf40a	0.674	0.000842321	Down	0.891	0.781	0.799	1.235	1.248	1.182
Q91ZW3	SWI/SNF-related matrix-associated actin-dependent regulator of chromatin subfamily A	Smarca5	0.642	2.58855E-05	Down	0.87	0.82	0.819	1.301	1.296	1.313
P84104	Serine/arginine-rich splicing factor 3	Srsf3	0.741	0.000505565	Down	0.844	0.842	0.839	1.202	1.112	1.094
Q9Z2I8	Succinate--CoA ligase [GDP-forming] subunit beta, mitochondrial	Suclg2	1.434	3.732E-05	Up	1.315	1.258	1.29	0.921	0.887	0.886
Q80X71	Transmembrane protein 106B	Tmem10	1.408	0.001429199	Up	1.106	1.127	1.226	0.832	0.853	0.772
P97371	Proteasome activator complex subunit 1	Psme1	0.679	3.48217E-06	Down	0.852	0.835	0.828	1.242	1.218	1.242
P61255	60S ribosomal protein	Rpl26	0.551	0.000154088	Down	0.79	0.782	0.697	1.409	1.369	1.338
O55142	60S ribosomal protein L35a	Rpl35a	0.552	1.94011E-05	Down	0.79	0.728	0.772	1.39	1.392	1.363
Q9CR57	60S ribosomal protein	Rpl14	0.56	0.000361084	Down	0.783	0.888	0.753	1.4	1.461	1.466
P62754	40S ribosomal protein	Rps6	0.516	5.28651E-06	Down	0.748	0.732	0.702	1.431	1.396	1.398
P62309	Small nuclear ribonucleoprotein G	Snrpg	0.707	0.004817123	Down	0.774	0.789	0.896	1.19	1.068	1.219
Q812A2	SLIT-ROBO Rho GTPase-activating	Srgap3	0.663	0.000915022	Down	0.81	0.911	0.783	1.238	1.28	1.259
Q8BTI8	Serine/arginine repetitive matrix protein	Srrm2	0.628	0.000708702	Down	0.835	0.842	0.95	1.449	1.408	1.324
Q70IV5	Synemin	Synm	0.739	0.001897865	Down	0.947	0.886	0.873	1.253	1.269	1.142
P97500	Myelin transcription factor 1-like protein	Myt1l	0.689	0.002737287	Down	0.793	0.753	0.853	1.26	1.133	1.09
P70255	Nuclear factor 1 C-type	Nfic	0.747	0.007828046	Down	0.981	0.896	0.863	1.322	1.212	1.132
Q9DB77	Cytochrome b-c1 complex subunit 2,	Uqcrc2	1.306	6.61687E-06	Up	1.211	1.224	1.229	0.924	0.948	0.933
P62918	60S ribosomal protein	Rpl8	0.531	6.0019E-05	Down	0.723	0.764	0.791	1.415	1.498	1.379
P47911	60S ribosomal protein	Rpl6	0.522	0.000125845	Down	0.719	0.837	0.76	1.482	1.474	1.478
Q9EPB5	Serine hydrolase-like protein	Serhl	1.541	0.000344247	Up	1.256	1.175	1.33	0.818	0.829	0.794
Q91YU8	Suppressor of SWI4 1 homolog	Ppan	0.375	0.002043491	Down	0.764	0.683	0.48	1.73	1.718	1.692

Q9EPT5	Solute carrier organic anion transporter family member 2A1	Slco2a1	2.484	7.89959E-05	Up	0.965	1.099	1.11	0.409	0.416	0.453
Q60864	Stress-induced-phosphoprotein 1	Stip1	0.725	3.08697E-06	Down	0.871	0.857	0.855	1.202	1.176	1.186
Q8VDS8	Syntaxin-18	Stx18	0.759	0.006875313	Down	0.791	0.932	0.854	1.152	1.074	1.169
Q9QZH3	Peptidyl-prolyl cis-trans isomerase E	Ppie	0.742	0.023897547	Down	0.767	0.899	0.876	1.056	1.302	1.07
Q64012	RNA-binding protein	Raly	0.595	2.47656E-05	Down	0.732	0.783	0.777	1.291	1.303	1.26
Q9D706	RNA polymerase II-associated protein 3	Rpap3	0.754	0.011274257	Down	0.829	0.834	0.817	1.16	1.164	0.964
P62270	40S ribosomal protein	Rps18	0.746	0.000178534	Down	0.855	0.827	0.88	1.134	1.128	1.174
A2AQ19	RNA polymerase-associated protein RTF1 homolog	Rtf1	0.712	0.009379208	Down	0.914	0.785	0.738	1.088	1.111	1.222
E9QAT4	Protein transport protein Sec16A	Sec16a	1.761	0.003448366	Up	1.125	1.138	1.128	0.534	0.661	0.731
Q78RX3	Small integral membrane protein 12	Smim12	1.331	3.18733E-05	Up	1.178	1.226	1.215	0.901	0.899	0.918
P49962	Signal recognition particle 9 kDa protein	Srp9	0.681	0.000592795	Down	0.866	0.827	0.88	1.326	1.27	1.18
P32921	Tryptophan--tRNA ligase, cytoplasmic	Wars1	0.696	2.22542E-06	Down	0.897	0.903	0.884	1.294	1.293	1.269
P21958	Antigen peptide transporter 1	Tap1	0.589	0.000125059	Down	0.912	0.835	0.82	1.432	1.496	1.429
Q8BRH0	Protein O-mannosyl-transferase TMTC3	Tmtc3	1.409	0.000517771	Up	1.21	1.235	1.263	0.902	0.823	0.906
Q60715	Prolyl 4-hydroxylase subunit alpha-1	P4ha1	0.726	0.000361204	Down	0.911	0.832	0.849	1.208	1.18	1.18
Q8CHY6	Transcriptional repressor p66 alpha	Gatad2a	0.664	0.000901648	Down	0.907	0.825	0.815	1.202	1.337	1.297
Q9QXV0	ProSAAS	Pcsk1n	1.462	0.007743641	Up	1.052	0.89	1.134	0.719	0.667	0.718
P47963	60S ribosomal protein	Rpl13	0.457	2.63519E-05	Down	0.723	0.711	0.66	1.585	1.53	1.466
Q8VCQ6	Phosphatidylcholine:ceramide cholinephosphotransferase 1	Sgms1	1.471	0.006742279	Up	1.024	1.196	1.051	0.703	0.692	0.829
A6H5X4	PHD finger protein 11	Phf11	0.375	0.001105395	Down	0.754	0.555	0.582	1.825	1.762	1.461
P35980	60S ribosomal protein	Rpl18	0.553	5.68866E-05	Down	0.802	0.812	0.757	1.5	1.402	1.384
P27659	60S ribosomal protein	Rpl3	0.562	0.000311687	Down	0.799	0.817	0.698	1.354	1.39	1.376
Q9D1R9	60S ribosomal protein	Rpl34	0.444	4.77041E-06	Down	0.673	0.696	0.643	1.496	1.535	1.502
P62855	40S ribosomal protein	Rps26	0.511	0.005368234	Down	0.654	0.912	0.619	1.455	1.482	1.34
Q60710	Deoxynucleoside triphosphate triphosphohydrolase SAMHD1	Samhd1	0.697	6.79167E-05	Down	0.862	0.82	0.818	1.168	1.218	1.202
Q62203	Splicing factor 3A	Sf3a2	0.742	0.006444385	Down	0.952	0.905	0.855	1.334	1.18	1.14
Q3UV70	[Pyruvate dehydrogenase [acetyl-transferring]]-phosphatase 1,	Pdp1	1.398	0.000475158	Up	1.158	1.271	1.184	0.842	0.887	0.855
Q3TVI8	Pre-B-cell leukemia transcription factor-interacting protein 1	Pbxip1	1.535	0.001553519	Up	1.298	1.229	1.299	0.763	0.915	0.815
Q9JHK5	Pleckstrin	Plek	0.246	0.000116283	Down	0.551	0.476	0.4	1.986	1.943	1.869
O35955	Proteasome subunit beta type-10	Psmb10	0.658	0.000157598	Down	0.83	0.885	0.802	1.288	1.286	1.252
P47915	60S ribosomal protein	Rpl29	0.397	2.89998E-06	Down	0.672	0.707	0.656	1.741	1.682	1.697
P25444	40S ribosomal protein	Rps2	0.728	0.000251973	Down	0.872	0.873	0.829	1.224	1.154	1.156
Q5M8N0	CB1 cannabinoid receptor-interacting	Cnrip1	1.532	0.00106414	Up	1.293	1.168	1.312	0.877	0.803	0.782
P27661	Histone H2AX	H2ax	0.143	4.19296E-05	Down	0.363	0.366	0.271	2.403	2.245	2.325
Q8K201	Keratinocyte-associated transmembrane protein 2	Kct2	1.422	0.000969407	Up	1.088	1.148	1.048	0.723	0.794	0.792
Q7TNG8	Probable D-lactate dehydrogenase, mitochondrial	Ldhd	1.378	0.00858082	Up	1.205	1.174	1.293	0.919	0.786	0.96
P19137	Laminin subunit alpha-1	Lama1	0.681	0.015086205	Down	0.681	0.684	0.895	1.037	1.13	1.152
Q8K3W2	Leucine-rich repeat-containing protein 10	Lrrc10	1.402	0.011931467	Up	1.15	1.228	1.002	0.857	0.73	0.824
Q9CRB9	MICOS complex subunit Mic19	Chchd3	1.802	1.24656E-06	Up	1.411	1.436	1.456	0.796	0.784	0.808
Q8BLF1	Neutral cholesterol ester hydrolase 1	Nceh1	1.391	0.000271719	Up	1.308	1.249	1.35	0.915	0.929	0.964
Q62425	Cytochrome c oxidase subunit NDUFA4	Ndufa4	1.613	3.17672E-05	Up	1.338	1.349	1.358	0.873	0.81	0.824
Q3UIZ8	Myosin light chain	Mylk3	1.329	0.000811981	Up	1.125	1.165	1.219	0.916	0.854	0.87
Q9D4J7	PHD finger protein 6	Phf6	0.723	0.000145986	Down	0.837	0.812	0.855	1.115	1.181	1.165

O35691	Pinin	Pnn	0.743	0.000361872	Down	0.869	0.92	0.84	1.183	1.182	1.175
P48774	Glutathione S-transferase Mu 5	Gstm5	1.364	0.023190427	Up	1.095	0.985	1.167	0.687	0.876	0.817
Q8CCK0	Core histone macro-	Macroh2	0.388	1.35318E-06	Down	0.673	0.666	0.66	1.776	1.716	1.658
Q9D8C4	Interferon-induced 35 kDa protein homolog	Ifi35	0.735	0.005744805	Down	0.918	0.88	0.773	1.168	1.215	1.114
Q60722	Transcription factor 4	Tcf4	0.69	0.000418927	Down	0.874	0.911	0.954	1.269	1.327	1.376
Q99LC3	NADH dehydrogenase [ubiquinone] 1 alpha subcomplex subunit 10, mitochondrial	Ndufa10	1.379	2.40117E-06	Up	1.231	1.227	1.227	0.901	0.894	0.877
Q8R5F7	Interferon-induced helicase C domain-containing protein 1	Ifih1	0.696	1.2227E-05	Down	0.858	0.875	0.872	1.22	1.273	1.25
Q8BV49	Pyrin and HIN domain-containing protein 1	Pyhin1	0.762	0.002752664	Down	0.937	0.874	0.848	1.123	1.135	1.23
Q8R1F0	Leydig cell tumor 10 kDa protein homolog	D8Ertd7	0.768	0.03308415	Down	1.083	0.937	0.833	1.33	1.189	1.197
Q9CXJ4	Mitochondrial potassium channel ATP-binding subunit	Abcb8	2.548	4.68527E-06	Up	1.578	1.614	1.65	0.617	0.618	0.665
Q62234	Myomesin-1	Myom1	1.53	9.81445E-06	Up	1.412	1.379	1.452	0.925	0.929	0.919
Q5SZT7	NKAP-like protein	Nkapl	0.572	0.000300905	Down	0.752	0.857	0.759	1.342	1.441	1.355
Q99P30	Peroxisomal coenzyme A diphosphatase	Nudt7	1.362	0.000143617	Up	1.242	1.199	1.239	0.926	0.869	0.906
Q8R1N4	NudC domain-containing protein 3	Nudcd3	0.757	0.001010096	Down	0.943	0.877	0.846	1.168	1.188	1.165
O70571	[Pyruvate dehydrogenase (acetyl-transferring)] kinase	Pdk4	1.535	0.00125106	Up	1.216	1.345	1.259	0.76	0.878	0.851
Q3TCJ8	Coiled-coil domain-containing protein 69	Ccdc69	0.741	0.000955124	Down	0.889	0.831	0.84	1.09	1.185	1.181
P12787	Cytochrome c oxidase subunit 5A,	Cox5a	1.304	8.34742E-05	Up	1.201	1.26	1.239	0.933	0.945	0.96
P84244	Histone H3.3	H3-3a	0.108	0.000169163	Down	0.247	0.368	0.214	2.624	2.64	2.39
Q8R0N6	Hydroxyacid-oxoacid transhydrogenase, mitochondrial	Adhfe1	1.438	0.004135925	Up	1.095	1.303	1.296	0.84	0.894	0.835
P11499	Heat shock protein HSP 90-beta	Hsp90ab	0.721	8.70662E-07	Down	0.85	0.834	0.844	1.176	1.164	1.166
Q9D0E1	Heterogeneous nuclear ribonucleoprotein M	Hnrnpm	0.719	2.30259E-05	Down	0.867	0.84	0.833	1.175	1.196	1.163
Q61581	Insulin-like growth factor-binding protein 7	Igfbp7	0.595	0.003097844	Down	0.863	0.754	0.652	1.258	1.31	1.246
Q91W39	Nuclear receptor coactivator 5	Ncoa5	0.742	0.000229794	Down	0.832	0.868	0.899	1.176	1.149	1.177
Q9Z1P6	NADH dehydrogenase [ubiquinone] 1 alpha subcomplex subunit 7	Ndufa7	1.362	9.74676E-06	Up	1.267	1.239	1.227	0.924	0.908	0.909
Q8VHR5	Transcriptional repressor p66-beta	Gatad2b	0.575	0.000631818	Down	0.792	0.694	0.801	1.372	1.237	1.365
Q8BTI9	Phosphatidylinositol 4,5-bisphosphate 3-kinase catalytic subunit beta isoform	Pik3cb	0.734	0.009515097	Down	0.795	0.908	0.907	1.081	1.194	1.281
Q9D937	Uncharacterized protein C11orf98 homolog	--	0.582	5.24901E-05	Down	0.824	0.774	0.856	1.415	1.395	1.408
B2RSH2	Guanine nucleotide-binding protein G(i)	Gnai1	1.502	0.002330688	Up	1.263	1.197	1.124	0.855	0.724	0.807
P02104	Hemoglobin subunit epsilon-Y2	Hbb-y	0.696	0.006300748	Down	0.73	0.704	0.848	1.171	1.087	1.021
Q9DC33	High mobility group protein 20A	Hmg20a	0.715	0.039598079	Down	0.909	0.901	0.793	1.217	1.005	1.417
Q64339	Ubiquitin-like protein ISG15	Isg15	0.49	6.70313E-05	Down	0.686	0.775	0.69	1.494	1.432	1.465
Q6P8J7	Creatine kinase S-type, mitochondrial	Ckmt2	1.927	9.24412E-06	Up	1.426	1.425	1.469	0.72	0.748	0.774
P32443	Homeobox protein	Meox2	0.638	0.000549808	Down	0.854	0.822	0.843	1.238	1.428	1.281
P42925	Peroxisomal membrane protein 2	Pxmp2	1.368	0.00331608	Up	1.279	1.237	1.266	1.013	0.889	0.862
P70429	Ena/VASP-like protein	Evl	0.688	0.001038417	Down	0.804	0.868	0.792	1.117	1.22	1.246
Q9CR98	Protein FAM136A	Fam136a	1.37	0.020017791	Up	1.352	1.2	1.166	0.962	0.78	0.972
P10853	Histone H2B type 1-	H2bc7	0.148	3.35638E-05	Down	0.4	0.34	0.292	2.274	2.433	2.27
Q9DB91	Mediator of RNA polymerase II transcription subunit 29	Med29	0.697	0.035912617	Down	0.999	0.785	0.861	1.11	1.5	1.187

Q921V5	Alpha-1,6-mannosyl-glycoprotein 2-beta-N-acetylglucosaminyltransferase	Mgat2	1.432	0.041321928	Up	1.215	1.296	1.084	0.747	1.035	0.728
O08692	Neutrophilic granule	Ngp	1.354	0.029391473	Up	1.43	1.053	1.243	0.909	0.935	0.908
P49717	DNA replication licensing factor MCM4	Mcm4	0.545	0.001787441	Down	0.767	0.657	0.624	1.383	1.151	1.223
P03893	NADH-ubiquinone oxidoreductase chain 2	Mtnd2	1.616	0.002819059	Up	1.197	1.481	1.327	0.867	0.763	0.848
Q3UHB1	5'-nucleotidase domain-containing protein 3	Nt5dc3	1.417	0.007366	Up	1.182	1.29	1.291	0.795	0.99	0.87
Q99NH2	Partitioning defective 3 homolog	Pard3	1.343	0.005206592	Up	1.303	1.189	1.253	1.018	0.885	0.886
Q9CW46	Ribonucleoprotein PTB-binding 1	Raver1	0.768	0.004933732	Down	0.876	0.839	0.785	1.06	1.16	1.036
Q60843	Krueppel-like factor 2	Klf2	0.472	0.012559444	Down	0.533	0.912	0.612	1.673	1.286	1.395
Q9Z2E2	Methyl-CpG-binding domain protein 1	Mbd1	0.587	0.005707022	Down	0.97	0.82	0.71	1.524	1.419	1.314
Q9DCZ4	MICOS complex subunit Mic26	Apoo	1.392	0.006083185	Up	1.348	1.15	1.336	0.856	0.946	0.953
Q8R1S4	Protein MTSS 1	Mtss1	0.709	0.000220333	Down	0.86	0.881	0.806	1.195	1.21	1.188
O08600	Endonuclease G, mitochondrial	Endog	1.301	0.002930432	Up	1.16	1.198	1.234	0.94	0.966	0.856
P58281	Dynamin-like 120 kDa protein, mitochondrial	Opa1	1.583	1.01272E-05	Up	1.357	1.293	1.347	0.833	0.853	0.839
P63250	G protein-activated inward rectifier potassium channel 1	Kcnj3	1.419	0.033667703	Up	1.07	1.106	1.437	0.763	0.921	0.862
Q8R4U7	Leucine zipper protein 1	Luzp1	0.75	0.003307001	Down	0.858	0.958	0.906	1.214	1.275	1.138
Q3UN70	Myelin regulatory factor-like protein	Myrfl	0.732	0.005255661	Down	1.095	0.993	0.956	1.495	1.313	1.35
Q7TMF3	NADH dehydrogenase [ubiquinone] 1 alpha subcomplex subunit 12	Ndufa12	1.311	3.87901E-05	Up	1.172	1.182	1.186	0.924	0.887	0.889
P03903	NADH-ubiquinone oxidoreductase chain 4L	Mtnd4l	1.348	0.001875892	Up	1.189	1.239	1.292	0.899	0.981	0.88
Q8R2Y8	Peptidyl-tRNA hydrolase 2,	Ptrh2	1.491	0.000884623	Up	1.253	1.199	1.373	0.882	0.861	0.823
Q0II04	Nebulette	Nebl	1.567	3.41461E-05	Up	1.31	1.38	1.385	0.849	0.887	0.865
Q91YE7	RNA-binding protein 5	Rbm5	0.716	0.00438439	Down	0.823	0.935	0.772	1.164	1.211	1.158
P53026	60S ribosomal protein L10a	Rpl10a	0.506	3.2446E-06	Down	0.75	0.732	0.712	1.428	1.434	1.478
P62717	60S ribosomal protein L18a	Rpl18a	0.619	0.00016344	Down	0.872	0.871	0.787	1.36	1.384	1.344
Q8BP67	60S ribosomal protein	Rpl24	0.538	2.04161E-05	Down	0.766	0.786	0.74	1.436	1.458	1.364
Q6ZWV7	60S ribosomal protein	Rpl35	0.472	2.35032E-05	Down	0.694	0.712	0.638	1.43	1.456	1.444
P14869	60S acidic ribosomal protein P0	Rplp0	0.674	0.000153373	Down	0.83	0.845	0.784	1.229	1.176	1.244
Q9D8E6	60S ribosomal protein	Rpl4	0.521	1.33199E-05	Down	0.798	0.759	0.735	1.461	1.451	1.491
P83882	60S ribosomal protein L36a	Rpl36a	0.523	5.62671E-05	Down	0.789	0.741	0.698	1.437	1.425	1.401
Q91VM5	RNA binding motif protein, X-linked-like-1	Rbmxl1	0.759	0.000215075	Down	0.848	0.872	0.881	1.107	1.139	1.179
Q8R127	Saccharopine dehydrogenase-like oxidoreductase	Sccpdh	1.452	0.000253426	Up	1.257	1.163	1.262	0.87	0.832	0.833
Q60598	Src substrate cortactin	Cttn	0.694	7.43128E-05	Down	0.869	0.847	0.809	1.227	1.207	1.203
Q8BVK9	Sp110 nuclear body	Sp110	0.501	0.001951792	Down	0.889	0.646	0.698	1.449	1.494	1.517
P52840	Sulfotransferase 1A1	Sult1a1	1.513	0.000165388	Up	1.05	1.138	1.061	0.732	0.693	0.723
Q6P3A8	2-oxoisovalerate dehydrogenase subunit beta, mitochondrial	Bckdhb	1.419	5.2319E-05	Up	1.185	1.221	1.249	0.869	0.868	0.839
Q9CUX1	52 kDa repressor of the inhibitor of the protein kinase	Thap12	0.724	0.023514537	Down	1.004	0.772	0.805	1.288	1.155	1.12
Q8CG03	cGMP-specific 3',5'-cyclic phosphodiesterase	Pde5a	0.559	0.000372353	Down	0.857	0.822	0.742	1.364	1.513	1.452
Q68FH0	Plakophilin-4	Pkp4	1.544	6.48934E-06	Up	1.173	1.19	1.212	0.782	0.756	0.777
Q99J62	Replication factor C subunit 4	Rfc4	0.703	0.018872331	Down	0.822	0.952	0.753	1.124	1.118	1.351
Q9CXW4	60S ribosomal protein	Rpl11	0.733	0.001858441	Down	0.84	0.831	0.748	1.089	1.067	1.146
Q9CPR4	60S ribosomal protein	Rpl17	0.641	0.000538676	Down	0.783	0.804	0.808	1.172	1.212	1.35
E9Q555	E3 ubiquitin-protein ligase RNF213	Rnf213	0.708	7.01095E-05	Down	0.875	0.84	0.84	1.228	1.168	1.213
Q8BGH2	Sorting and assembly machinery component 50 homolog	Samm50	1.481	2.10545E-05	Up	1.294	1.256	1.332	0.871	0.876	0.874
P19324	Serpin H1	Serpinh1	0.702	7.22515E-05	Down	0.847	0.828	0.792	1.165	1.189	1.162

Q9CSN1	SNW domain-containing protein 1	Snw1	0.571	4.22299E-05	Down	0.814	0.832	0.758	1.398	1.424	1.39
Q925B0	PRKC apoptosis WT1 regulator protein	Pawr	0.548	0.000379268	Down	0.826	0.839	0.836	1.67	1.386	1.508
Q3TC46	Protein PAT1 homolog 1	Patl1	1.508	0.010545999	Up	1.441	1.119	1.253	0.78	0.819	0.93
Q9DBD5	Proline-, glutamic acid- and leucine-rich protein	Pelp1	0.759	0.006577527	Down	0.922	0.771	0.86	1.119	1.146	1.097
Q80SY5	Pre-mRNA-splicing factor 38B	Prpf38b	0.663	0.001342551	Down	0.901	0.759	0.839	1.273	1.22	1.276
O09167	60S ribosomal protein	Rpl21	0.537	3.63589E-06	Down	0.743	0.757	0.734	1.416	1.396	1.347
P62984	Ubiquitin-60S ribosomal protein L40	Uba52	0.508	0.000191276	Down	0.788	0.677	0.757	1.52	1.398	1.454
P62852	40S ribosomal protein	Rps25	0.624	5.33126E-05	Down	0.794	0.826	0.764	1.3	1.276	1.244
O70622	Reticulon-2	Rtn2	1.361	0.003022556	Up	1.408	1.281	1.2	0.938	0.946	0.973
E9Q401	Ryanodine receptor 2	Ryr2	1.669	2.24518E-06	Up	1.436	1.428	1.434	0.837	0.871	0.867
Q9D7S7	60S ribosomal protein L22-like 1	Rpl22l1	0.614	0.008283574	Down	0.583	0.81	0.655	1.189	1.066	1.082
P41105	60S ribosomal protein	Rpl28	0.565	6.2655E-05	Down	0.784	0.784	0.728	1.376	1.298	1.39
P99027	60S acidic ribosomal protein P2	Rplp2	0.585	9.4623E-06	Down	0.746	0.765	0.782	1.273	1.324	1.326
Q8K2B3	Succinate dehydrogenase [ubiquinone]	Sdha	1.335	2.00853E-06	Up	1.222	1.219	1.213	0.921	0.901	0.916
A2A5Z6	E3 ubiquitin-protein ligase SMURF2	Smurf2	1.447	0.013159707	Up	1.102	1.301	1.354	0.928	0.766	0.902
Q9QUM0	Integrin alpha-IIb	Itga2b	0.32	5.39152E-06	Down	0.589	0.583	0.588	1.826	1.948	1.726
Q9Z2E1	Methyl-CpG-binding domain protein 2	Mbd2	0.709	2.34168E-05	Down	0.873	0.847	0.867	1.229	1.234	1.187
Q811U4	Mitofusin-1	Mfn1	1.376	0.000408256	Up	1.331	1.217	1.264	0.899	0.932	0.94
Q78IK4	MICOS complex subunit Mic27	Apool	1.482	5.49106E-05	Up	1.325	1.323	1.306	0.876	0.865	0.927
Q9CPP6	NADH dehydrogenase [ubiquinone] 1 alpha subcomplex subunit 5	Ndufa5	1.315	0.000283838	Up	1.238	1.215	1.203	0.905	0.909	0.967
Q99LH1	Nucleolar GTP-binding protein 2	Gnl2	0.641	0.010324361	Down	0.803	1.045	0.757	1.361	1.372	1.329
Q8K2T8	RNA polymerase II-associated factor 1 homolog	Paf1	0.746	0.000250027	Down	0.823	0.858	0.819	1.08	1.152	1.12
Q4ZJM7	Otolin-1	Otol1	1.774	0.00115091	Up	1.409	1.355	1.414	0.829	0.682	0.844
Q8BHL4	Retinoic acid-induced protein 3	Gprc5a	4.776	3.99853E-05	Up	1.285	1.327	1.333	0.265	0.318	0.243
Q923G2	DNA-directed RNA polymerases I, II, and III subunit RPABC3	Polr2h	0.719	0.00677931	Down	0.876	1.035	0.85	1.227	1.315	1.299
Q4QQM4	Tumor protein p53-inducible protein 11	Trp53i11	0.709	0.004498823	Down	0.965	0.804	0.91	1.305	1.276	1.195
Q8BVZ1	Perilipin-5	Plin5	1.8	3.37006E-06	Up	1.477	1.425	1.445	0.807	0.821	0.787
Q8K1L5	E3 ubiquitin-protein ligase PPP1R11	Ppp1r11	0.759	0.019801882	Down	0.788	0.895	0.914	1.027	1.249	1.147
Q8R1I1	Cytochrome b-c1 complex subunit 9	Uqcr10	1.308	7.39476E-05	Up	1.208	1.185	1.235	0.937	0.931	0.905
P35979	60S ribosomal protein	Rpl12	0.673	0.000286723	Down	0.83	0.837	0.806	1.296	1.215	1.164
P62301	40S ribosomal protein	Rps13	0.596	7.56756E-06	Down	0.813	0.773	0.773	1.326	1.325	1.308
Q5SUC9	Protein SCO1 homolog, mitochondrial	Sco1	1.532	0.001032914	Up	1.38	1.443	1.239	0.891	0.911	0.849
Q9CR46	Spindle and kinetochore-associated	Ska2	1.454	0.002464385	Up	1.287	1.212	1.087	0.81	0.796	0.861
Q91WK1	SPRY domain-containing protein 4	Spryd4	1.38	0.005474719	Up	0.998	1.18	1.184	0.818	0.83	0.788
Q91Z83	Myosin-7	Myh7	1.897	9.96578E-05	Up	1.134	1.138	1.155	0.556	0.613	0.638
Q08481	Platelet endothelial cell adhesion molecule	Pecam1	1.387	9.90703E-05	Up	1.058	1.108	1.071	0.754	0.795	0.784
Q8VE62	Polyadenylate-binding protein-interacting	Paip1	1.457	0.00108057	Up	1.061	1.205	1.194	0.784	0.772	0.818
P61014	Cardiac phospholamban	Pln	1.469	2.70011E-05	Up	1.388	1.398	1.412	0.974	0.963	0.921
Q6P6M5	Peroxisomal membrane protein 11C	Pex11g	1.532	0.00070117	Up	1.336	1.312	1.29	0.917	0.788	0.866
P62849	40S ribosomal protein	Rps24	0.5	0.000605339	Down	0.724	0.595	0.743	1.377	1.4	1.345
Q5I012	Putative sodium-coupled neutral amino acid transporter 10	Slc38a10	1.381	0.037265359	Up	1.254	1.087	1.439	0.925	1.008	0.804
Q7TSZ8	Nucleus accumbens-associated protein 1	Nacc1	0.726	0.008481447	Down	0.872	0.996	0.876	1.333	1.307	1.138
P70414	Sodium/calcium exchanger 1	Slc8a1	1.552	6.39771E-05	Up	1.177	1.18	1.236	0.743	0.778	0.794

P52503	NADH dehydrogenase [ubiquinone] iron-sulfur protein 6, mitochondrial	Ndufs6	1.31	0.000325603	Up	1.24	1.227	1.186	0.898	0.93	0.96
P97372	Proteasome activator complex subunit 2	Psme2	0.666	0.000212142	Down	0.856	0.814	0.803	1.296	1.227	1.19
Q8C2Q3	RNA-binding protein 14	Rbm14	0.729	0.00218978	Down	0.848	0.898	0.809	1.128	1.247	1.128
P21440	Phosphatidylcholine translocator ABCB4	Abcb4	1.364	3.23348E-05	Up	1.409	1.378	1.435	1.035	1.014	1.047
P0DN34	NADH dehydrogenase [ubiquinone] 1 beta subcomplex subunit 1	Ndufb1	1.359	0.003241333	Up	1.157	1.293	1.146	0.934	0.863	0.85
Q6R891	Neurabin-2	Ppp1r9b	0.753	0.002896618	Down	0.829	0.82	0.915	1.167	1.163	1.076
Q9EQQ9	Protein O-GlcNAcase	Oga	0.722	0.000537955	Down	0.922	0.856	0.843	1.25	1.185	1.195
P06800	Receptor-type tyrosine-protein phosphatase C	Ptprc	0.58	0.032592685	Down	0.729	0.476	0.758	1.217	1.233	0.936
Q3TZ89	Protein transport protein Sec31B	Sec31b	1.54	3.76138E-05	Up	1.292	1.257	1.231	0.81	0.845	0.8
Q6NXH8	Methyltransferase-like protein 25	Mettl25	0.114	0.000742404	Down	0.399	0.303	0.177	2.603	2.646	2.483
Q8C854	Myelin expression factor	Myef2	0.746	0.000443754	Down	0.855	0.856	0.812	1.104	1.102	1.176
O55126	Protein NipSnap	Nipsnap2	1.407	8.7961E-05	Up	1.178	1.265	1.219	0.875	0.868	0.859
Q8K2V1	Serine/threonine-protein phosphatase 4 regulatory subunit 1	Ppp4r1	0.765	0.000638125	Down	0.76	0.743	0.804	0.978	1.029	1.01
Q9CPX8	Cytochrome b-c1 complex subunit 10	Uqcr11	1.534	0.005323786	Up	1.124	1.45	1.314	0.812	0.852	0.87
Q8VDD5	Myosin-9	Myh9	0.752	1.42236E-05	Down	0.865	0.862	0.839	1.147	1.14	1.125
Q9R0E2	Procollagen-lysine,2-oxoglutarate 5-dioxygenase 1	Plod1	0.735	3.45465E-05	Down	0.864	0.851	0.892	1.195	1.17	1.182
P04925	Major prion protein	Prnp	1.68	0.041230359	Up	0.971	1.452	1.416	0.606	0.898	0.781
O35435	Dihydroorotate dehydrogenase (quinone), mitochondrial	Dhodh	1.341	0.001001155	Up	1.285	1.175	1.214	0.911	0.88	0.949
Q9CQY2	RNA guanine-N7 methyltransferase activating subunit	Ramac	0.489	0.000579928	Down	0.785	0.736	0.623	1.421	1.434	1.527
Q9CT10	Ran-binding protein 3	Ranbp3	0.648	0.001439322	Down	0.916	0.758	0.862	1.313	1.305	1.298
P62751	60S ribosomal protein L23a	Rpl23a	0.464	0.00013603	Down	0.761	0.659	0.645	1.52	1.451	1.482
P47964	60S ribosomal protein	Rpl36	0.478	1.365E-05	Down	0.694	0.744	0.698	1.5	1.529	1.438
P47955	60S acidic ribosomal protein P1	Rplp1	0.637	0.000612677	Down	0.835	0.779	0.743	1.197	1.313	1.193
Q91VS7	Microsomal glutathione S-transferase 1	Mgst1	1.816	3.93603E-05	Up	1.268	1.382	1.364	0.734	0.72	0.756
Q9D517	1-acyl-sn-glycerol-3-phosphate acyltransferase gamma	Agpat3	1.742	0.000185699	Up	1.3	1.426	1.416	0.747	0.812	0.819
P43883	Perilipin-2	Plin2	1.619	0.001250134	Up	1.332	1.277	1.398	0.76	0.804	0.911
Q9CR16	Peptidyl-prolyl cis-trans isomerase D	Ppid	0.761	0.001294634	Down	0.866	0.909	0.825	1.181	1.13	1.106
P42669	Transcriptional activator protein Pur-alpha	Pura	0.75	3.26021E-06	Down	0.854	0.862	0.871	1.156	1.138	1.157
Q921W4	Quinone oxidoreductase-like	Cryzl1	0.654	0.001655585	Down	0.865	0.735	0.785	1.287	1.159	1.2
Q8BHS3	Pre-mRNA-splicing factor RBM22	Rbm22	0.719	0.000740379	Down	0.865	0.859	0.838	1.215	1.24	1.11
P62830	60S ribosomal protein	Rpl23	0.666	0.000928147	Down	0.814	0.863	0.799	1.334	1.215	1.166
P67984	60S ribosomal protein	Rpl22	0.679	0.001880488	Down	0.845	0.81	0.742	1.172	1.254	1.104
P62900	60S ribosomal protein	Rpl31	0.483	1.88816E-05	Down	0.723	0.743	0.674	1.491	1.49	1.446
Q64345	Interferon-induced protein with tetratricopeptide repeats	Ifit3	0.426	4.97602E-05	Down	0.686	0.716	0.617	1.548	1.619	1.574
O08573	Galectin-9	Lgals9	0.76	0.001596709	Down	0.895	0.792	0.848	1.124	1.114	1.096
P25206	DNA replication licensing factor MCM3	Mcm3	0.745	0.000606603	Down	0.827	0.88	0.817	1.172	1.1	1.115
P97310	DNA replication licensing factor MCM2	Mcm2	0.73	0.0004671	Down	0.879	0.822	0.808	1.111	1.169	1.158
Q99PG2	Opioid growth factor receptor	Ogfr	0.722	0.000753404	Down	0.826	0.859	0.856	1.249	1.12	1.151
Q6DID5	PWWP domain-containing DNA repair	Pwwp3a	0.742	0.004516436	Down	0.972	0.822	0.938	1.199	1.244	1.241
Q8VH51	RNA-binding protein 39	Rbm39	0.652	0.000420185	Down	0.846	0.783	0.823	1.215	1.336	1.209
Q9CZM2	60S ribosomal protein	Rpl15	0.522	0.000496386	Down	0.73	0.827	0.691	1.487	1.482	1.34
P84099	60S ribosomal protein	Rpl19	0.49	6.23239E-05	Down	0.726	0.736	0.82	1.57	1.579	1.512
Q7TNC4	Putative RNA-binding protein Luc7-like 2	Luc7l2	0.731	0.000609311	Down	0.863	0.879	0.906	1.254	1.141	1.228

P50136	2-oxoisovalerate dehydrogenase subunit alpha, mitochondrial	Bckdha	1.387	0.000235888	Up	1.211	1.164	1.178	0.822	0.848	0.891
P61514	60S ribosomal protein L37a	Rpl37a	0.535	6.85567E-05	Down	0.708	0.793	0.719	1.407	1.379	1.364
Q2EMV9	Protein mono-ADP-ribosyltransferase	Parp14	0.648	0.000862973	Down	0.762	0.883	0.769	1.218	1.259	1.246
Q60953	Protein PML	Pml	0.687	0.000330093	Down	0.931	0.85	0.84	1.261	1.272	1.28
Q8CGC6	RNA-binding protein 28	Rbm28	0.657	0.000983902	Down	0.896	0.807	0.819	1.313	1.335	1.19
Q9ET26	E3 ubiquitin-protein ligase RNF114	Rnf114	0.603	0.016053518	Down	0.845	0.601	0.854	1.338	1.346	1.131
P48025	Tyrosine-protein kinase SYK	Syk	0.54	0.000107274	Down	0.777	0.772	0.706	1.39	1.33	1.455
Q9R0B6	Laminin subunit	Lamc3	0.541	0.002269686	Down	0.761	0.707	0.617	1.369	1.356	1.126
Q791V5	Mitochondrial carrier homolog 2	Mtch2	1.348	0.000874801	Up	1.268	1.218	1.234	0.87	0.97	0.92
Q6PDM1	Male-specific lethal 1 homolog	Msl1	0.421	0.002042469	Down	0.531	0.734	0.693	1.77	1.48	1.403
O88441	Metaxin-2	Mtx2	1.602	0.000111563	Up	1.266	1.383	1.339	0.803	0.835	0.852
Q8CIH5	1-phosphatidylinositol 4,5-bisphosphate phosphodiesterase gamma-2	Plcg2	0.75	7.14884E-06	Down	0.863	0.849	0.869	1.133	1.158	1.152
P70388	DNA repair protein RAD50	Rad50	0.668	0.001103318	Down	0.869	0.801	0.808	1.148	1.32	1.241
Q6ZWV3	60S ribosomal protein	Rpl10	0.625	0.00027003	Down	0.827	0.811	0.73	1.277	1.258	1.256
P11928	2'-5'-oligoadenylate synthase 1A	Oas1a	0.523	0.000743552	Down	0.856	0.758	0.686	1.41	1.55	1.441
Q9CRD0	OCIA domain-containing protein 1	Ociad1	1.381	0.000107461	Up	1.165	1.207	1.224	0.843	0.888	0.872
Q3UU35	Ovostatin homolog	Ovos	1.311	8.84946E-05	Up	1.288	1.254	1.302	0.999	0.956	0.976
Q99PV0	Pre-mRNA-processing-splicing factor 8	Prpf8	0.751	0.000198415	Down	0.826	0.89	0.856	1.132	1.146	1.147
P19253	60S ribosomal protein L13a	Rpl13a	0.574	9.89536E-06	Down	0.8	0.815	0.795	1.349	1.411	1.435
P61358	60S ribosomal protein	Rpl27	0.53	6.1212E-05	Down	0.752	0.704	0.672	1.31	1.328	1.38
P62889	60S ribosomal protein	Rpl30	0.619	6.838E-05	Down	0.796	0.786	0.814	1.282	1.236	1.352
P62264	40S ribosomal protein	Rps14	0.558	0.000608703	Down	0.813	0.75	0.688	1.273	1.434	1.324
P62242	40S ribosomal protein	Rps8	0.514	4.35741E-05	Down	0.716	0.744	0.706	1.378	1.491	1.346
Q8C4H2	Sterile alpha motif domain-containing	Samd3	1.315	0.019590928	Up	1.07	1.218	1.131	0.813	0.979	0.808
Q922B1	ADP-ribose glycohydrolase	Macrod1	1.504	8.25877E-05	Up	1.224	1.199	1.211	0.826	0.767	0.824
Q91V01	Lysophospholipid acyltransferase 5	Lpcat3	1.433	0.00014373	Up	1.18	1.215	1.186	0.855	0.85	0.794
Q3URS9	Mitochondrial potassium channel	Ccdc51	1.39	0.004532686	Up	1.275	1.293	1.263	0.924	1.007	0.826
P43136	Nuclear receptor subfamily 2 group F	Nr2f6	0.734	0.004260688	Down	0.959	0.9	0.861	1.324	1.143	1.24
P03899	NADH-ubiquinone oxidoreductase chain 3	Mtnd3	0.762	0.034806046	Down	1.038	1.023	0.808	1.171	1.305	1.287
Q9CY58	Plasminogen activator inhibitor 1 RNA-binding protein	Serbp1	0.587	0.001440195	Down	0.887	0.863	0.786	1.285	1.555	1.479
Q8BGC4	Prostaglandin reductase-	Zadh2	1.378	0.001239833	Up	1.249	1.166	1.208	0.88	0.823	0.926
Q9D855	Cytochrome b-c1 complex subunit 7	Uqcrb	1.324	6.09273E-05	Up	1.213	1.249	1.198	0.938	0.921	0.906
Q9DBN5	Lon protease homolog 2, peroxisomal	Lonp2	1.614	0.000740876	Up	1.454	1.356	1.242	0.872	0.834	0.804
P49718	DNA replication licensing factor MCM5	Mcm5	0.664	0.007057955	Down	0.761	0.891	0.675	1.161	1.142	1.203
P39655	Polyunsaturated fatty acid lipoxygenase	Alox12	0.588	0.004228107	Down	0.853	0.805	0.7	1.356	1.482	1.171
Q9CQL1	Protein mago nashi homolog 2	Magohb	0.658	0.000104677	Down	0.81	0.792	0.794	1.277	1.17	1.196
P11103	Poly [ADP-ribose] polymerase 1	Parp1	0.537	4.34331E-06	Down	0.714	0.734	0.72	1.324	1.39	1.324
P30412	Peptidyl-prolyl cis-trans isomerase C	Ppic	0.74	0.000717169	Down	0.791	0.816	0.74	1.086	1.047	1.037
Q4FK66	Pre-mRNA-splicing factor 38A	Prpf38a	0.605	0.000578151	Down	0.825	0.77	0.704	1.215	1.308	1.28
Q80XL6	Acyl-CoA dehydrogenase family	Acad11	1.363	0.000255523	Up	1.374	1.278	1.378	0.975	0.992	0.99
Q5EG47	5'-AMP-activated protein kinase catalytic subunit alpha-1	Prkaa1	1.367	0.020790457	Up	1.14	1.119	1.084	0.702	0.802	0.942

Q8BK64	Activator of 90 kDa heat shock protein ATPase homolog 1	Ahsa1	0.753	0.001761414	Down	0.81	0.882	0.912	1.124	1.183	1.149
Q9Z1K6	E3 ubiquitin-protein ligase ARIH2	Arih2	0.705	0.035069249	Down	0.734	1.017	0.769	1.099	1.31	1.166
Q9D8B3	Charged multivesicular body protein 4b	Chmp4b	0.754	7.40882E-05	Down	0.845	0.854	0.822	1.122	1.134	1.088
Q9JHI5	Isovaleryl-CoA dehydrogenase, mitochondrial	Ivd	1.338	3.41929E-06	Up	1.208	1.234	1.204	0.906	0.906	0.912
O35900	U6 snRNA-associated Sm-like protein LSm2	Lsm2	0.761	0.021997276	Down	0.751	0.941	0.876	1.193	1.132	1.051
P14430	H-2 class I histocompatibility antigen, Q8 alpha chain	H2-Q8	0.719	3.30629E-05	Down	0.958	0.94	0.975	1.301	1.342	1.354
Q3THW5	Histone H2A.V	H2az2	0.371	1.43807E-06	Down	0.654	0.665	0.642	1.83	1.723	1.73
Q9D2U9	Histone H2B type 3-A	Hist3h2b	0.491	0.00015637	Down	0.758	0.842	0.784	1.749	1.587	1.524
P84228	Histone H3.2	H3c2	0.139	3.37626E-05	Down	0.384	0.35	0.278	2.481	2.444	2.368
Q8VEK3	Heterogeneous nuclear ribonucleoprotein U	Hnrnpu	0.724	1.11564E-06	Down	0.853	0.845	0.834	1.162	1.166	1.168
Q64282	Interferon-induced protein with tetratricopeptide repeats	Ifit1	0.392	0.000314288	Down	0.758	0.613	0.586	1.719	1.641	1.629
Q811L6	Microtubule-associated serine/threonine-protein kinase 4	Mast4	0.628	0.017247409	Down	0.982	0.748	0.816	1.239	1.594	1.219
Q8CAQ8	MICOS complex subunit Mic60	Immt	1.631	6.47268E-06	Up	1.383	1.329	1.371	0.837	0.819	0.848
Q9CZB0	Succinate dehydrogenase cytochrome b560	Sdhc	1.314	0.021971277	Up	1.278	1.043	1.296	0.891	0.96	0.901
A2A4P0	ATP-dependent RNA helicase DHX8	Dhx8	0.766	0.007324745	Down	0.944	0.925	0.828	1.255	1.14	1.127
Q80W21	Glutathione S-transferase Mu 7	Gstm7	1.514	0.001981231	Up	1.023	1.24	1.105	0.753	0.748	0.723
P43275	Histone H1.1	H1-1	0.224	3.88434E-05	Down	0.543	0.42	0.455	2.141	2.095	2.105
P01872	Immunoglobulin heavy constant mu	Ighm	1.609	0.000266977	Up	1.079	1.014	1.008	0.647	0.604	0.676
Q9CPU0	Lactoylglutathione lyase	Glo1	0.756	0.000192606	Down	0.846	0.903	0.856	1.166	1.142	1.14
P60853	Leucine zipper putative tumor suppressor 1	Lzts1	0.541	0.001929964	Down	0.793	0.88	0.66	1.463	1.39	1.457
Q9Z2Z6	Mitochondrial carnitine/acylcarnitine carrier protein	Slc25a20	1.575	3.87045E-05	Up	1.331	1.265	1.308	0.801	0.827	0.851
B2RPV6	Multimerin-1	Mmrn1	0.601	6.00037E-05	Down	0.89	0.854	0.868	1.436	1.52	1.39
O35615	Zinc finger protein	Zfpm1	0.766	0.023466307	Down	0.742	0.947	0.867	1.166	1.108	1.064
P01921	H-2 class II histocompatibility antigen, A-D beta chain	H2-Ab1	0.383	3.61082E-05	Down	0.752	0.688	0.644	1.772	1.8	1.873
Q3U1G5	Interferon-stimulated 20 kDa exonuclease-like 2	Isg20l2	0.587	0.008878755	Down	0.666	0.983	0.811	1.441	1.409	1.341
P08249	Malate dehydrogenase, mitochondrial	Mdh2	1.345	2.15818E-06	Up	1.224	1.222	1.236	0.913	0.922	0.902
P43247	DNA mismatch repair protein Msh2	Msh2	0.745	0.00052597	Down	0.858	0.846	0.826	1.137	1.078	1.182
Q61554	Fibrillin-1	Fbn1	1.457	1.27185E-05	Up	1.193	1.145	1.196	0.813	0.806	0.807
Q8BH70	F-box/LRR-repeat protein 4	Fbxl4	2.616	9.31205E-05	Up	1.738	1.822	1.515	0.618	0.649	0.673
P98192	Dihydroxyacetone phosphate	Gnpat	1.656	0.000121636	Up	1.262	1.404	1.39	0.816	0.816	0.818
Q80SU7	Interferon-induced very large GTPase 1	Gvin1	0.574	3.43221E-06	Down	0.858	0.826	0.816	1.454	1.458	1.446
Q61425	Hydroxyacyl-coenzyme A dehydrogenase, mitochondrial	Hadh	1.406	5.74545E-06	Up	1.232	1.237	1.257	0.892	0.868	0.89
Q61696	Heat shock 70 kDa protein 1A	Hspa1a	0.66	0.029145507	Down	0.861	0.897	0.874	1.662	1.197	1.131
Q5EBG6	Heat shock protein beta-	Hspb6	0.719	0.002001856	Down	0.857	0.852	0.858	1.222	1.262	1.086
P11835	Integrin beta-2	Itgb2	0.748	0.016278287	Down	0.724	0.659	0.575	0.918	0.826	0.872
Q8K310	Matrin-3	Matr3	0.689	1.35824E-05	Down	0.798	0.8	0.798	1.162	1.13	1.188
P21843	Mast cell protease 3 (Fragment)	Mcpt3	0.277	0.000753556	Down	0.605	0.646	0.413	2.063	2.015	1.939
Q78J03	Methionine-R-sulfoxide reductase B2,	Msrb2	1.386	0.0001586	Up	1.208	1.244	1.276	0.926	0.872	0.892
Q9DAU1	Protein canopy homolog	Cnpy3	0.719	2.84704E-05	Down	0.896	0.933	0.887	1.259	1.258	1.263
P19536	Cytochrome c oxidase subunit 5B,	Cox5b	1.337	0.000129345	Up	1.239	1.283	1.233	0.96	0.939	0.91

Q9CXV1	Succinate dehydrogenase [ubiquinone]	Sdhd	1.482	0.00217678	Up	1.208	1.403	1.176	0.858	0.861	0.836
O35459	Delta(3,5)-Delta(2,4)-dienoyl-CoA isomerase, mitochondrial	Ech1	1.901	6.77988E-07	Up	1.384	1.415	1.426	0.734	0.752	0.736
P23336	N-acetyllactosaminide alpha-1,3-galactosyltransferase	Ggta1	1.39	0.003953816	Up	1.171	1.075	1.036	0.723	0.823	0.815
Q8BMF3	NADP-dependent malic enzyme, mitochondrial	Me3	1.348	0.000166117	Up	1.193	1.248	1.282	0.911	0.93	0.921
P26645	Myristoylated alanine-rich C-kinase substrate	Marcks	0.573	0.004250461	Down	0.836	0.89	0.761	1.591	1.528	1.22
Q80XN0	D-beta-hydroxybutyrate dehydrogenase, mitochondrial	Bdh1	0.497	3.02764E-05	Down	0.738	0.791	0.765	1.553	1.6	1.462
Q8CGZ0	Calcium homeostasis endoplasmic reticulum protein	Cherp	0.747	0.009070963	Down	0.848	0.934	0.77	1.12	1.2	1.096
Q9R1Y5	Hypermethylated in cancer 1 protein	Hic1	0.378	8.33768E-05	Down	0.622	0.631	0.577	1.697	1.699	1.449
Q00PI9	Heterogeneous nuclear ribonucleoprotein U-like protein 2	Hnrnpul2	0.644	7.39966E-06	Down	0.798	0.802	0.768	1.226	1.215	1.238
P28740	Kinesin-like protein	Kif2a	0.661	0.000782979	Down	0.878	0.767	0.79	1.216	1.273	1.194
P55200	Histone-lysine N-methyltransferase 2A	Kmt2a	0.745	0.003965154	Down	0.813	0.927	0.941	1.163	1.199	1.235
Q9DA03	Complex III assembly factor LYRM7	Lyrm7	1.335	0.003056538	Up	1.195	1.166	1.211	0.94	0.92	0.816
Q6ZQ73	Cullin-associated NEDD8-dissociated	Cand2	1.385	0.006031155	Up	1.308	1.328	1.101	0.91	0.887	0.902
P97821	Dipeptidyl peptidase 1	Ctsc	0.756	0.021167473	Down	0.68	0.694	0.848	1.032	0.925	0.984
Q6Q899	Antiviral innate immune response receptor RIG-I	Ddx58	0.751	1.77404E-06	Down	0.881	0.882	0.868	1.175	1.168	1.158
Q3UIR3	E3 ubiquitin-protein ligase DTX3L	Dtx3l	0.637	0.000494047	Down	0.9	0.814	0.79	1.333	1.336	1.261
Q3UN02	Lysocardiolipin acyltransferase 1	Lclat1	1.388	0.000878557	Up	1.222	1.132	1.2	0.812	0.851	0.897
Q9DCX2	ATP synthase subunit d, mitochondrial	Atp5pd	1.405	1.4883E-05	Up	1.245	1.241	1.264	0.894	0.906	0.869
Q07813	Apoptosis regulator	Bax	0.673	0.001518967	Down	0.865	0.816	0.76	1.19	1.146	1.291
Q69ZF3	Non-lysosomal glucosylceramidase	Gba2	1.325	0.006948138	Up	1.153	1.101	1.171	0.801	0.832	0.952
Q3TC93	HCLS1-binding protein	Hs1bp3	0.74	0.006548617	Down	0.921	1.037	0.856	1.251	1.24	1.311
P15864	Histone H1.2	H1-2	0.275	0.001069321	Down	0.556	0.746	0.437	2.13	2.162	2.042
P17879	Heat shock 70 kDa protein 1B	Hspa1b	0.561	0.00222035	Down	0.803	0.96	0.749	1.382	1.571	1.524
P07901	Heat shock protein HSP 90-alpha	Hsp90aa	0.655	1.11825E-05	Down	0.827	0.826	0.796	1.236	1.232	1.269
Q69ZA1	Cyclin-dependent kinase	Cdk13	0.6	0.015426603	Down	0.741	1.055	0.759	1.53	1.259	1.472
P62806	Histone H4	H4c1	0.155	2.99714E-05	Down	0.398	0.391	0.303	2.388	2.326	2.31
Q9CY57	Chromatin target of PRMT1 protein	Chtop	0.531	0.001253299	Down	0.75	0.856	0.711	1.532	1.534	1.299
P11087	Collagen alpha-1(I)	Col1a1	1.56	0.003717024	Up	1.02	1.224	1.118	0.671	0.692	0.792
Q9QYB2	Dachshund homolog 1	Dach1	0.695	0.006342414	Down	0.76	0.93	0.776	1.19	1.238	1.122
Q8BK48	Pyrethroid hydrolase	Ces2e	0.56	4.88786E-06	Down	0.788	0.749	0.764	1.391	1.347	1.371
O54890	Integrin beta-3	Itgb3	0.288	1.05053E-05	Down	0.557	0.552	0.485	1.877	1.844	1.823
A2CG49	Kalirin	Kalrn	0.597	0.000215432	Down	0.863	0.808	0.76	1.345	1.401	1.324
P60824	Cold-inducible RNA-binding protein	Cirbp	1.438	4.7395E-05	Up	1.112	1.102	1.08	0.739	0.768	0.784
Q99LC5	Electron transfer flavoprotein subunit alpha, mitochondrial	Etfa	1.37	2.18456E-06	Up	1.255	1.268	1.254	0.931	0.915	0.91
Q9CY66	H/ACA ribonucleoprotein	Gar1	0.741	0.001944307	Down	0.94	0.852	0.932	1.172	1.216	1.286
Q9R1K9	Centrin-2	Cetn2	0.724	0.005779075	Down	0.936	0.961	0.843	1.356	1.266	1.162
P06537	Glucocorticoid receptor	Nr3c1	0.518	0.000451255	Down	0.707	0.824	0.685	1.468	1.355	1.454
Q62469	Integrin alpha-2	Itga2	0.463	0.001069674	Down	0.756	0.774	0.586	1.552	1.456	1.561
Q03963	Interferon-induced, double-stranded RNA-activated protein kinase	Eif2ak2	0.624	0.000412091	Down	0.804	0.849	0.815	1.353	1.386	1.216
Q9DCW4	Electron transfer flavoprotein subunit beta	Etfb	1.34	0.000164075	Up	1.241	1.189	1.258	0.903	0.908	0.941
P20491	High affinity immunoglobulin epsilon receptor subunit gamma	Fcer1g	0.409	5.43606E-05	Down	0.586	0.518	0.509	1.367	1.302	1.27
Q60634	Flotillin-2	Flot2	1.498	9.12109E-06	Up	1.06	1.049	1.084	0.696	0.719	0.717

P54071	Isocitrate dehydrogenase [NADP], mitochondrial	Idh2	1.505	1.14836E-06	Up	1.263	1.244	1.272	0.828	0.84	0.843
P97864	Caspase-7	Casp7	0.727	0.000168271	Down	0.889	0.85	0.844	1.153	1.222	1.18
Q9CZT6	Protein CMSS1	Cmss1	0.627	6.60394E-05	Down	0.828	0.816	0.764	1.307	1.269	1.262
P01027	Complement C3	C3	1.405	0.00020286	Up	1.104	1.117	1.12	0.791	0.829	0.758
Q9CRA8	Exosome complex component RRP46	Exosc5	0.665	0.001051869	Down	0.744	0.804	0.873	1.214	1.243	1.184
Q3TEA8	Heterochromatin protein 1-binding protein 3	Hp1bp3	0.438	8.32133E-05	Down	0.72	0.764	0.644	1.644	1.594	1.624
P0DOV2	Interferon-activable protein 204	Ifi204	0.484	3.13496E-05	Down	0.678	0.742	0.663	1.424	1.45	1.428
Q9D2D9	Kelch domain-containing protein 8B	Klhdc8b	1.311	0.008822426	Up	1.183	1.367	1.236	1.026	0.959	0.902
Q2VLH6	Scavenger receptor cysteine-rich type 1 protein M130	Cd163	0.705	0.031578821	Down	0.649	0.81	0.62	0.985	0.863	1.1
P56393	Cytochrome c oxidase subunit 7B,	Cox7b	1.325	0.000433242	Up	1.303	1.342	1.228	0.978	0.976	0.969
Q9Z2A9	Glutathione hydrolase 5 proenzyme	Ggt5	1.533	0.000106006	Up	1.071	1.114	1.013	0.69	0.697	0.699
Q07235	Glia-derived nexin	Serpine2	0.438	9.57876E-05	Down	0.743	0.657	0.721	1.573	1.736	1.537
Q99MJ9	ATP-dependent RNA helicase DDX50	Ddx50	0.639	0.00018264	Down	0.82	0.895	0.826	1.277	1.352	1.348
Q8BHC4	Dephospho-CoA kinase domain-containing	Dcakd	1.439	0.000351077	Up	1.305	1.192	1.269	0.855	0.859	0.904
P42125	Enoyl-CoA delta isomerase 1,	Eci1	1.383	6.31794E-07	Up	1.236	1.245	1.226	0.887	0.896	0.898
Q9D7X8	Gamma-glutamylcyclotransferase	Ggct	0.752	0.015621612	Down	0.772	0.951	0.887	1.239	1.132	1.099
Q2TPA8	Hydroxysteroid dehydrogenase-like protein 2	Hsdl2	1.416	4.54865E-06	Up	1.215	1.217	1.238	0.851	0.864	0.876
Q99M71	Mammalian ependymin-related protein 1	Epdr1	0.724	0.010648354	Down	0.993	0.806	0.93	1.176	1.256	1.338
Q91WK5	Glycine cleavage system H protein, mitochondrial	Gcsh	1.465	0.001758615	Up	1.18	1.27	1.251	0.796	0.809	0.922
Q8VDM6	Heterogeneous nuclear ribonucleoprotein U-like protein 1	Hnrnpul1	0.69	5.65929E-05	Down	0.856	0.822	0.836	1.248	1.177	1.217
Q9QZ85	Interferon-inducible GTPase 1	Iigp1	0.477	2.86035E-06	Down	0.821	0.769	0.787	1.65	1.67	1.661
Q8R3P0	Aspartoacylase	Aspa	0.699	0.000416492	Down	0.922	0.861	0.842	1.273	1.277	1.205
Q8JZX9	Cdc42 effector protein 2	Cdc42ep	0.675	0.008935203	Down	0.861	0.907	0.814	1.294	1.427	1.102
P24270	Catalase	Cat	1.682	0.00010164	Up	1.441	1.294	1.342	0.79	0.816	0.818
P29391	Ferritin light chain 1	Ftl1	0.687	0.007936346	Down	0.467	0.53	0.412	0.713	0.652	0.685
Q9QUG9	RAS guanyl-releasing protein 2	Rasgrp2	0.748	0.039277508	Down	0.896	0.984	0.711	1.13	1.204	1.128
Q8C6K9	Collagen alpha-6(VI)	Col6a6	0.709	0.000634207	Down	0.936	0.868	0.854	1.207	1.243	1.3
O54942	Claudin-5	Cldn5	1.439	0.000466992	Up	1.216	1.36	1.24	0.88	0.888	0.883
O35218	Cleavage and polyadenylation specificity factor subunit	Cpsf2	0.765	0.010239701	Down	0.956	0.872	0.818	1.126	1.238	1.094
P10922	Histone H1.0	H1-0	0.179	6.64656E-05	Down	0.465	0.432	0.336	2.362	2.314	2.21
Q9D1N2	Glycosylphosphatidylinositol-anchored high density lipoprotein-binding protein 1	Gpihbp1	1.893	1.04396E-05	Up	1.536	1.506	1.422	0.787	0.783	0.788
Q9Z204	Heterogeneous nuclear ribonucleoproteins	Hnrnpc	0.636	0.000569655	Down	0.811	0.781	0.774	1.216	1.344	1.162
P01867	Ig gamma-2B chain C region	Igh-3	0.623	0.000684153	Down	0.852	0.827	0.914	1.462	1.281	1.416
Q91VN6	Probable ATP-dependent RNA helicase	Ddx41	0.703	0.000147337	Down	0.853	0.853	0.854	1.158	1.225	1.261
Q99KI0	Aconitate hydratase, mitochondrial	Aco2	1.374	2.67615E-08	Up	1.254	1.251	1.258	0.915	0.909	0.914
P31786	Acyl-CoA-binding	Dbi	1.326	0.002800761	Up	0.996	1.037	1.143	0.809	0.808	0.778
Q8CFQ3	RNA helicase aquarius	Aqr	0.756	0.000514695	Down	0.824	0.896	0.855	1.159	1.14	1.109
O09161	Calsequestrin-2	Casq2	1.965	2.95613E-06	Up	1.461	1.49	1.497	0.736	0.75	0.778
Q8VCT4	Carboxylesterase 1D	Ces1d	1.934	0.000596915	Up	1.63	1.683	1.502	0.812	0.756	0.922
P02463	Collagen alpha-1(IV)	Col4a1	0.754	0.010167446	Down	0.954	0.888	0.787	1.21	1.105	1.171
Q08093	Calponin-2	Cnn2	0.479	0.000637138	Down	0.743	0.59	0.657	1.327	1.491	1.339
Q91WS0	CDGSH iron-sulfur domain-containing	Cisd1	1.393	0.000953572	Up	1.36	1.243	1.288	0.97	0.94	0.883
Q8BH61	Coagulation factor XIII A chain	F13a1	0.752	0.005237586	Down	0.638	0.706	0.596	0.889	0.847	0.844
P59266	Fat storage-inducing transmembrane protein 2	Fitm2	1.506	0.000148637	Up	1.262	1.324	1.341	0.874	0.833	0.9
Q9Z0E6	Guanylate-binding	Gbp2	0.595	7.75646E-05	Down	0.891	0.887	0.81	1.462	1.444	1.442

O54794	Aquaporin-7	Aqp7	1.671	3.98577E-05	Up	1.326	1.273	1.359	0.814	0.765	0.79
O54962	Barrier-to-autointegration factor	Banf1	0.598	0.001339253	Down	0.778	0.705	0.859	1.297	1.24	1.378
Q8K0E8	Fibrinogen beta chain	Fgb	0.478	5.75315E-08	Down	0.739	0.749	0.744	1.57	1.538	1.562
P26323	Friend leukemia integration 1	Fli1	1.505	0.027763983	Up	1.106	1.258	1.088	0.919	0.616	0.758
C0HKE9	Histone H2A type 1-P	Hist1h2a	0.165	0.000308176	Down	0.471	0.426	0.283	2.366	2.449	2.34
P01902	H-2 class I histocompatibility antigen, K-D alpha	H2-K1	0.452	1.93409E-07	Down	0.751	0.732	0.726	1.621	1.637	1.624
Q9D3B1	Very-long-chain (3R)-3-hydroxyacyl-CoA dehydratase 2	Hacd2	1.52	0.000482401	Up	1.443	1.391	1.262	0.898	0.894	0.903
Q61699	Heat shock protein 105 kDa	Hsph1	0.545	6.89793E-05	Down	0.822	0.765	0.749	1.487	1.42	1.38
Q01149	Collagen alpha-2(I)	Col1a2	1.621	0.000289083	Up	1.007	1.071	0.988	0.613	0.672	0.606
Q60649	Caseinolytic peptidase B protein homolog	Clpb	1.563	2.42195E-06	Up	1.294	1.297	1.29	0.812	0.827	0.844
Q61164	Transcriptional repressor CTCF	Ctcf	0.593	2.06348E-05	Down	0.833	0.8	0.776	1.377	1.334	1.348
Q61107	Guanylate-binding	Gbp4	0.463	6.63729E-05	Down	0.753	0.767	0.672	1.618	1.527	1.585
P56400	Platelet glycoprotein Ib beta chain	Gp1bb	0.391	2.58866E-05	Down	0.682	0.624	0.658	1.561	1.722	1.737
P30681	High mobility group protein B2	Hmgb2	0.56	0.000462302	Down	0.836	0.846	0.762	1.418	1.581	1.365
Q9CQ00	Distal membrane-arm assembly complex	Dmac1	1.639	0.000803687	Up	1.288	1.378	1.53	0.879	0.86	0.821
Q9DBM2	Peroxisomal bifunctional enzyme	Ehhadh	1.389	0.006297675	Up	1.337	1.315	1.103	0.908	0.906	0.889
Q80VD1	Protein FAM98B	Fam98b	0.751	0.019154089	Down	0.792	0.939	1.017	1.264	1.174	1.222
Q8R0F8	Acylpyruvase FAHD1, mitochondrial	Fahd1	1.383	0.000754741	Up	1.3	1.207	1.216	0.853	0.93	0.909
O08917	Flotillin-1	Flot1	1.418	6.161E-05	Up	1.042	1.086	1.04	0.756	0.724	0.754
Q91WG8	Bifunctional UDP-N-acetylglucosamine 2-epimerase/N-acetylmannosamine	Gne	0.75	0.002066763	Down	0.886	0.967	0.883	1.152	1.262	1.235
Q9D1G3	Protein-cysteine N-palmitoyltransferase HHAT-like protein	Hhatl	2.064	0.000757112	Up	1.45	1.616	1.454	0.692	0.664	0.834
P34914	Bifunctional epoxide hydrolase 2	Ephx2	1.32	4.18106E-05	Up	1.205	1.207	1.231	0.931	0.932	0.897
P97465	Docking protein 1	Dok1	0.748	7.43479E-05	Down	0.876	0.85	0.894	1.18	1.146	1.176
P43276	Histone H1.5	H1-5	0.375	0.000397652	Down	0.747	0.694	0.557	1.745	1.849	1.737
Q3U5Q7	UMP-CMP kinase 2, mitochondrial	Cmpk2	0.751	4.19744E-05	Down	0.879	0.858	0.882	1.19	1.145	1.152
Q8R1S0	Ubiquinone biosynthesis monooxygenase COQ6, mitochondrial	Coq6	1.311	0.00335072	Up	1.147	1.284	1.263	0.972	0.952	0.894
O70585	Dystrobrevin beta	Dtnb	1.608	0.013709022	Up	1.093	0.883	1.107	0.557	0.612	0.748
Q3TZW0	Endothelial cell-specific chemotaxis regulator	Ecscr	1.51	0.002779497	Up	1.253	1.072	1.216	0.721	0.8	0.824
Q811I0	ATP synthase mitochondrial F1 complex assembly factor	Atpaf1	1.31	0.000568784	Up	1.22	1.12	1.208	0.904	0.901	0.903
Q921G7	Electron transfer flavoprotein-ubiquinone oxidoreductase, mitochondrial	Etfdh	1.346	6.53177E-06	Up	1.224	1.24	1.246	0.926	0.904	0.926
P28798	Progranulin	Grn	0.72	0.019753133	Down	0.791	0.95	0.946	1.284	1.35	1.096
P43274	Histone H1.4	H1-4	0.253	0.000129717	Down	0.572	0.54	0.422	2.023	1.958	2.086
Q6PGH1	Protein BUD31 homolog	Bud31	0.746	0.003562886	Down	0.787	0.872	0.866	1.063	1.124	1.197
Q1ERP8	CMRF35-like molecule	Cd300lg	1.598	9.20399E-07	Up	1.23	1.212	1.193	0.76	0.754	0.761
P30416	Peptidyl-prolyl cis-trans isomerase FKBP4	Fkbp4	0.732	0.000395439	Down	0.859	0.9	0.891	1.188	1.166	1.265
Q920E5	Farnesyl pyrophosphate synthase	Fdps	0.744	0.008461435	Down	0.804	0.866	0.878	1.255	1.04	1.131
Q99PU8	ATP-dependent RNA helicase DHX30	Dhx30	0.749	0.010425875	Down	0.796	0.834	0.938	1.117	1.235	1.077
Q91V16	Electron transfer flavoprotein regulatory factor 1	Etfrf1	1.333	0.000335072	Up	1.238	1.221	1.189	0.872	0.929	0.935
Q9D6M3	Mitochondrial glutamate carrier 1	Slc25a22	1.357	0.000148904	Up	1.21	1.276	1.221	0.888	0.933	0.91
Q9Z1E4	Glycogen [starch] synthase, muscle	Gys1	1.303	0.000658549	Up	1.193	1.157	1.149	0.931	0.9	0.854
P56391	Cytochrome c oxidase subunit 6B1	Cox6b1	1.725	3.6386E-07	Up	1.378	1.382	1.393	0.809	0.79	0.809

Q8VCM7	Fibrinogen gamma chain	Fgg	0.549	4.77603E-05	Down	0.791	0.86	0.782	1.488	1.444	1.498
Q61599	Rho GDP-dissociation inhibitor 2	Arhgdib	0.736	0.000212859	Down	0.771	0.822	0.816	1.064	1.103	1.108
Q9JI91	Alpha-actinin-2	Actn2	1.31	1.63612E-05	Up	1.214	1.182	1.209	0.913	0.93	0.908
P18293	Atrial natriuretic peptide receptor 1	Npr1	1.317	0.007413486	Up	1.121	1.256	1.239	0.934	0.969	0.843
O70423	Membrane primary amine oxidase	Aoc3	1.566	1.55678E-05	Up	1.089	1.116	1.097	0.705	0.682	0.722
Q08509	Epidermal growth factor receptor kinase substrate	Eps8	1.376	6.57434E-05	Up	1.087	1.088	1.066	0.804	0.759	0.792
Q91WM6	Protein eva-1 homolog	Eva1a	1.592	4.11035E-05	Up	1.208	1.122	1.192	0.73	0.748	0.735
Q91V79	Fat storage-inducing transmembrane protein 1	Fitm1	1.632	0.001254545	Up	1.413	1.327	1.556	0.884	0.932	0.816
Q9D164	FXYD domain-containing ion transport	Fxyd6	0.64	0.000663978	Down	0.703	0.778	0.792	1.251	1.14	1.162
P51174	Long-chain specific acyl-CoA dehydrogenase,	Acadl	1.419	3.11844E-06	Up	1.243	1.284	1.265	0.89	0.892	0.89
Q9Z277	Tyrosine-protein kinase BAZ1B	Baz1b	0.581	0.000328578	Down	0.799	0.816	0.721	1.284	1.412	1.325
O08739	AMP deaminase 3	Ampd3	0.734	0.002081805	Down	0.899	0.8	0.89	1.226	1.163	1.136
P56382	ATP synthase subunit epsilon, mitochondrial	Atp5f1e	1.337	0.007333529	Up	1.356	1.134	1.227	0.973	0.906	0.902
P51637	Caveolin-3	Cav3	1.483	0.020877648	Up	1.357	0.989	1.34	0.817	0.824	0.844
Q9D6U8	Protein FAM162A	Fam162a	1.331	0.001566665	Up	1.173	1.306	1.295	0.921	0.967	0.947
P47934	Carnitine O-acetyltransferase	Crat	1.376	1.5979E-06	Up	1.24	1.218	1.246	0.895	0.901	0.895
Q8K199	COX assembly mitochondrial protein 2 homolog	Cmc2	1.389	0.002698563	Up	1.465	1.24	1.368	0.978	0.993	0.961
O70433	Four and a half LIM domains protein 2	Fhl2	1.516	0.000671332	Up	1.203	1.062	1.136	0.747	0.717	0.78
Q9Z0G0	PDZ domain-containing protein GIPC1	Gipc1	0.662	0.006420113	Down	0.952	0.758	0.789	1.352	1.197	1.224
E9PZM4	Chromodomain-helicase-DNA-binding	Chd2	0.287	0.000186362	Down	0.657	0.574	0.507	1.903	2.257	1.898
Q8CIM7	Cytochrome P450 2D26	Cyp2d26	1.376	0.01115752	Up	1.045	1.094	1.151	0.866	0.829	0.696
Q8C0L8	Conserved oligomeric Golgi complex subunit 5	Cog5	0.727	0.03789841	Down	0.872	0.888	0.786	1.049	1.396	1.055
Q9JI44	DNA methyltransferase 1-associated protein 1	Dmap1	0.728	0.000284249	Down	0.89	0.835	0.828	1.162	1.145	1.2
Q8BH95	Enoyl-CoA hydratase, mitochondrial	Echs1	1.349	4.87105E-05	Up	1.275	1.22	1.242	0.931	0.906	0.934
P15379	CD44 antigen	Cd44	0.478	6.47113E-05	Down	0.727	0.658	0.72	1.412	1.549	1.439
Q99388	Component of Sp100-rs	Csprs	0.483	0.001990641	Down	0.809	0.698	0.734	1.838	1.391	1.411
Q3UZA1	CapZ-interacting protein	Rcsd1	0.742	0.001460336	Down	0.942	0.936	0.848	1.252	1.182	1.239
Q9CXV9	DCN1-like protein 5	Dcun1d5	0.747	0.007722076	Down	0.784	0.836	0.899	1.057	1.093	1.22
Q99JY0	Trifunctional enzyme subunit beta,	Hadhb	1.406	1.92959E-06	Up	1.18	1.212	1.198	0.847	0.854	0.852
Q8BIQ5	Cleavage stimulation factor subunit 2	Cstf2	0.764	0.007031259	Down	0.894	0.909	0.79	1.08	1.12	1.194
P00184	Cytochrome P450 1A1	Cyp1a1	1.6	4.32347E-05	Up	1.53	1.508	1.623	0.976	0.953	0.984
O89103	Complement component C1q receptor	Cd93	1.5	0.035983892	Up	0.918	1.384	1.061	0.78	0.691	0.771
Q99NF3	Centrosomal protein of 41 kDa	Cep41	0.709	0.028169577	Down	0.967	1.02	0.817	1.132	1.467	1.356
P51660	Peroxisomal multifunctional enzyme	Hsd17b4	1.313	0.001665005	Up	1.247	1.113	1.17	0.922	0.889	0.878
Q64237	Dopamine beta-hydroxylase	Dbh	1.371	0.044305409	Up	0.86	1.005	1.222	0.707	0.773	0.771
P15089	Mast cell carboxypeptidase A	Cpa3	0.37	8.50488E-05	Down	0.687	0.672	0.605	1.638	1.927	1.747
Q921W0	Charged multivesicular body protein 1a	Chmp1a	0.76	0.001242031	Down	0.913	0.884	0.818	1.167	1.133	1.14
P97478	5-demethoxyubiquinone hydroxylase,	Coq7	1.317	0.002183742	Up	1.246	1.234	1.318	1.02	0.955	0.908
Q64442	Sorbitol dehydrogenase	Sord	0.485	9.3719E-06	Down	0.784	0.724	0.741	1.571	1.518	1.546
P84089	Enhancer of rudimentary homolog	Erh	0.743	0.001036442	Down	0.884	0.83	0.794	1.152	1.092	1.13
P09813	Apolipoprotein A-II	Apoa2	1.708	0.018669749	Up	1.353	0.87	1.228	0.7	0.664	0.657
Q9Z1W8	Potassium-transporting ATPase alpha chain 2	Atp12a	1.339	0.000293429	Up	1.239	1.219	1.209	0.87	0.932	0.936
O54940	BCL2/adenovirus E1B 19 kDa protein-interacting protein 2	Bnip2	1.408	0.00011115	Up	1.093	1.09	1.108	0.79	0.802	0.746
P01887	Beta-2-microglobulin	B2m	0.467	3.32057E-05	Down	0.805	0.71	0.752	1.622	1.594	1.637
Q9CZU6	Citrate synthase, mitochondrial	Cs	1.412	9.95763E-07	Up	1.23	1.253	1.248	0.874	0.886	0.883

Q9CQ62	2,4-dienoyl-CoA reductase, mitochondrial	Decr1	1.428	8.28811E-05	Up	1.242	1.288	1.233	0.881	0.851	0.903
E9Q557	Desmoplakin	Dsp	1.809	1.35626E-06	Up	1.492	1.445	1.438	0.805	0.799	0.815
Q921E6	Polycomb protein EED	Eed	0.649	0.00209914	Down	0.716	0.845	0.85	1.227	1.192	1.297
P35550	rRNA 2'-O-methyltransferase	Fbl	0.53	1.48477E-05	Down	0.8	0.776	0.736	1.444	1.474	1.445
Q9D1F4	Proline-rich AKT1 substrate 1	Akt1s1	0.687	0.008206878	Down	0.705	0.739	0.817	1.237	1.044	1.009
P59017	Bcl-2-like protein 13	Bcl2l13	1.488	0.027819432	Up	1.282	0.992	1.371	0.727	0.827	0.896
O35855	Branched-chain-amino-acid aminotransferase, mitochondrial	Bcat2	1.324	5.41113E-05	Up	1.169	1.21	1.168	0.889	0.879	0.91
Q60997	Deleted in malignant brain tumors 1 protein	Dmbt1	2.862	0.000370042	Up	1.755	1.37	1.709	0.554	0.517	0.618
Q3TXU5	Deoxyhypusine synthase	Dhps	1.303	0.015555965	Up	1.213	0.999	1.129	0.906	0.813	0.845
Q9WUR2	Enoyl-CoA delta isomerase 2,	Eci2	1.707	8.80716E-06	Up	1.325	1.349	1.35	0.762	0.81	0.785
Q00623	Apolipoprotein A-I	Apoa1	2.152	4.32107E-06	Up	1.206	1.186	1.25	0.546	0.574	0.572
Q9CXW3	Calcyclin-binding	Cacybp	0.707	0.000292325	Down	0.864	0.877	0.8	1.181	1.209	1.204
P98078	Disabled homolog 2	Dab2	0.759	0.001611111	Down	0.766	0.865	0.804	1.088	1.064	1.056
Q8BGB7	Enolase-phosphatase E1	Enoph1	0.183	0.000477073	Down	0.494	0.39	0.306	2.154	2.504	1.846
Q8VC31	Coiled-coil domain-containing protein 9	Ccdc9	0.701	0.000288295	Down	0.848	0.848	0.778	1.168	1.196	1.164
O88271	Craniofacial development protein 1	Cfdp1	0.736	0.008304923	Down	0.907	0.819	0.877	1.053	1.234	1.249
P00416	Cytochrome c oxidase subunit 3	mt-Co3	1.389	0.0007143	Up	1.264	1.383	1.255	0.917	0.927	0.965
Q8BMS4	Ubiquinone biosynthesis O-methyltransferase, mitochondrial	Coq3	1.472	0.000192373	Up	1.287	1.317	1.338	0.89	0.936	0.852
P00397	Cytochrome c oxidase subunit 1	Mtco1	1.7	0.000808078	Up	1.183	1.373	1.203	0.738	0.693	0.78
P70170	ATP-binding cassette sub-family C member 9	Abcc9	1.365	5.54845E-05	Up	1.133	1.16	1.096	0.83	0.833	0.819
Q9CS00	Cactin	Cactin	0.691	0.020741493	Down	0.761	0.754	0.973	1.294	1.235	1.072
P50172	Corticosteroid 11-beta-dehydrogenase isozyme	Hsd11b1	0.711	1.5457E-05	Down	0.896	0.925	0.911	1.281	1.303	1.258
Q9DBB1	Dual specificity protein phosphatase 6	Dusp6	0.446	0.022822087	Down	0.601	0.673		1.74	1.458	1.085
Q5DW34	Histone-lysine N-methyltransferase	Ehmt1	0.604	0.040525119	Down	0.608	0.949	0.575	1.142	1.04	1.348
Q9EPU4	Cleavage and polyadenylation specificity factor subunit	Cpsf1	0.704	0.002680295	Down	0.855	0.781	0.886	1.198	1.115	1.268
P47713	Cytosolic phospholipase A2	Pla2g4a	0.536	0.000836308	Down	0.609	0.711	0.768	1.308	1.327	1.264
Q99MZ7	Peroxisomal trans-2-enoyl-CoA reductase	Pecr	1.381	0.01015188	Up	1.195	1.188	1.337	0.854	1.007	0.833
Q8BP40	Lysophosphatidic acid phosphatase type 6	Acp6	1.445	9.93873E-06	Up	1.284	1.28	1.312	0.875	0.905	0.902
Q7TSC1	Protein PRRC2A	Prrc2a	0.577	0.002822184	Down	0.814	0.723	0.942	1.375	1.383	1.538
P14148	60S ribosomal protein	Rpl7	0.514	9.22952E-06	Down	0.766	0.749	0.71	1.462	1.442	1.428
P36536	GTP-binding protein SAR1a	Sar1a	1.303	0.03875108	Up	1.206	1.062	1.145	0.823	0.78	1.017
Q60714	Long-chain fatty acid transport protein 1	Slc27a1	3.031	5.76947E-05	Up	1.567	1.666	1.799	0.579	0.502	0.579
P31725	Protein S100-A9	S100a9	2.218	1.08112E-05	Up	1.284	1.299	1.372	0.586	0.578	0.619
Q64213	Splicing factor 1	Sf1	0.684	0.000344561	Down	0.828	0.785	0.749	1.138	1.19	1.126
Q8BHZ4	Zinc finger protein 592	Znf592	0.671	0.024205087	Down	0.965	0.713	0.804	1.365	1.267	1.069
P28650	Adenylosuccinate synthetase isozyme 1	Adss1	0.727	0.000270028	Down	0.81	0.756	0.749	1.051	1.082	1.052
P62911	60S ribosomal protein	Rpl32	0.59	0.000311356	Down	0.818	0.837	0.728	1.352	1.31	1.374
Q8BR65	Sin3 histone deacetylase corepressor complex component SDS3	Suds3	0.744	0.00241626	Down	0.792	0.859	0.898	1.141	1.096	1.187
Q8R2K4	TAF6-like RNA polymerase II p300/CBP-associated factor-associated factor 65 kDa subunit 6L	Taf6l	1.547	0.000134158	Up	1.37	1.304	1.244	0.86	0.846	0.826
Q61464	Zinc finger protein 638	Znf638	0.682	0.000389105	Down	0.833	0.853	0.9	1.275	1.203	1.316
P62702	40S ribosomal protein S4, X isoform	Rps4x	0.701	1.93737E-05	Down	0.818	0.837	0.803	1.178	1.182	1.148
Q9ERN0	Secretory carrier-associated membrane protein 2	Scamp2	0.729	0.035270937	Down	0.724	0.894	1.03	1.216	1.167	1.251
Q8C1Q6	Small integral membrane protein 4	Smim4	1.784	0.005060552	Up	1.471	1.334	1.558	0.67	0.869	0.907

Q4G0F8	Ubinuclein-1	Ubn1	0.542	0.000425826	Down	0.7	0.717	0.705	1.454	1.242	1.217
Q9ERU3	Zinc finger protein 22	Znf22	0.614	0.000844469	Down	0.904	0.771	0.857	1.3	1.42	1.404
Q8BKS9	Pumilio homolog 3	Pum3	0.695	0.023359314	Down	0.94	0.994	0.71	1.281	1.28	1.241
P12970	60S ribosomal protein	Rpl7a	0.633	2.39168E-05	Down	0.832	0.835	0.846	1.324	1.369	1.278
P62320	Small nuclear ribonucleoprotein Sm	Snrpd3	0.759	0.00031918	Down	0.855	0.802	0.846	1.116	1.07	1.11
Q6NZQ6	Zinc finger protein 740	Znf740	0.655	0.011492346	Down	0.972	0.771	0.761	1.177	1.413	1.234
O09114	Prostaglandin-H2 D-isomerase	Ptgds	1.767	0.000198686	Up	1.342	1.37	1.439	0.841	0.738	0.77
O88851	Putative hydrolase	Rbbp9	1.323	7.61942E-05	Up	1.038	1.07	1.057	0.78	0.793	0.819
Q6P6M7	O-phosphoseryl-tRNA(Sec) selenium transferase	Sepsecs	1.418	0.000640623	Up	1.306	1.276	1.224	0.902	0.844	0.938
Q80X82	Symplekin	Sympk	0.757	0.002321862	Down	0.814	0.92	0.911	1.142	1.169	1.181
Q5NBU8	XIAP-associated factor 1	Xaf1	0.528	0.000238339	Down	0.835	0.749	0.736	1.425	1.407	1.561
Q8VE97	Serine/arginine-rich splicing factor 4	Srsf4	0.646	0.000334142	Down	0.823	0.777	0.75	1.271	1.207	1.158
Q8BL97	Serine/arginine-rich splicing factor 7	Srsf7	0.727	0.000158069	Down	0.869	0.835	0.886	1.178	1.222	1.162
Q9D883	Splicing factor U2AF 35 kDa subunit	U2af1	0.651	0.002387563	Down	0.836	0.869	0.74	1.156	1.32	1.279
Q5H8C4	Vacuolar protein sorting-associated	Vps13a	0.752	0.005856704	Down	0.812	0.975	0.891	1.21	1.168	1.185
Q921F2	TAR DNA-binding protein 43	Tardbp	0.677	0.000108619	Down	0.845	0.804	0.799	1.236	1.216	1.162
Q8K284	General transcription factor 3C polypeptide 1	Gtf3c1	0.708	0.001648671	Down	0.796	0.879	0.803	1.216	1.092	1.19
P04202	Transforming growth factor beta-1 proprotein	Tgfb1	0.441	0.000888744	Down	0.779	0.584	0.662	1.632	1.418	1.546
Q9DBU0	Transmembrane 9 superfamily member 1	Tm9sf1	1.337	0.004830814	Up	1.24	1.07	1.145	0.91	0.846	0.829
P52196	Thiosulfate	Tst	1.397	0.00043405	Up	1.167	1.157	1.26	0.829	0.872	0.864
Q9R233	Tapasin	Tapbp	0.63	2.63298E-05	Down	0.881	0.927	0.864	1.409	1.423	1.409
P08043	Zinc finger protein 2	Zfp2	0.415	0.000680457	Down	0.726	0.554	0.576	1.476	1.6	1.398
Q60790	Ras GTPase-activating protein 3	Rasa3	0.66	0.008468858	Down	0.738	0.785	0.969	1.33	1.243	1.205
Q9D0B0	Serine/arginine-rich splicing factor 9	Srsf9	0.703	0.001642192	Down	0.89	0.77	0.809	1.14	1.156	1.216
Q9Z0H1	WD repeat-containing protein 46	Wdr46	0.688	0.01799811	Down	1.041	0.804	0.909	1.269	1.503	1.231
Q6NZF1	Zinc finger CCCH domain-containing	Zc3h11a	0.722	0.001160594	Down	0.84	0.919	0.824	1.173	1.243	1.164
Q02526	Zinc finger protein 41	Zfp41	0.42	0.028227643	Down	0.828	0.361	0.831	1.661	1.554	1.59
Q9R1B9	Slit homolog 2 protein	Slit2	1.357	0.007179114	Up	1.164	1.336	1.299	1.004	0.864	0.931
P35710	Transcription factor	Sox5	0.7	0.013477133	Down	0.774	0.868	0.888	1.337	1.231	1.046
Q922F4	Tubulin beta-6 chain	Tubb6	0.71	0.012636721	Down	0.7	0.797	0.721	1.003	1.182	0.939
Q8BWT1	3-ketoacyl-CoA thiolase, mitochondrial	Acaa2	1.383	1.64276E-05	Up	1.236	1.267	1.254	0.891	0.901	0.925
Q8CD92	Tetratricopeptide repeat protein 27	Ttc27	1.581	0.000959925	Up	1.3	1.298	1.192	0.735	0.856	0.806
Q8VHL0	Urea transporter 1	Slc14a1	0.569	7.49912E-07	Down	0.812	0.842	0.83	1.46	1.455	1.454
O89090	Transcription factor Sp1	Sp1	0.767	0.016246539	Down	0.972	0.774	0.878	1.16	1.152	1.108
Q8QZT1	Acetyl-CoA acetyltransferase, mitochondrial	Acat1	1.378	1.56247E-05	Up	1.25	1.196	1.23	0.889	0.888	0.89
P20152	Vimentin	Vim	0.736	4.38354E-05	Down	0.823	0.83	0.79	1.11	1.096	1.114
Q8BL74	General transcription factor 3C polypeptide 2	Gtf3c2	0.572	0.00045148	Down	0.856	0.759	0.722	1.404	1.348	1.332
Q5U4D9	THO complex subunit 6 homolog	Thoc6	0.714	0.002105635	Down	0.844	0.853	0.737	1.156	1.127	1.125
Q3UUI3	Acyl-coenzyme A thioesterase THEM4	Them4	1.404	0.000304322	Up	1.291	1.26	1.232	0.927	0.914	0.853
O70404	Vesicle-associated membrane protein 8	Vamp8	0.737	0.010489727	Down	0.887	0.725	0.873	1.167	1.084	1.123
P54227	Stathmin	Stmn1	0.723	0.01193334	Down	0.918	0.709	0.794	1.096	1.135	1.118
Q9DBY1	E3 ubiquitin-protein ligase synoviolin	Syvn1	1.359	0.032004436	Up	0.96	1.158	1.192	0.916	0.789	0.73
Q91ZE0	Trimethyllysine dioxygenase,	Tmlhe	1.459	0.002667365	Up	1.264	1.176	1.115	0.774	0.778	0.885
Q91WM3	U3 small nucleolar RNA-interacting protein	Rrp9	0.759	0.015836628	Down	0.889	0.915	0.912	1.358	1.114	1.108
Q60932	Voltage-dependent anion-selective channel	Vdac1	1.742	2.75239E-06	Up	1.409	1.398	1.462	0.814	0.824	0.813
P17141	Zinc finger protein 37	Zfp37	0.698	0.038253679	Down	1.054	0.691	0.872	1.224	1.246	1.277
P11031	Activated RNA polymerase II transcriptional	Sub1	0.655	0.001569106	Down	0.758	0.801	0.75	1.08	1.159	1.288

P56501	Mitochondrial uncoupling protein 3	Ucp3	2.004	1.13465E-05	Up	1.483	1.565	1.485	0.77	0.766	0.726
Q9QZM0	Ubiquilin-2	Ubqln2	0.671	0.026911126	Down	0.854	0.636	0.934	1.262	1.222	1.131
Q8BJH1	Zinc finger C2HC domain-containing	Zc2hc1a	0.708	0.006186825	Down	0.984	0.792	0.935	1.277	1.255	1.296
Q9CWZ3	RNA-binding protein 8A	Rbm8a	0.678	0.005016002	Down	0.922	0.845	0.759	1.3	1.283	1.142
Q8CBA2	Schlafen family member	Slfn5	0.749	7.55335E-05	Down	0.905	0.88	0.867	1.197	1.153	1.193
P16254	Signal recognition particle 14 kDa protein	Srp14	0.666	0.000182792	Down	0.787	0.827	0.749	1.206	1.178	1.163
Q923B6	Metalloreductase	Steap4	1.53	0.001699199	Up	1.062	1.136	1.17	0.762	0.776	0.664
Q8BYC6	Serine/threonine-protein kinase TAO3	Taok3	0.588	0.022768225	Down	0.87	0.814	0.931	1.486	1.823	1.135
P68368	Tubulin alpha-4A chain	Tuba4a	0.632	0.001961007	Down	0.93	0.783	0.875	1.272	1.365	1.456
P25976	Nucleolar transcription factor 1	Ubtf	0.392	8.21219E-06	Down	0.68	0.613	0.64	1.682	1.624	1.628
Q91VW9	Zinc finger protein with KRAB and SCAN domains 3	Zkscan3	0.626	0.035811965	Down	0.891	0.582	0.848	1.191	1.098	1.417
Q9CPQ3	Mitochondrial import receptor subunit TOM22 homolog	Tomm22	1.524	0.004175343	Up	1.31	1.302	1.374	0.759	0.964	0.892
Q3TMP8	Trimeric intracellular cation channel type A	Tmem38	1.48	0.001302231	Up	1.5	1.336	1.485	0.982	0.916	1.021
A2AJB2	Transmembrane protein 141	Tmem14	1.526	9.47056E-05	Up	1.283	1.322	1.384	0.886	0.883	0.845
B2RVY9	Transmembrane protein 182	Tmem18	1.512	0.000219257	Up	1.138	1.212	1.104	0.75	0.788	0.747
P25446	Tumor necrosis factor receptor superfamily member 6	Fas	1.446	0.000732799	Up	1.18	1.205	1.189	0.763	0.871	0.837
Q9D735	Telomerase RNA component interacting RNase	Trir	0.656	0.0018904	Down	0.995	0.969	0.837	1.476	1.425	1.367
Q8VBT1	Beta-taxilin	Txlnb	1.328	0.005211736	Up	1.093	1.164	1.063	0.762	0.874	0.864
Q8K1B8	Fermitin family homolog	Fermt3	0.315	0.000136639	Down	0.601	0.593	0.469	1.773	1.734	1.769
Q99NB8	Ubiquilin-4	Ubqln4	0.704	0.011428694	Down	0.77	0.874	0.868	1.251	1.036	1.279
Q8K0L9	Zinc finger and BTB domain-containing protein 20	Zbtb20	0.56	2.41242E-05	Down	0.756	0.793	0.727	1.362	1.366	1.336
Q62523	Zyxin	Zyx	0.749	8.7445E-05	Down	0.922	0.903	0.881	1.213	1.223	1.175
Q8C0L0	Thioredoxin-related transmembrane protein 4	Tmx4	1.301	0.004116814	Up	1.16	1.204	1.192	0.892	0.988	0.853
Q8BMD6	Transmembrane protein 260	Tmem26	1.324	0.031809097	Up	1.117	0.996	1.29	0.825	0.816	0.929
Q6ZPZ3	Zinc finger CCCH domain-containing	Zc3h4	0.435	0.000198905	Down	0.731	0.754	0.703	1.484	1.74	1.806
Q9ERA6	Tuftelin-interacting protein 11	Tfip11	0.594	0.000554352	Down	0.84	0.832	0.72	1.366	1.302	1.357
Q8BZZ3	NEDD4-like E3 ubiquitin-protein ligase	Wwp1	1.407	0.041123506	Up	1.002	1.324	1.344	0.768	0.93	0.911
P36371	Antigen peptide transporter 2	Tap2	0.548	0.000153621	Down	0.894	0.868	0.783	1.592	1.543	1.507
Q5F293	Zinc finger and BTB domain-containing	Zbtb4	0.528	0.001954299	Down	0.882	0.722	0.696	1.356	1.599	1.405
Q60596	DNA repair protein XRCC1	Xrcc1	0.745	0.005497641	Down	0.908	0.78	0.791	1.128	1.142	1.056
Q8BI66	Zinc finger protein 526	Znf526	0.769	0.024488001	Down	1.038	0.895	0.888	1.114	1.21	1.346
Q62293	T-cell-specific guanine nucleotide triphosphate-binding protein 1	Tgtp1	0.604	0.000374913	Down	0.922	0.831	0.833	1.458	1.343	1.477
Q9D710	Thioredoxin-related transmembrane protein 2	Tmx2	1.352	0.002266976	Up	1.233	1.141	1.177	0.938	0.863	0.826
Q8CC21	Tetratricopeptide repeat protein 19,	Ttc19	1.819	0.000253041	Up	1.393	1.441	1.407	0.72	0.763	0.848
Q60931	Voltage-dependent anion-selective channel	Vdac3	1.523	7.51741E-06	Up	1.346	1.309	1.35	0.888	0.859	0.882
P35918	Vascular endothelial growth factor receptor 2	Kdr	1.379	0.000236166	Up	1.183	1.176	1.224	0.87	0.831	0.898
Q3UX10	Tubulin alpha chain-like	Tubal3	0.686	0.008538309	Down	0.941	0.859	0.741	1.166	1.323	1.215
Q9JMA2	Queuine tRNA-ribosyltransferase catalytic subunit 1	Qtrt1	1.806	0.008056271	Up	1.326	1.123	1.126	0.811	0.576	0.593
E9Q9K5	Triadin	Trdn	2.359	0.000399641	Up	1.689	1.56	1.476	0.643	0.758	0.602
Q9R0Q1	Synaptotagmin-like protein 4	Sytl4	0.471	0.000542269	Down	0.76	0.77	0.622	1.475	1.614	1.482
Q80X50	Ubiquitin-associated protein 2-like	Ubap2l	0.682	0.003747094	Down	0.912	0.935	0.82	1.346	1.384	1.182

Q5ND56	TLC domain-containing protein 3A	Tlcd3a	0.73	0.018258849	Down	0.992	0.786	0.948	1.22	1.346	1.169
Q3UPF5	Zinc finger CCCH-type antiviral protein 1	Zc3hav1	0.757	0.005409762	Down	0.845	0.825	0.939	1.182	1.188	1.077
Q69Z99	Zinc finger protein 512	Znf512	0.635	0.000209259	Down	0.887	0.878	0.836	1.438	1.36	1.296
P14115	60S ribosomal protein L27a	Rpl27a	0.731	0.00024156	Down	0.889	0.877	0.869	1.196	1.255	1.154
Q6P5D8	Structural maintenance of chromosomes flexible hinge domain-	Smchd1	0.729	7.46196E-05	Down	0.865	0.859	0.819	1.147	1.164	1.179
P26369	Splicing factor U2AF 65 kDa subunit	U2af2	0.658	0.000107135	Down	0.802	0.774	0.798	1.22	1.244	1.145
Q9JKB3	Y-box-binding protein 3	Ybx3	0.673	0.000609236	Down	0.832	0.843	0.806	1.312	1.15	1.225
Q64669	NAD(P)H dehydrogenase	Nqo1	1.327	0.001134667	Up	1.106	1.159	1.116	0.798	0.87	0.88
Q61469	Phospholipid	Plpp1	1.538	0.003268312	Up	1.024	1.271	1.173	0.756	0.717	0.782
O88492	Perilipin-4	Plin4	1.671	9.63539E-06	Up	1.154	1.18	1.152	0.694	0.676	0.716
Q8VE37	Regulator of chromosome	Rcc1	0.758	0.000766418	Down	0.972	0.879	0.908	1.226	1.204	1.21
Q6ZWY3	40S ribosomal protein S27-like	Rps27l	0.58	0.001187708	Down	0.753	0.818	0.652	1.29	1.286	1.258
Q80YR5	Scaffold attachment factor B2	Safb2	0.696	0.000938207	Down	0.883	0.853	0.818	1.262	1.27	1.14
Q61234	Alpha-1-syntrophin	Snta1	1.498	0.000271656	Up	1.021	1.143	1.078	0.718	0.732	0.714
Q60930	Voltage-dependent anion-selective channel	Vdac2	1.509	7.30689E-08	Up	1.318	1.318	1.308	0.869	0.867	0.877
Q9R020	Zinc finger Ran-binding domain-containing	Zranb2	0.557	0.00411454	Down	0.712	0.771	0.839	1.266	1.638	1.265
Q9DB26	Phytanoyl-CoA dioxygenase domain-containing protein 1	Phyhd1	1.81	0.046930053	Up	1.103	1.756	0.915	0.659	0.708	0.718
P62267	40S ribosomal protein	Rps23	0.663	0.000576639	Down	0.842	0.763	0.861	1.267	1.26	1.195
Q9D1J3	SAP domain-containing ribonucleoprotein	Sarnp	0.594	0.00021862	Down	0.843	0.761	0.861	1.35	1.379	1.422
O88974	Histone-lysine N-methyltransferase	Setdb1	0.763	0.004317886	Down	0.948	0.857	0.897	1.197	1.101	1.244
Q64674	Spermidine synthase	Srm	0.735	0.001000499	Down	0.834	0.796	0.85	1.192	1.09	1.09
P07759	Serine protease inhibitor A3K	Serpina3	0.516	0.000482122	Down	0.753	0.89	0.724	1.548	1.502	1.534
Q8BGG7	Ubiquitin-associated and SH3 domain-containing protein B	Ubash3b	0.422	0.00016805	Down	0.577	0.704	0.654	1.505	1.472	1.603
O35892	Nuclear autoantigen Sp-100	Sp100	0.319	2.16067E-05	Down	0.619	0.601	0.529	1.781	1.832	1.863
P39876	Metalloproteinase inhibitor 3	Timp3	0.449	0.00086696	Down	0.831	0.615	0.68	1.595	1.521	1.617
Q9QZU9	Ubiquitin/ISG15-conjugating enzyme E2	Ube2l6	0.661	0.006524877	Down	0.838	0.822	0.886	1.118	1.281	1.451
Q9CR11	YEATS domain-containing protein 4	Yeats4	0.655	0.015124337	Down	0.935	0.652	0.832	1.243	1.217	1.232
Q8R143	Pituitary tumor-transforming gene 1 protein-interacting	Pttg1ip	0.72	6.47407E-05	Down	0.962	0.933	0.901	1.296	1.295	1.292
Q9R0U0	Serine/arginine-rich splicing factor 10	Srsf10	0.755	0.012477467	Down	0.956	0.775	0.931	1.189	1.17	1.169
Q08943	FACT complex subunit SSRP1	Ssrp1	0.493	1.56858E-06	Down	0.694	0.682	0.689	1.4	1.431	1.358
P40630	Transcription factor A, mitochondrial	Tfam	0.272	1.80865E-05	Down	0.584	0.553	0.494	2.009	2.07	1.912
Q04750	DNA topoisomerase 1	Top1	0.594	0.000230532	Down	0.864	0.863	0.764	1.376	1.41	1.409
Q80YV3	Transformation/transcription domain-associated	Trrap	0.748	0.021238806	Down	0.753	0.989	0.835	1.13	1.178	1.136
Q3UDK1	TRAF-type zinc finger domain-containing	Trafd1	0.541	0.000693089	Down	0.691	0.76	0.774	1.489	1.236	1.386
Q00899	Transcriptional repressor protein YY1	Yy1	0.754	2.40663E-05	Down	0.902	0.875	0.883	1.157	1.193	1.18
P62281	40S ribosomal protein	Rps11	0.733	0.000907286	Down	0.832	0.867	0.802	1.13	1.194	1.088
P97351	40S ribosomal protein	Rps3a	0.606	2.82047E-05	Down	0.812	0.755	0.78	1.266	1.296	1.311
P63276	40S ribosomal protein	Rps17	0.761	0.002603002	Down	0.933	0.822	0.853	1.174	1.11	1.141
Q91WA6	Sharpin	Sharpin	0.689	0.000767416	Down	0.859	0.805	0.871	1.178	1.305	1.194
Q8C3Q5	Protein shisa-7	Shisa7	1.376	0.045003781	Up	1.264	1.068	1.416	1.035	0.802	0.886
O89032	SH3 and PX domain-containing protein 2A	Sh3pxd2	1.353	0.000336299	Up	1.059	0.98	1.055	0.763	0.772	0.751
Q91WP6	Serine protease inhibitor A3N	Serpina3	1.809	0.000323742	Up	1.129	1.174	1.267	0.61	0.695	0.669
Q8R3N6	THO complex subunit 1	Thoc1	0.759	0.007251132	Down	0.93	0.805	0.922	1.218	1.18	1.104
Q8BK08	Transmembrane protein 11, mitochondrial	Tmem11	1.627	1.52625E-05	Up	1.301	1.345	1.383	0.836	0.828	0.813

Q91YR7	Pre-mRNA-processing factor 6	Prpf6	0.758	0.000798145	Down	0.87	0.897	0.822	1.174	1.11	1.132
Q9CQS4	Solute carrier family 25 member 46	Slc25a46	1.45	0.001711855	Up	1.228	1.323	1.333	0.902	0.953	0.824
O55128	Histone deacetylase complex subunit SAP18	Sap18	0.721	0.000899486	Down	0.832	0.898	0.891	1.255	1.233	1.146
P70460	Vasodilator-stimulated phosphoprotein	Vasp	0.736	1.48336E-05	Down	0.893	0.89	0.863	1.2	1.21	1.187
Q923D5	WW domain-binding protein 11	Wbp11	0.766	0.018858819	Down	0.814	1.028	0.864	1.17	1.156	1.208
P52760	2-iminobutanoate/2-iminopropanoate	Rida	0.62	0.000639705	Down	0.798	0.811	0.901	1.42	1.358	1.273
P62843	40S ribosomal protein	Rps15	0.657	0.000969226	Down	0.803	0.868	0.771	1.236	1.169	1.314
P31532	Serum amyloid A-4	Saa4	1.418	0.001097394	Up	1.265	1.312	1.26	0.847	0.97	0.888
Q7TQ48	Sarcalumenin	Srl	2.218	8.16025E-06	Up	1.573	1.52	1.641	0.693	0.732	0.709
P42225	Signal transducer and activator of transcription	Stat1	0.544	5.75517E-05	Down	0.776	0.819	0.775	1.495	1.489	1.369
Q921N7	Transmembrane protein 70, mitochondrial	Tmem70	1.377	0.00314259	Up	1.085	1.248	1.27	0.874	0.877	0.865
Q8JZL7	Ras-GEF domain-containing family member 1B	Rasgef1b	1.414	0.001443654	Up	1.303	1.211	1.311	0.838	0.925	0.942
P62245	40S ribosomal protein S15a	Rps15a	0.714	0.000239162	Down	0.857	0.857	0.808	1.135	1.206	1.193
Q9CZX8	40S ribosomal protein	Rps19	0.706	0.0006349	Down	0.796	0.854	0.772	1.19	1.12	1.121
P62082	40S ribosomal protein	Rps7	0.674	0.001244898	Down	0.822	0.897	0.831	1.319	1.301	1.163
Q80T69	Lysine-specific demethylase 9	Rsbn1	3.886	2.43614E-06	Up	2.187	1.978	2.037	0.521	0.55	0.525
Q9WVL2	Signal transducer and activator of transcription	Stat2	0.595	0.000357803	Down	0.891	0.82	0.798	1.481	1.411	1.325
Q8CI59	Metalloreductase	Steap3	0.763	0.005077656	Down	0.856	0.939	0.909	1.184	1.098	1.261
Q6IRU2	Tropomyosin alpha-4	Tpm4	0.686	7.74965E-06	Down	0.827	0.818	0.799	1.205	1.174	1.184
P13439	Uridine 5'-monophosphate synthase	Umps	0.732	0.017712737	Down	0.709	0.834	0.871	1.173	0.993	1.13
P30355	Arachidonate 5-lipoxygenase-activating protein	Alox5ap	1.319	0.031604527	Up	1.162	1.24	1.142	0.769	0.889	1.028
P34928	Apolipoprotein C-I	Apoc1	1.727	0.000746277	Up	0.863	1.05	0.991	0.566	0.56	0.556
Q91YY4	ATP synthase mitochondrial F1 complex assembly factor	Atpaf2	1.661	0.000107085	Up	1.324	1.351	1.291	0.829	0.809	0.749
E9PV24	Fibrinogen alpha chain	Fga	0.488	5.79859E-05	Down	0.802	0.722	0.734	1.602	1.477	1.55
P50149	Guanine nucleotide-binding protein G(t)	Gnat2	5.93	0.000149407	Up	1.642	1.496	1.778	0.216	0.314	0.299

Supplementary Table 3. Differentially expressed proteins in CMECs between GCGR mAb- and IgG control (Ctrl)-treated *db/db* mice

Protein accession	Protein description	Gene name	Ratio (mAb vs. Ctrl)	P value (mAb vs. Ctrl)	Regulated type	mAb #1	mAb #2	mAb #3	*Ctrl #1*	*Ctrl #2*	*Ctrl #3*
Q99KI0	Aconitate hydratase, mitochondrial	Aco2	0.698	1.80444E-08	Down	0.873	0.874	0.879	1.254	1.251	1.258
E9Q401	Ryanodine receptor 2	Ryr2	0.538	4.93278E-08	Down	0.766	0.78	0.768	1.436	1.428	1.434
P41216	Long-chain-fatty-acid--CoA ligase 1	Acsl1	0.563	1.09467E-07	Down	0.815	0.824	0.806	1.453	1.449	1.443
P51667	Myosin regulatory light chain 2, ventricular/cardiac	Myl2	0.523	2.06178E-07	Down	0.636	0.638	0.627	1.229	1.202	1.205
Q62425	Cytochrome c oxidase subunit NDUFA4	Ndufa4	0.646	2.35527E-07	Down	0.868	0.879	0.867	1.338	1.349	1.358
Q61598	Rab GDP dissociation inhibitor beta	Gdi2	1.338	2.6306E-07	Up	1.185	1.189	1.191	0.895	0.884	0.885
P16110	Galectin-3	Lgals3	2.175	2.79511E-07	Up	1.534	1.514	1.555	0.694	0.708	0.714
P56480	ATP synthase subunit beta, mitochondrial	Atp5f1b	0.754	3.06568E-07	Down	0.884	0.888	0.887	1.168	1.173	1.184
O70468	Myosin-binding protein C, cardiac-type	Mybpc3	0.562	4.75473E-07	Down	0.714	0.716	0.702	1.27	1.246	1.277
A2ASS6	Titin	Ttn	0.536	5.91088E-07	Down	0.689	0.688	0.668	1.282	1.262	1.272
Q91YP0	L-2-hydroxyglutarate dehydrogenase, mitochondrial	L2hgdh	0.723	6.60921E-07	Down	0.853	0.858	0.867	1.197	1.185	1.184
Q61646	Haptoglobin	Hp	0.435	7.59156E-07	Down	0.676	0.701	0.672	1.596	1.558	1.554
P42125	Enoyl-CoA delta isomerase 1,	Eci1	0.736	8.99335E-07	Down	0.912	0.902	0.914	1.236	1.245	1.226
Q9D8U8	Sorting nexin-5	Snx5	1.306	1.12347E-06	Up	1.158	1.162	1.142	0.886	0.881	0.884
Q9R0H0	Peroxisomal acyl-coenzyme A oxidase 1	Acox1	0.581	1.13165E-06	Down	0.812	0.833	0.838	1.41	1.428	1.439
Q9WUR2	Enoyl-CoA delta isomerase 2,	Eci2	0.678	1.19013E-06	Down	0.917	0.911	0.9	1.325	1.349	1.35
Q62234	Myomesin-1	Myom1	0.493	1.27809E-06	Down	0.701	0.694	0.698	1.412	1.379	1.452
O09161	Calsequestrin-2	Casq2	0.556	1.28278E-06	Down	0.84	0.823	0.811	1.461	1.49	1.497
Q9CRB9	MICOS complex subunit Mic19	Chchd3	0.573	1.61452E-06	Down	0.834	0.824	0.809	1.411	1.436	1.456
Q9CXJ4	Mitochondrial potassium channel ATP-binding	Abcb8	0.504	1.70613E-06	Down	0.8	0.813	0.826	1.578	1.614	1.65
Q921G7	Electron transfer flavoprotein-ubiquinone oxidoreductase, mitochondrial	Etfdh	0.714	1.83843E-06	Down	0.892	0.883	0.874	1.224	1.24	1.246
Q8K2B3	Succinate dehydrogenase [ubiquinone] flavoprotein subunit,	Sdha	0.735	2.07337E-06	Down	0.897	0.906	0.884	1.222	1.219	1.213
Q6P8J7	Creatine kinase S-type, mitochondrial	Ckmt2	0.596	2.10584E-06	Down	0.861	0.867	0.845	1.426	1.425	1.469
Q60931	Voltage-dependent anion-selective channel	Vdac3	0.621	2.14261E-06	Down	0.819	0.835	0.834	1.346	1.309	1.35
P05202	Aspartate aminotransferase,	Got2	0.727	2.44902E-06	Down	0.907	0.889	0.892	1.24	1.22	1.238
Q9EPL9	Peroxisomal acyl-coenzyme A oxidase 3	Acox3	0.699	2.61316E-06	Down	0.99	0.998	0.992	1.408	1.408	1.446
Q9DB77	Cytochrome b-c1 complex subunit 2,	Uqcrc2	0.717	2.74809E-06	Down	0.882	0.883	0.863	1.211	1.224	1.229
Q02566	Myosin-6	Myh6	0.538	3.17295E-06	Down	0.637	0.656	0.632	1.216	1.166	1.199
P48962	ADP/ATP translocase 1	Slc25a4	0.729	3.3244E-06	Down	0.894	0.879	0.87	1.201	1.214	1.213
Q78IK4	MICOS complex subunit Mic27	Apool	0.641	4.11309E-06	Down	0.863	0.828	0.845	1.325	1.323	1.306
P21440	Phosphatidylcholine translocator ABCB4	Abcb4	0.44	4.46982E-06	Down	0.61	0.645	0.602	1.409	1.378	1.435
E9Q557	Desmoplakin	Dsp	0.546	4.60262E-06	Down	0.791	0.817	0.78	1.492	1.445	1.438
O35857	Mitochondrial import inner membrane translocase subunit	Timm44	0.724	4.78327E-06	Down	0.866	0.867	0.863	1.174	1.199	1.213
Q8BWF0	Succinate-semialdehyde dehydrogenase, mitochondrial	Aldh5a1	0.689	4.8591E-06	Down	0.868	0.869	0.843	1.258	1.236	1.249
Q9JJW5	Myozenin-2	Myoz2	0.621	4.9043E-06	Down	0.792	0.804	0.83	1.292	1.306	1.308
P50544	Very long-chain specific acyl-CoA dehydrogenase, mitochondrial	Acadvl	0.684	4.98281E-06	Down	0.907	0.92	0.899	1.336	1.305	1.346

Q80T69	Lysine-specific demethylase 9	Rsbn1	0.245	5.26855E-06	Down	0.53	0.513	0.477	2.187	1.978	2.037
P47934	Carnitine O-acetyltransferase	Crat	0.748	5.31964E-06	Down	0.933	0.923	0.915	1.24	1.218	1.246
Q91ZA3	Propionyl-CoA carboxylase alpha chain,	Pcca	0.768	5.54713E-06	Down	0.893	0.898	0.893	1.184	1.155	1.157
P19783	Cytochrome c oxidase subunit 4 isoform 1, mitochondrial	Cox4i1	0.731	5.66682E-06	Down	0.885	0.893	0.865	1.199	1.208	1.207
Q8BWT1	3-ketoacyl-CoA thiolase, mitochondrial	Acaa2	0.697	5.67276E-06	Down	0.879	0.882	0.858	1.236	1.267	1.254
P19123	Troponin C, slow skeletal and cardiac muscles	Tnnc1	0.497	5.98419E-06	Down	0.623	0.64	0.659	1.31	1.25	1.305
P19536	Cytochrome c oxidase subunit 5B,	Cox5b	0.672	6.17478E-06	Down	0.844	0.838	0.84	1.239	1.283	1.233
P61014	Cardiac phospholamban	Pln	0.493	6.39821E-06	Down	0.675	0.721	0.674	1.388	1.398	1.412
Q9CZU6	Citrate synthase, mitochondrial	Cs	0.725	6.99651E-06	Down	0.916	0.901	0.888	1.23	1.253	1.248
Q9CR68	Cytochrome b-c1 complex subunit Rieske, mitochondrial	Uqcrfs1	0.672	7.06229E-06	Down	0.837	0.831	0.843	1.254	1.266	1.215
Q99LC5	Electron transfer flavoprotein subunit alpha, mitochondrial	Etfa	0.68	7.80301E-06	Down	0.87	0.863	0.835	1.255	1.268	1.254
P09542	Myosin light chain 3	Myl3	0.543	7.9673E-06	Down	0.619	0.653	0.641	1.2	1.145	1.177
P45952	Medium-chain specific acyl-CoA dehydrogenase, mitochondrial	Acadm	0.713	8.50471E-06	Down	0.928	0.921	0.921	1.265	1.309	1.309
P54071	Isocitrate dehydrogenase [NADP], mitochondrial	Idh2	0.749	9.25445E-06	Down	0.958	0.941	0.933	1.263	1.244	1.272
Q924X2	Carnitine O-palmitoyltransferase 1, muscle isoform	Cpt1b	0.521	9.373E-06	Down	0.81	0.788	0.782	1.518	1.473	1.581
Q7TQ48	Sarcalumenin	Srl	0.504	9.45429E-06	Down	0.793	0.811	0.784	1.573	1.52	1.641
P00184	Cytochrome P450 1A1	Cyp1a1	0.326	9.48067E-06	Down	0.533	0.51	0.476	1.53	1.508	1.623
P01942	Hemoglobin subunit alpha	Hba	1.316	9.71803E-06	Up	1.16	1.124	1.132	0.863	0.869	0.863
Q6P5H2	Nestin	Nes	1.401	1.0017E-05	Up	1.066	1.054	1.086	0.773	0.765	0.751
Q9DCT2	NADH dehydrogenase [ubiquinone] iron-sulfur protein 3, mitochondrial	Ndufs3	0.733	1.02051E-05	Down	0.863	0.893	0.866	1.198	1.194	1.186
Q60930	Voltage-dependent anion-selective channel	Vdac2	0.646	1.03836E-05	Down	0.872	0.851	0.826	1.318	1.318	1.308
P07724	Serum albumin	Alb	1.384	1.06443E-05	Up	1.336	1.288	1.296	0.944	0.938	0.95
P63038	60 kDa heat shock protein, mitochondrial	Hspd1	0.75	1.11952E-05	Down	0.858	0.84	0.837	1.136	1.133	1.11
P62897	Cytochrome c, somatic	Cycs	0.533	1.14615E-05	Down	0.818	0.776	0.758	1.49	1.461	1.461
Q8VCT4	Carboxylesterase 1D	Ces1d	0.39	1.16666E-05	Down	0.615	0.635	0.629	1.63	1.683	1.502
Q60932	Voltage-dependent anion-selective channel	Vdac1	0.575	1.1755E-05	Down	0.84	0.817	0.796	1.409	1.398	1.462
O55143	Sarcoplasmic/endoplasmic reticulum calcium	Atp2a2	0.504	1.17724E-05	Down	0.734	0.75	0.705	1.444	1.402	1.493
Q8BXV2	BRI3-binding protein	Bri3bp	0.447	1.21901E-05	Down	0.712	0.69	0.652	1.528	1.488	1.574
Q8CAQ8	MICOS complex subunit Mic60	Immt	0.625	1.22063E-05	Down	0.863	0.859	0.828	1.383	1.329	1.371
Q91VD9	NADH-ubiquinone oxidoreductase 75 kDa subunit, mitochondrial	Ndufs1	0.769	1.3197E-05	Down	0.917	0.909	0.89	1.181	1.185	1.166
Q8BH95	Enoyl-CoA hydratase, mitochondrial	Echs1	0.706	1.36265E-05	Down	0.884	0.883	0.872	1.275	1.22	1.242
Q8R1I1	Cytochrome b-c1 complex subunit 9	Uqcr10	0.717	1.36461E-05	Down	0.87	0.872	0.858	1.208	1.185	1.235
Q3UU35	Ovostatin homolog	Ovos	0.616	1.39384E-05	Down	0.81	0.788	0.768	1.288	1.254	1.302
P00493	Hypoxanthine-guanine phosphoribosyltransferas	Hprt1	1.56	1.59227E-05	Up	1.299	1.226	1.249	0.807	0.814	0.798
Q80XL6	Acyl-CoA dehydrogenase family member 11	Acad11	0.532	1.64892E-05	Down	0.713	0.725	0.707	1.374	1.278	1.378
P58281	Dynamin-like 120 kDa protein, mitochondrial	Opa1	0.653	1.65485E-05	Down	0.885	0.868	0.859	1.357	1.293	1.347
P20108	Thioredoxin-dependent peroxide reductase, mitochondrial	Prdx3	0.743	1.66608E-05	Down	0.877	0.893	0.866	1.183	1.2	1.166
P08249	Malate dehydrogenase, mitochondrial	Mdh2	0.72	1.74899E-05	Down	0.897	0.894	0.861	1.224	1.222	1.236
P09528	Ferritin heavy chain	Fth1	4.032	1.84966E-05	Up	1.87	1.87	1.969	0.44	0.526	0.45

Q9CRB8	Mitochondrial fission process protein 1	Mtfp1	0.682	1.85221E-05	Down	0.812	0.841	0.814	1.215	1.224	1.179
P49813	Tropomodulin-1	Tmod1	0.53	1.89194E-05	Down	0.671	0.692	0.636	1.283	1.242	1.244
Q8R0Y6	Cytosolic 10-formyltetrahydrofolate dehydrogenase	Aldh1l1	1.422	1.93028E-05	Up	1.098	1.123	1.097	0.79	0.758	0.785
Q8BRH0	Protein O-mannosyl-transferase TMTC3	Tmtc3	0.746	1.96199E-05	Down	0.92	0.924	0.924	1.21	1.235	1.263
Q9D855	Cytochrome b-c1 complex subunit 7	Uqcrb	0.743	2.0714E-05	Down	0.906	0.901	0.912	1.213	1.249	1.198
O35459	Delta(3,5)-Delta(2,4)-dienoyl-CoA isomerase, mitochondrial	Ech1	0.623	2.1029E-05	Down	0.89	0.896	0.845	1.384	1.415	1.426
Q9DCD0	6-phosphogluconate dehydrogenase, decarboxylating	Pgd	1.372	2.11987E-05	Up	1.229	1.218	1.178	0.887	0.873	0.883
Q61941	NAD(P) transhydrogenase,	Nnt	0.746	2.13303E-05	Down	0.825	0.793	0.811	1.095	1.086	1.074
Q9CPP6	NADH dehydrogenase [ubiquinone] 1 alpha subcomplex subunit 5	Ndufa5	0.739	2.1408E-05	Down	0.883	0.914	0.904	1.238	1.215	1.203
Q8CAK1	Putative transferase CAF17 homolog,	Iba57	0.758	2.16524E-05	Down	0.873	0.864	0.868	1.167	1.121	1.15
Q00612	Glucose-6-phosphate 1-dehydrogenase X	G6pdx	1.334	2.20544E-05	Up	1.252	1.209	1.222	0.926	0.907	0.928
Q8BW75	Amine oxidase [flavin-containing] B	Maob	0.626	2.21184E-05	Down	0.823	0.856	0.877	1.362	1.338	1.38
Q8VEM8	Phosphate carrier protein, mitochondrial	Slc25a3	0.72	2.22926E-05	Down	0.878	0.859	0.847	1.184	1.22	1.184
Q8BMS4	Ubiquinone biosynthesis O-methyltransferase, mitochondrial	Coq3	0.669	2.35877E-05	Down	0.902	0.874	0.86	1.287	1.317	1.338
Q8BLF1	Neutral cholesterol ester hydrolase 1	Nceh1	0.602	2.39796E-05	Down	0.781	0.788	0.784	1.308	1.249	1.35
Q9D0M3	Cytochrome c1, heme protein, mitochondrial	Cyc1	0.747	2.4278E-05	Down	0.904	0.897	0.873	1.179	1.211	1.191
Q9D6R2	Isocitrate dehydrogenase [NAD] subunit alpha, mitochondrial	Idh3a	0.691	2.43337E-05	Down	0.833	0.867	0.853	1.261	1.22	1.214
Q6P6M5	Peroxisomal membrane protein 11C	Pex11g	0.669	2.48066E-05	Down	0.883	0.898	0.853	1.336	1.312	1.29
Q99LB2	Dehydrogenase/reductase SDR family member 4	Dhrs4	0.746	2.63226E-05	Down	0.912	0.896	0.895	1.207	1.182	1.232
P31532	Serum amyloid A-4 protein	Saa4	0.677	2.70051E-05	Down	0.887	0.85	0.861	1.265	1.312	1.26
Q99LC3	NADH dehydrogenase [ubiquinone] 1 alpha subcomplex subunit 10, mitochondrial	Ndufa10	0.739	2.72936E-05	Down	0.93	0.906	0.886	1.231	1.227	1.227
Q9JI91	Alpha-actinin-2	Actn2	0.762	2.73034E-05	Down	0.916	0.931	0.901	1.214	1.182	1.209
Q9JLT4	Thioredoxin reductase 2, mitochondrial	Txnrd2	0.706	2.77482E-05	Down	0.83	0.854	0.825	1.21	1.183	1.16
Q9QYR9	Acyl-coenzyme A thioesterase 2,	Acot2	0.693	2.77825E-05	Down	0.909	0.909	0.887	1.265	1.31	1.331
Q9D1H6	NADH dehydrogenase [ubiquinone] 1 alpha subcomplex assembly factor 4	Ndufaf4	0.674	2.82988E-05	Down	0.782	0.815	0.794	1.204	1.15	1.192
P58252	Elongation factor 2	Eef2	1.406	2.86061E-05	Up	1.191	1.152	1.16	0.839	0.842	0.81
Q9CQZ5	NADH dehydrogenase [ubiquinone] 1 alpha subcomplex subunit 6	Ndufa6	0.747	2.99643E-05	Down	0.884	0.876	0.893	1.172	1.166	1.214
Q91VH6	Protein MEMO1	Memo1	1.451	2.99921E-05	Up	1.187	1.195	1.186	0.847	0.814	0.798
A2AAJ9	Obscurin	Obscn	0.725	3.16747E-05	Down	0.876	0.876	0.858	1.17	1.227	1.205
P63323	40S ribosomal protein S12	Rps12	1.403	3.19275E-05	Up	1.166	1.165	1.183	0.812	0.857	0.835
Q3UN90	LYR motif-containing protein 9	Lyrm9	0.675	3.37339E-05	Down	0.834	0.802	0.843	1.242	1.196	1.236
Q9Z2Z6	Mitochondrial carnitine/acylcarnitine carrier protein	Slc25a20	0.708	3.41275E-05	Down	0.924	0.932	0.907	1.331	1.265	1.308
Q9DBL1	Short/branched chain specific acyl-CoA dehydrogenase, mitochondrial	Acadsb	0.764	3.41787E-05	Down	0.937	0.907	0.911	1.187	1.221	1.197
Q3TMP8	Trimeric intracellular cation channel type A	Tmem38a	0.448	3.42236E-05	Down	0.628	0.652	0.655	1.5	1.336	1.485

Q99MR9	Protein phosphatase 1 regulatory subunit 3A	Ppp1r3a	0.397	3.45355E-05	Down	0.559	0.494	0.548	1.403	1.289	1.341
Q61176	Arginase-1	Arg1	4.619	3.46264E-05	Up	2.055	1.985	2.219	0.395	0.497	0.463
Q61425	Hydroxyacyl-coenzyme A dehydrogenase, mitochondrial	Hadh	0.726	3.48455E-05	Down	0.914	0.916	0.876	1.232	1.237	1.257
Q9CQ75	NADH dehydrogenase [ubiquinone] 1 alpha subcomplex subunit 2	Ndufa2	0.747	3.5238E-05	Down	0.89	0.88	0.918	1.216	1.19	1.192
O88958	Glucosamine-6-phosphate isomerase 1	Gnpda1	1.411	3.58633E-05	Up	1.16	1.197	1.161	0.828	0.852	0.813
P00158	Cytochrome b	Mt-Cyb	0.711	3.62525E-05	Down	0.812	0.791	0.829	1.146	1.118	1.157
Q2TPA8	Hydroxysteroid dehydrogenase-like protein 2	Hsdl2	0.762	3.66962E-05	Down	0.949	0.938	0.911	1.215	1.217	1.238
Q8JZV7	N-acetylglucosamine-6-phosphate deacetylase	Amdhd2	1.332	3.67633E-05	Up	1.2	1.148	1.179	0.884	0.891	0.872
P58771	Tropomyosin alpha-1 chain	Tpm1	0.585	3.68883E-05	Down	0.737	0.702	0.675	1.189	1.225	1.202
Q8K031	StAR-related lipid transfer protein 8	Stard8	0.506	3.7086E-05	Down	0.659	0.595	0.625	1.24	1.272	1.199
Q8BGH2	Sorting and assembly machinery component 50 homolog	Samm50	0.671	3.99301E-05	Down	0.882	0.872	0.849	1.294	1.256	1.332
P97478	5-demethoxyubiquinone hydroxylase,	Coq7	0.643	3.99749E-05	Down	0.824	0.82	0.8	1.246	1.234	1.318
P51174	Long-chain specific acyl-CoA dehydrogenase, mitochondrial	Acadl	0.696	4.13836E-05	Down	0.906	0.878	0.857	1.243	1.284	1.265
O08749	Dihydrolipoyl dehydrogenase, mitochondrial	Dld	0.743	4.3663E-05	Down	0.875	0.871	0.847	1.17	1.138	1.184
Q9D1I5	Methylmalonyl-CoA epimerase, mitochondrial	Mcee	0.685	4.37626E-05	Down	0.835	0.798	0.822	1.163	1.223	1.199
Q8QZT1	Acetyl-CoA acetyltransferase, mitochondrial	Acat1	0.766	4.43786E-05	Down	0.948	0.931	0.936	1.25	1.196	1.23
P56391	Cytochrome c oxidase subunit 6B1	Cox6b1	0.637	4.45651E-05	Down	0.87	0.923	0.854	1.378	1.382	1.393
Q9DAN9	Uncharacterized protein CFAP97D1	Cfap97d1	0.53	4.5515E-05	Down	0.635	0.631	0.656	1.263	1.224	1.137
Q8BH59	Calcium-binding mitochondrial carrier protein Aralar1	Slc25a12	0.694	4.57237E-05	Down	0.871	0.871	0.824	1.229	1.224	1.247
Q9Z0X1	Apoptosis-inducing factor 1, mitochondrial	Aifm1	0.575	4.71217E-05	Down	0.828	0.846	0.827	1.402	1.416	1.534
Q03265	ATP synthase subunit alpha, mitochondrial	Atp5f1a	0.744	4.73213E-05	Down	0.882	0.873	0.864	1.192	1.14	1.19
Q9D6M3	Mitochondrial glutamate carrier 1	Slc25a22	0.715	4.73622E-05	Down	0.871	0.888	0.891	1.21	1.276	1.221
P00405	Cytochrome c oxidase subunit 2	Mtco2	0.691	4.75001E-05	Down	0.83	0.839	0.852	1.172	1.239	1.236
Q9DCM2	Glutathione S-transferase kappa 1	Gstk1	0.708	4.88816E-05	Down	0.861	0.829	0.866	1.174	1.219	1.219
Q9D172	Glutamine amidotransferase-like class 1 domain-containing protein 3A,	Gatd3a	0.683	4.9773E-05	Down	0.788	0.813	0.774	1.188	1.129	1.162
Q9CR62	Mitochondrial 2-oxoglutarate/malate carrier protein	Slc25a11	0.764	5.20561E-05	Down	0.89	0.911	0.903	1.2	1.15	1.19
P11103	Poly [ADP-ribose] polymerase 1	Parp1	1.304	5.24559E-05	Up	0.965	0.932	0.93	0.714	0.734	0.72
P68040	Receptor of activated protein C kinase 1	Rack1	1.374	5.30012E-05	Up	1.196	1.201	1.187	0.85	0.899	0.86
P12787	Cytochrome c oxidase subunit 5A,	Cox5a	0.704	5.30384E-05	Down	0.891	0.854	0.86	1.201	1.26	1.239
Q9CQQ7	ATP synthase F(0) complex subunit B1,	Atp5pb	0.728	5.32126E-05	Down	0.879	0.88	0.866	1.209	1.233	1.165
Q60714	Long-chain fatty acid transport protein 1	Slc27a1	0.482	5.51299E-05	Down	0.799	0.813	0.812	1.567	1.666	1.799
Q9Z2I8	Succinate--CoA ligase [GDP-forming] subunit beta, mitochondrial	Suclg2	0.667	5.61723E-05	Down	0.885	0.86	0.83	1.315	1.258	1.29
Q9CQR4	Acyl-coenzyme A thioesterase 13	Acot13	0.726	5.80497E-05	Down	0.863	0.845	0.88	1.216	1.16	1.191

Q9Z1P6	NADH dehydrogenase [ubiquinone] 1 alpha subcomplex subunit 7	Ndufa7	0.724	5.96735E-05	Down	0.909	0.919	0.873	1.267	1.239	1.227
Q8R4N0	Citramalyl-CoA lyase, mitochondrial	Clybl	0.714	6.00245E-05	Down	0.864	0.862	0.874	1.254	1.212	1.176
Q9CQI6	Coactosin-like protein	Cotl1	1.92	6.02125E-05	Up	1.266	1.34	1.374	0.695	0.722	0.656
A6BLY7	Keratin, type I cytoskeletal 28	Krt28	0.656	6.3952E-05	Down	0.796	0.797	0.798	1.206	1.168	1.269
Q9QZ85	Interferon-inducible GTPase 1	Iigp1	0.69	6.48399E-05	Down	0.55	0.554	0.537	0.821	0.769	0.787
Q9QXX4	Calcium-binding mitochondrial carrier protein Aralar2	Slc25a13	0.672	6.80745E-05	Down	0.824	0.774	0.783	1.154	1.181	1.207
P29391	Ferritin light chain 1	Ftl1	3.792	6.94112E-05	Up	1.847	1.679	1.817	0.467	0.53	0.412
Q61093	Cytochrome b-245 heavy chain	Cybb	1.743	6.9785E-05	Up	1.295	1.341	1.251	0.738	0.779	0.713
Q0II04	Nebulette	Nebl	0.58	7.01608E-05	Down	0.768	0.83	0.765	1.31	1.38	1.385
O88441	Metaxin-2	Mtx2	0.603	7.02149E-05	Down	0.824	0.795	0.784	1.266	1.383	1.339
Q99L13	3-hydroxyisobutyrate dehydrogenase, mitochondrial	Hibadh	0.711	7.04145E-05	Down	0.907	0.929	0.877	1.27	1.297	1.248
P21550	Beta-enolase	Eno3	0.741	7.0742E-05	Down	0.843	0.825	0.816	1.138	1.13	1.085
P28650	Adenylosuccinate synthetase isozyme 1	Adss1	1.521	7.09072E-05	Up	1.171	1.178	1.172	0.81	0.756	0.749
Q8BJE2	Butyrophilin-like protein 9	Btnl9	0.749	7.14585E-05	Down	1.006	0.988	1.013	1.31	1.378	1.325
Q78J03	Methionine-R-sulfoxide reductase B2,	Msrb2	0.719	7.182E-05	Down	0.896	0.909	0.874	1.208	1.244	1.276
Q9JKF1	Ras GTPase-activating-like protein IQGAP1	Iqgap1	1.313	7.24896E-05	Up	1.22	1.165	1.169	0.909	0.907	0.891
Q07076	Annexin A7	Anxa7	1.372	7.45238E-05	Up	1.181	1.2	1.166	0.833	0.88	0.872
Q3URS9	Mitochondrial potassium channel	Ccdc51	0.677	7.69204E-05	Down	0.839	0.851	0.903	1.275	1.293	1.263
P20491	High affinity immunoglobulin epsilon receptor subunit gamma	Fcer1g	2.14	7.7115E-05	Up	1.148	1.128	1.176	0.586	0.518	0.509
P20152	Vimentin	Vim	1.308	8.06016E-05	Up	1.066	1.076	1.054	0.823	0.83	0.79
Q8CJ27	Abnormal spindle-like microcephaly-associated protein homolog	Aspm	0.52	8.2954E-05	Down	0.642	0.682	0.62	1.293	1.267	1.176
Q9JHI5	Isovaleryl-CoA dehydrogenase, mitochondrial	Ivd	0.745	8.3079E-05	Down	0.934	0.898	0.884	1.208	1.234	1.204
Q791V5	Mitochondrial carrier homolog 2	Mtch2	0.71	8.39881E-05	Down	0.854	0.882	0.907	1.268	1.218	1.234
O09114	Prostaglandin-H2 D-isomerase	Ptgds	0.644	8.4117E-05	Down	0.922	0.883	0.87	1.342	1.37	1.439
P30993	C5a anaphylatoxin chemotactic receptor 1	C5ar1	1.769	8.67779E-05	Up	1.28	1.415	1.351	0.733	0.785	0.769
Q9D1G3	Protein-cysteine N-palmitoyltransferase HHAT-like protein	Hhatl	0.546	8.70038E-05	Down	0.813	0.814	0.842	1.45	1.616	1.454
Q9DCW4	Electron transfer flavoprotein subunit beta	Etfb	0.712	8.84897E-05	Down	0.887	0.885	0.853	1.241	1.189	1.258
Q8BK08	Transmembrane protein 11, mitochondrial	Tmem11	0.655	8.99047E-05	Down	0.915	0.867	0.859	1.301	1.345	1.383
P53395	Lipoamide acyltransferase component of branched-chain alpha-keto acid dehydrogenase complex	Dbt	0.764	9.25295E-05	Down	0.924	0.899	0.886	1.18	1.16	1.208
P09405	Nucleolin	Ncl	1.317	9.66888E-05	Up	1.138	1.116	1.11	0.869	0.86	0.825
Q61233	Plastin-2	Lcp1	1.938	9.78119E-05	Up	1.378	1.346	1.466	0.7	0.77	0.692
Q9CXX9	CUE domain-containing protein 2	Cuedc2	0.434	9.98588E-05	Down	0.663	0.61	0.682	1.62	1.403	1.482
Q9DB20	ATP synthase subunit O, mitochondrial	Atp5po	0.732	0.000102846	Down	0.876	0.897	0.868	1.223	1.16	1.223
P16045	Galectin-1	Lgals1	1.307	0.000103614	Up	1.238	1.207	1.25	0.93	0.929	0.969
P07310	Creatine kinase M-type	Ckm	0.754	0.000103848	Down	0.838	0.807	0.817	1.113	1.059	1.095
P97807	Fumarate hydratase, mitochondrial	Fh	0.702	0.000108851	Down	0.884	0.876	0.825	1.212	1.248	1.224
Q8BMF4	Dihydrolipoyllysine-residue acetyltransferase component of pyruvate dehydrogenase complex,	Dlat	0.746	0.000114818	Down	0.903	0.885	0.855	1.158	1.204	1.18

Q9CR61	NADH dehydrogenase [ubiquinone] 1 beta subcomplex subunit 7	Ndufb7	0.755	0.000115561	Down	0.862	0.918	0.894	1.192	1.178	1.174
P60867	40S ribosomal protein S20	Rps20	1.313	0.000117761	Up	1.092	1.121	1.137	0.841	0.874	0.836
Q61074	Protein phosphatase 1G	Ppm1g	1.361	0.000118324	Up	1.207	1.192	1.149	0.853	0.894	0.86
Q9Z1Q5	Chloride intracellular channel protein 1	Clic1	1.377	0.000124576	Up	1.2	1.165	1.214	0.885	0.879	0.836
Q4ZJM7	Otolin-1	Otol1	0.622	0.000125227	Down	0.917	0.834	0.847	1.409	1.355	1.414
Q8BH61	Coagulation factor XIII A chain	F13a1	2.226	0.000127253	Up	1.48	1.371	1.468	0.638	0.706	0.596
Q9CXD6	Mitochondrial calcium uniporter regulator 1	Mcur1	0.714	0.000128831	Down	0.832	0.872	0.807	1.176	1.18	1.16
P10605	Cathepsin B	Ctsb	1.808	0.000132267	Up	1.32	1.425	1.494	0.81	0.759	0.775
Q8BH70	F-box/LRR-repeat protein 4	Fbxl4	0.436	0.000134591	Down	0.725	0.76	0.727	1.738	1.822	1.515
Q9D517	1-acyl-sn-glycerol-3-phosphate acyltransferase gamma	Agpat3	0.628	0.000134797	Down	0.852	0.86	0.888	1.3	1.426	1.416
Q71RI9	Kynurenine--oxoglutarate	Kyat3	0.652	0.000135134	Down	0.76	0.813	0.808	1.243	1.168	1.241
A2AJB2	Transmembrane protein 141	Tmem141	0.639	0.000138624	Down	0.812	0.866	0.87	1.283	1.322	1.384
Q811U4	Mitofusin-1	Mfn1	0.668	0.000140123	Down	0.835	0.867	0.846	1.331	1.217	1.264
P56501	Mitochondrial uncoupling protein 3	Ucp3	0.554	0.000140951	Down	0.859	0.876	0.776	1.483	1.565	1.485
P42925	Peroxisomal membrane protein 2	Pxmp2	0.686	0.000144365	Down	0.861	0.904	0.83	1.279	1.237	1.266
Q8BWY3	Eukaryotic peptide chain release factor subunit 1	Etf1	1.301	0.00014523	Up	1.137	1.104	1.158	0.885	0.878	0.85
P56379	ATP synthase subunit ATP5MPL,	Atp5mpl	0.73	0.000145585	Down	0.896	0.862	0.866	1.225	1.154	1.214
Q8K1Z0	Ubiquinone biosynthesis protein COQ9, mitochondrial	Coq9	0.683	0.000149053	Down	0.838	0.836	0.826	1.267	1.158	1.234
Q9D1A2	Cytosolic non-specific dipeptidase	Cndp2	1.363	0.000149928	Up	1.211	1.176	1.148	0.874	0.838	0.882
Q9D2G2	Dihydrolipoyllysine-residue succinyltransferase component of 2-oxoglutarate	Dlst	0.72	0.000156223	Down	0.843	0.877	0.909	1.222	1.235	1.196
Q3TZ89	Protein transport protein Sec31B	Sec31b	0.766	0.000157144	Down	0.981	0.94	0.974	1.292	1.257	1.231
Q9D273	Corrinoid adenosyltransferase	Mmab	0.73	0.000159619	Down	0.802	0.787	0.843	1.095	1.132	1.104
Q91V16	Electron transfer flavoprotein regulatory factor 1	Etfrf1	0.737	0.000161185	Down	0.927	0.869	0.892	1.238	1.221	1.189
P50752	Troponin T, cardiac muscle	Tnnt2	0.519	0.000161449	Down	0.628	0.735	0.67	1.283	1.341	1.29
Q9D3B1	Very-long-chain (3R)-3-hydroxyacyl-CoA dehydratase 2	Hacd2	0.578	0.00016856	Down	0.785	0.791	0.793	1.443	1.391	1.262
P63028	Translationally-controlled tumor protein	Tpt1	1.422	0.000175563	Up	1.288	1.203	1.207	0.874	0.883	0.844
Q9CQ62	2,4-dienoyl-CoA reductase, mitochondrial	Decr1	0.709	0.000177122	Down	0.923	0.889	0.856	1.242	1.288	1.233
P34914	Bifunctional epoxide hydrolase 2	Ephx2	0.762	0.000179918	Down	0.928	0.893	0.954	1.205	1.207	1.231
P43023	Cytochrome c oxidase subunit 6A2,	Cox6a2	0.687	0.000182493	Down	0.881	0.934	0.862	1.267	1.294	1.333
Q9DCV4	Regulator of microtubule dynamics protein 1	Rmdn1	0.603	0.000183676	Down	0.832	0.833	0.745	1.309	1.347	1.343
O54794	Aquaporin-7	Aqp7	0.699	0.00018475	Down	0.942	0.936	0.887	1.326	1.273	1.359
O70251	Elongation factor 1-beta	Eef1b	1.319	0.000184878	Up	1.14	1.129	1.088	0.839	0.874	0.832
Q9DC70	NADH dehydrogenase [ubiquinone] iron-sulfur protein 7, mitochondrial	Ndufs7	0.732	0.000190007	Down	0.902	0.847	0.87	1.198	1.159	1.22
O89053	Coronin-1A	Coro1a	1.733	0.000190864	Up	1.083	1.02	1.051	0.644	0.611	0.565
P24270	Catalase	Cat	0.642	0.000195999	Down	0.893	0.863	0.86	1.441	1.294	1.342
P47226	Testin	Tes	1.432	0.000197378	Up	1.252	1.186	1.275	0.872	0.836	0.884
Q9CPQ3	Mitochondrial import receptor subunit TOM22 homolog	Tomm22	0.657	0.000198073	Down	0.85	0.921	0.847	1.31	1.302	1.374
O89017	Legumain	Lgmn	4.733	0.000199858	Up	1.833	2.033	2.013	0.331	0.418	0.493

P62073	Mitochondrial import inner membrane translocase subunit	Timm10	0.726	0.000201008	Down	0.825	0.865	0.887	1.18	1.16	1.211
Q9CQA3	Succinate dehydrogenase [ubiquinone] iron-sulfur subunit, mitochondrial	Sdhb	0.736	0.000205118	Down	0.943	0.886	0.885	1.252	1.205	1.232
Q9DCX2	ATP synthase subunit d, mitochondrial	Atp5pd	0.708	0.000205313	Down	0.885	0.925	0.845	1.245	1.241	1.264
Q63810	Calcineurin subunit B type 1	Ppp3r1	1.334	0.000205599	Up	1.157	1.177	1.143	0.839	0.901	0.866
Q9Z0J0	NPC intracellular cholesterol transporter 2	Npc2	1.534	0.000208615	Up	1.264	1.24	1.27	0.777	0.869	0.814
P15105	Glutamine synthetase	Glul	1.465	0.000209084	Up	1.255	1.259	1.368	0.883	0.87	0.897
Q8BMF3	NADP-dependent malic enzyme, mitochondrial	Me3	0.701	0.000211775	Down	0.9	0.862	0.847	1.193	1.248	1.282
Q9ERS2	NADH dehydrogenase [ubiquinone] 1 alpha subcomplex subunit 13	Ndufa13	0.755	0.000221192	Down	0.889	0.916	0.849	1.168	1.176	1.172
Q8BP40	Lysophosphatidic acid phosphatase type 6	Acp6	0.636	0.000228805	Down	0.821	0.773	0.873	1.284	1.28	1.312
Q61599	Rho GDP-dissociation inhibitor 2	Arhgdib	1.367	0.000230244	Up	1.122	1.103	1.068	0.771	0.822	0.816
Q91VR2	ATP synthase subunit gamma, mitochondrial	Atp5f1c	0.698	0.0002348	Down	0.86	0.865	0.84	1.268	1.16	1.246
Q9R0H2	Endomucin	Emcn	0.62	0.000236908	Down	0.713	0.759	0.759	1.13	1.262	1.204
P98192	Dihydroxyacetone phosphate acyltransferase	Gnpat	0.655	0.000252491	Down	0.887	0.876	0.892	1.262	1.404	1.39
Q99JX4	Eukaryotic translation initiation factor 3 subunit	Eif3m	1.35	0.000256385	Up	1.118	1.206	1.146	0.874	0.85	0.846
Q8R0F8	Acylpyruvase FAHD1, mitochondrial	Fahd1	0.726	0.000257114	Down	0.893	0.921	0.888	1.3	1.207	1.216
Q08943	FACT complex subunit SSRP1	Ssrp1	1.338	0.000260499	Up	0.893	0.907	0.964	0.694	0.682	0.689
Q922F4	Tubulin beta-6 chain	Tubb6	1.659	0.000260948	Up	1.228	1.255	1.196	0.7	0.797	0.721
P14206	40S ribosomal protein SA	Rpsa	1.392	0.000262865	Up	1.186	1.153	1.19	0.804	0.875	0.856
P11835	Integrin beta-2	Itgb2	2.285	0.000270222	Up	1.495	1.448	1.531	0.724	0.659	0.575
Q8BKZ9	Pyruvate dehydrogenase protein X component, mitochondrial	Pdhx	0.735	0.000273839	Down	0.85	0.9	0.841	1.167	1.15	1.208
P70677	Caspase-3	Casp3	1.449	0.000281745	Up	1.114	1.146	1.088	0.79	0.792	0.729
P97929	Breast cancer type 2 susceptibility protein homolog	Brca2	0.55	0.000283467	Down	0.888	0.857	0.826	1.454	1.696	1.526
P97443	Histone-lysine N-methyltransferase Smyd1	Smyd1	0.609	0.000283961	Down	0.769	0.745	0.792	1.259	1.181	1.345
Q9CXI0	2-methoxy-6-polyprenyl-1,4-benzoquinol methylase, mitochondrial	Coq5	0.718	0.000288028	Down	0.849	0.805	0.854	1.132	1.15	1.212
Q8VE95	UPF0598 protein C8orf82 homolog	--	0.702	0.000289591	Down	0.837	0.853	0.908	1.231	1.27	1.198
P56392	Cytochrome c oxidase subunit 7A1,	Cox7a1	0.669	0.000293237	Down	0.795	0.843	0.82	1.16	1.284	1.231
Q9CQ54	NADH dehydrogenase [ubiquinone] 1 subunit	Ndufc2	0.758	0.000293922	Down	0.903	0.893	0.903	1.2	1.134	1.225
Q64433	10 kDa heat shock protein, mitochondrial	Hspe1	0.719	0.000295336	Down	0.842	0.82	0.777	1.144	1.15	1.097
Q61827	Transcription factor MafK	Mafk	0.736	0.000302165	Down	0.671	0.706	0.704	0.948	0.907	0.972
P56393	Cytochrome c oxidase subunit 7B,	Cox7b	0.604	0.000306061	Down	0.831	0.771	0.739	1.303	1.342	1.228
Q9WVJ3	Carboxypeptidase Q	Cpq	1.303	0.000307701	Up	1.147	1.172	1.236	0.902	0.913	0.914
Q91YY4	ATP synthase mitochondrial F1 complex assembly factor	Atpaf2	0.695	0.000315988	Down	0.97	0.881	0.907	1.324	1.351	1.291
Q91WS0	CDGSH iron-sulfur domain-containing	Cisd1	0.645	0.000322691	Down	0.792	0.867	0.851	1.36	1.243	1.288
Q9D6K8	FUN14 domain-containing protein 2	Fundc2	0.664	0.000326788	Down	0.861	0.798	0.811	1.204	1.308	1.208
Q91V79	Fat storage-inducing transmembrane protein 1	Fitm1	0.524	0.000327468	Down	0.707	0.789	0.754	1.413	1.327	1.556
Q9DCS9	NADH dehydrogenase [ubiquinone] 1 beta subcomplex subunit 10	Ndufb10	0.73	0.000337961	Down	0.926	0.849	0.886	1.206	1.244	1.197
Q9JJU8	SH3 domain-binding glutamic acid-rich-like protein	Sh3bgrl	1.308	0.000339256	Up	1.266	1.2	1.282	0.977	0.938	0.95

P52503	NADH dehydrogenase [ubiquinone] iron-sulfur protein 6, mitochondrial	Ndufs6	0.725	0.0003413	Down	0.926	0.871	0.852	1.24	1.227	1.186
Q8JZL7	Ras-GEF domain-containing family	Rasgef1b	0.681	0.000343551	Down	0.858	0.906	0.841	1.303	1.211	1.311
P10107	Annexin A1	Anxa1	1.392	0.000365199	Up	1.235	1.27	1.182	0.857	0.919	0.872
Q9ERI2	Ras-related protein Rab-27A	Rab27a	1.305	0.000365743	Up	1.158	1.216	1.18	0.942	0.884	0.898
Q9CXS4	Centromere protein V	Cenpv	0.706	0.000368133	Down	0.661	0.674	0.613	0.899	0.936	0.924
O35129	Prohibitin-2	Phb2	0.763	0.000368877	Down	0.917	0.918	0.872	1.224	1.157	1.168
O08600	Endonuclease G, mitochondrial	Endog	0.767	0.0003714	Down	0.89	0.928	0.937	1.16	1.198	1.234
Q99MV1	Tudor domain-containing protein 1	Tdrd1	1.609	0.000374045	Up	1.403	1.377	1.248	0.81	0.872	0.822
Q91VN4	MICOS complex subunit Mic25	Chchd6	0.739	0.000375138	Down	0.962	0.883	0.898	1.219	1.244	1.251
P14901	Heme oxygenase 1	Hmox1	2.017	0.000377149	Up	1.538	1.469	1.514	0.66	0.768	0.813
Q9D3D9	ATP synthase subunit delta, mitochondrial	Atp5f1d	0.692	0.000378222	Down	0.889	0.796	0.844	1.197	1.222	1.238
P07091	Protein S100-A4	S100a4	2.335	0.00038036	Up	1.641	1.742	1.467	0.774	0.643	0.66
Q9CQ24	F-box only protein 36	Fbxo36	0.75	0.00038158	Down	0.877	0.88	0.949	1.202	1.211	1.194
O89086	RNA-binding protein 3	Rbm3	1.358	0.000384585	Up	1.255	1.235	1.203	0.948	0.87	0.901
O09131	Glutathione S-transferase omega-1	Gsto1	1.312	0.000391194	Up	1.166	1.148	1.09	0.864	0.886	0.844
P18760	Cofilin-1	Cfl1	1.357	0.000396234	Up	1.194	1.123	1.139	0.871	0.861	0.814
P63242	Eukaryotic translation initiation factor 5A-1	Eif5a	1.322	0.000401165	Up	1.136	1.091	1.168	0.868	0.872	0.829
Q8K199	COX assembly mitochondrial protein 2 homolog	Cmc2	0.53	0.000412039	Down	0.68	0.762	0.716	1.465	1.24	1.368
Q9DCJ5	NADH dehydrogenase [ubiquinone] 1 alpha subcomplex subunit 8	Ndufa8	0.753	0.000416655	Down	0.889	0.891	0.837	1.122	1.166	1.186
Q8K2Q5	Coiled-coil-helix-coiled-coil-helix domain-containing protein 7	Chchd7	0.71	0.000427231	Down	0.739	0.766	0.726	1.073	0.99	1.078
P29341	Polyadenylate-binding protein 1	Pabpc1	1.349	0.00042758	Up	1.155	1.084	1.108	0.816	0.861	0.804
Q61387	Cytochrome c oxidase subunit 7A-related protein, mitochondrial	Cox7a2l	0.769	0.000433097	Down	0.915	0.887	0.888	1.156	1.216	1.127
Q9CZ13	Cytochrome b-c1 complex subunit 1,	Uqcrc1	0.755	0.000436246	Down	0.863	0.9	0.875	1.202	1.113	1.181
P30412	Peptidyl-prolyl cis-trans isomerase C	Ppic	1.478	0.000436662	Up	1.167	1.107	1.195	0.791	0.816	0.74
Q78RX3	Small integral membrane protein 12	Smim12	0.769	0.000440799	Down	0.888	0.951	0.943	1.178	1.226	1.215
P11276	Fibronectin	Fn1	1.392	0.000454335	Up	1.243	1.208	1.237	0.852	0.861	0.937
A6PWD2	Forkhead-associated domain-containing	Fhad1	0.567	0.000458035	Down	0.704	0.715	0.679	1.116	1.328	1.259
Q6R5N8	Toll-like receptor 13	Tlr13	0.71	0.000460418	Down	0.844	0.916	0.826	1.212	1.232	1.197
Q8K370	Acyl-CoA dehydrogenase family member 10	Acad10	0.732	0.000473687	Down	0.725	0.765	0.706	1.014	1.022	0.964
Q9D706	RNA polymerase II-associated protein 3	Rpap3	1.322	0.000492195	Up	1.062	1.151	1.066	0.829	0.834	0.817
Q6P6M7	O-phosphoseryl-tRNA(Sec) selenium transferase	Sepsecs	0.676	0.000496199	Down	0.915	0.828	0.83	1.306	1.276	1.224
P55096	ATP-binding cassette sub-family D member 3	Abcd3	0.669	0.000498218	Down	0.851	0.914	0.872	1.338	1.232	1.374
P12265	Beta-glucuronidase	Gusb	1.316	0.000504568	Up	1.126	1.184	1.172	0.849	0.88	0.916
Q9WTP6	Adenylate kinase 2, mitochondrial	Ak2	0.762	0.000506508	Down	0.909	0.873	0.902	1.127	1.222	1.174
P08030	Adenine phosphoribosyltransferas	Aprt	1.394	0.000516603	Up	1.137	1.106	1.217	0.836	0.802	0.844
P00416	Cytochrome c oxidase subunit 3	mt-Co3	0.612	0.000519281	Down	0.829	0.739	0.82	1.264	1.383	1.255
Q8C1Q6	Small integral membrane protein 4	Smim4	0.54	0.00053005	Down	0.745	0.849	0.762	1.471	1.334	1.558
Q8K558	Trem-like transcript 1 protein	Treml1	0.698	0.000539649	Down	0.742	0.833	0.774	1.133	1.096	1.134
Q9Z0N2	Eukaryotic translation initiation factor 2 subunit 3, Y-linked	Eif2s3y	1.51	0.000542873	Up	1.33	1.342	1.308	0.812	0.894	0.93

Q9D1N2	Glycosylphosphatidylinositol-anchored high density lipoprotein-	Gpihbp1	0.529	0.000543005	Down	0.829	0.835	0.697	1.536	1.506	1.422
Q9JLR1	Protein transport protein Sec61 subunit alpha isoform 2	Sec61a2	0.721	0.000546175	Down	0.831	0.864	0.891	1.181	1.151	1.254
Q61878	Bone marrow proteoglycan	Prg2	2.901	0.000549517	Up	1.774	1.561	1.791	0.488	0.681	0.598
Q80ZU0	ADP-ribosylation factor-like protein 5A	Arl5a	0.509	0.000550567	Down	0.631	0.593	0.697	1.366	1.157	1.252
P46656	Adrenodoxin, mitochondrial	Fdx1	0.718	0.000552448	Down	0.88	0.888	0.868	1.285	1.235	1.151
P39749	Flap endonuclease 1	Fen1	1.473	0.000559655	Up	1.275	1.416	1.33	0.874	0.906	0.949
Q9JII6	Aldo-keto reductase family 1 member A1	Akr1a1	1.304	0.000569637	Up	1.223	1.193	1.241	0.918	0.907	0.98
Q8VD26	Transmembrane protein 143	Tmem143	0.684	0.000571682	Down	0.754	0.829	0.749	1.144	1.094	1.169
P47802	Metaxin-1	Mtx1	0.766	0.000579888	Down	0.895	0.901	0.907	1.239	1.148	1.141
P03903	NADH-ubiquinone oxidoreductase chain 4L	Mtnd4l	0.713	0.000585656	Down	0.843	0.894	0.915	1.189	1.239	1.292
P33610	DNA primase large subunit	Prim2	0.705	0.000589403	Down	0.881	0.876	0.842	1.264	1.272	1.151
Q9CQH3	NADH dehydrogenase [ubiquinone] 1 beta subcomplex subunit 5, mitochondrial	Ndufb5	0.769	0.000599185	Down	0.937	0.879	0.895	1.206	1.133	1.188
Q62351	Transferrin receptor protein 1	Tfrc	1.373	0.000628405	Up	1.275	1.22	1.343	0.91	0.963	0.923
E9Q9K5	Triadin	Trdn	0.53	0.000634893	Down	0.756	0.907	0.84	1.689	1.56	1.476
P08074	Carbonyl reductase [NADPH] 2	Cbr2	1.492	0.000638893	Up	1.038	1.011	1.138	0.732	0.684	0.72
Q9QVP4	Myosin regulatory light chain 2, atrial isoform	Myl7	0.766	0.000643097	Down	0.978	1.037	0.986	1.352	1.259	1.305
O55126	Protein NipSnap homolog 2	Nipsnap2	0.765	0.000659021	Down	0.969	0.916	0.915	1.178	1.265	1.219
Q9WUU7	Cathepsin Z	Ctsz	1.31	0.000664219	Up	1.089	1.146	1.163	0.89	0.832	0.872
P48787	Troponin I, cardiac muscle	Tnni3	0.523	0.000666453	Down	0.691	0.639	0.629	1.341	1.103	1.303
Q99NH2	Partitioning defective 3 homolog	Pard3	0.695	0.000677138	Down	0.875	0.904	0.823	1.303	1.189	1.253
P70195	Proteasome subunit beta type-7	Psmb7	1.376	0.000709879	Up	1.337	1.244	1.281	0.909	0.986	0.911
P43883	Perilipin-2	Plin2	0.647	0.000723475	Down	0.814	0.928	0.85	1.332	1.277	1.398
P98078	Disabled homolog 2	Dab2	1.395	0.000725539	Up	1.127	1.144	1.126	0.766	0.865	0.804
P03921	NADH-ubiquinone oxidoreductase chain 5	Mtnd5	0.742	0.000729703	Down	0.904	0.87	0.844	1.121	1.22	1.188
Q8K2V1	Serine/threonine-protein phosphatase 4 regulatory subunit 1	Ppp4r1	1.6	0.000747988	Up	1.173	1.176	1.343	0.76	0.743	0.804
O35143	ATPase inhibitor, mitochondrial	ATP5IF1	0.724	0.00075228	Down	0.841	0.898	0.915	1.172	1.272	1.221
Q06185	ATP synthase subunit e, mitochondrial	Atp5me	0.688	0.000760764	Down	0.889	0.826	0.866	1.206	1.338	1.209
Q9JKL4	NADH dehydrogenase [ubiquinone] 1 alpha subcomplex assembly factor 3	Ndufaf3	0.715	0.000777894	Down	0.844	0.852	0.899	1.279	1.196	1.152
Q8R086	Sulfite oxidase, mitochondrial	Suox	0.766	0.000786767	Down	0.878	0.909	0.839	1.133	1.114	1.182
Q9CQV8	14-3-3 protein beta/alpha	Ywhab	1.303	0.000812749	Up	1.18	1.086	1.18	0.896	0.879	0.869
Q3TWW8	Serine/arginine-rich splicing factor 6	Srsf6	1.574	0.000814291	Up	1.366	1.321	1.211	0.775	0.827	0.874
P14069	Protein S100-A6	S100a6	1.72	0.000824627	Up	1.256	1.398	1.484	0.857	0.784	0.765
Q9QXS6	Drebrin	Dbn1	1.544	0.000829798	Up	1.289	1.292	1.266	0.898	0.833	0.761
Q9JLJ5	Elongation of very long chain fatty acids protein	Elovl1	0.7	0.000848717	Down	0.781	0.781	0.859	1.102	1.196	1.159
Q6NS65	Uracil nucleotide/cysteinyl	Gpr17	0.42	0.000860937	Down	0.627	0.456	0.487	1.27	1.214	1.256
Q9WV98	Mitochondrial import inner membrane translocase subunit Tim9	Timm9	0.693	0.00086463	Down	0.811	0.839	0.857	1.24	1.118	1.261
Q9Z1W8	Potassium-transporting ATPase alpha chain 2	Atp12a	0.741	0.000882091	Down	0.927	0.942	0.847	1.239	1.219	1.209
P21614	Vitamin D-binding protein	Gc	1.413	0.000887084	Up	1.289	1.232	1.302	0.857	0.885	0.964
Q9CQJ8	NADH dehydrogenase [ubiquinone] 1 beta subcomplex subunit 9	Ndufb9	0.767	0.000891277	Down	0.872	0.942	0.933	1.17	1.236	1.176

Q8K215	LYR motif-containing protein 4	Lyrm4	0.746	0.000930325	Down	0.931	0.858	0.922	1.185	1.263	1.185
Q8VEE4	Replication protein A 70 kDa DNA-binding	Rpa1	1.357	0.000932634	Up	1.137	1.115	1.204	0.891	0.841	0.815
Q9D5T0	ATPase family AAA domain-containing	Atad1	0.642	0.000938895	Down	0.757	0.846	0.82	1.31	1.164	1.301
Q9CQI3	Glia maturation factor beta	Gmfb	1.373	0.00094385	Up	1.176	1.138	1.147	0.893	0.791	0.836
Q4VAE3	Transmembrane protein 65	Tmem65	0.754	0.000986165	Down	0.862	0.878	0.873	1.135	1.227	1.102
P68037	Ubiquitin-conjugating enzyme E2 L3	Ube2l3	1.304	0.001013465	Up	1.216	1.172	1.113	0.926	0.879	0.88
P62858	40S ribosomal protein S28	Rps28	1.302	0.001013719	Up	1.118	1.175	1.172	0.891	0.845	0.925
P47962	60S ribosomal protein L5	Rpl5	1.38	0.001019654	Up	1.14	1.124	1.109	0.87	0.808	0.766
Q8BHC4	Dephospho-CoA kinase domain-containing	Dcakd	0.735	0.001045979	Down	0.898	0.968	0.902	1.305	1.192	1.269
Q9CQ69	Cytochrome b-c1 complex subunit 8	Uqcrq	0.739	0.001063371	Down	0.89	0.896	0.872	1.238	1.117	1.24
Q61462	Cytochrome b-245 light chain	Cyba	1.746	0.001078398	Up	1.36	1.301	1.279	0.701	0.851	0.704
Q64516	Glycerol kinase	Gk	0.633	0.001082847	Down	0.762	0.818	0.76	1.308	1.118	1.273
Q9CQ00	Distal membrane-arm assembly complex	Dmac1	0.575	0.001086303	Down	0.738	0.846	0.828	1.288	1.378	1.53
Q62159	Rho-related GTP-binding protein RhoC	Rhoc	1.31	0.001087721	Up	1.204	1.107	1.169	0.918	0.883	0.855
Q78IK2	ATP synthase membrane subunit DAPIT, mitochondrial	Atp5md	0.719	0.00109471	Down	0.864	0.917	0.844	1.28	1.152	1.219
Q9CPQ1	Cytochrome c oxidase subunit 6C	Cox6c	0.743	0.001105753	Down	0.892	0.901	0.828	1.2	1.122	1.207
Q8CCM6	Mitochondrial import inner membrane translocase subunit	Timm21	0.709	0.001125204	Down	0.858	0.804	0.914	1.218	1.17	1.246
Q8BVZ1	Perilipin-5	Plin5	0.556	0.001145002	Down	0.885	0.699	0.831	1.477	1.425	1.445
Q60649	Caseinolytic peptidase B protein homolog	Clpb	0.708	0.001181807	Down	0.972	0.931	0.843	1.294	1.297	1.29
Q9JI71	Delta-like protein 4	Dll4	0.728	0.001205283	Down	0.806	0.776	0.845	1.176	1.094	1.064
Q8VE38	Oxidoreductase NAD-binding domain-containing protein 1	Oxnad1	0.751	0.001222379	Down	0.844	0.854	0.903	1.163	1.095	1.206
Q9EPT5	Solute carrier organic anion transporter family member 2A1	Slco2a1	1.45	0.001222782	Up	1.554	1.532	1.515	0.965	1.099	1.11
Q91XE4	N-acyl-aromatic-L-amino acid amidohydrolase (carboxylate-forming)	Acy3	1.425	0.001247851	Up	1.324	1.239	1.344	0.893	0.87	0.979
Q9CPU4	Microsomal glutathione S-transferase 3	Mgst3	0.675	0.001261418	Down	0.924	0.91	0.922	1.239	1.457	1.388
Q8R1F1	Protein Niban 2	Niban2	1.314	0.001264509	Up	1.229	1.142	1.178	0.938	0.857	0.905
Q8CC21	Tetratricopeptide repeat protein 19, mitochondrial	Ttc19	0.61	0.001276108	Down	0.771	0.867	0.951	1.393	1.441	1.407
Q5SUC9	Protein SCO1 homolog, mitochondrial	Sco1	0.603	0.001299418	Down	0.889	0.779	0.78	1.38	1.443	1.239
Q3UUI3	Acyl-coenzyme A thioesterase THEM4	Them4	0.703	0.001324457	Down	0.962	0.84	0.858	1.291	1.26	1.232
P03930	ATP synthase protein 8	Mtatp8	0.672	0.00133074	Down	0.784	0.792	0.867	1.261	1.122	1.255
O54942	Claudin-5	Cldn5	0.735	0.001331737	Down	0.954	0.946	0.903	1.216	1.36	1.24
O08528	Hexokinase-2	Hk2	0.728	0.001350468	Down	0.845	0.927	0.904	1.207	1.175	1.294
Q91VI7	Ribonuclease inhibitor	Rnh1	1.312	0.001385922	Up	1.131	1.169	1.219	0.857	0.939	0.886
P29351	Tyrosine-protein phosphatase non-receptor type 6	Ptpn6	1.496	0.001387814	Up	1.13	1.315	1.213	0.774	0.836	0.836
Q01149	Collagen alpha-2(I) chain	Col1a2	1.347	0.001403724	Up	1.452	1.316	1.361	1.007	1.071	0.988
Q9DBN5	Lon protease homolog 2, peroxisomal	Lonp2	0.641	0.001409066	Down	0.839	0.924	0.834	1.454	1.356	1.242
P11031	Activated RNA polymerase II transcriptional	Sub1	1.363	0.001417202	Up	1.036	0.995	1.116	0.758	0.801	0.75
Q3UW53	Protein Niban 1	Niban1	1.37	0.001419131	Up	1.074	1.122	1.195	0.864	0.792	0.82
P05125	Natriuretic peptides A	Nppa	0.762	0.001439036	Down	0.92	1.024	0.992	1.289	1.25	1.312
Q9CX80	Cytoglobin	Cygb	1.353	0.001450221	Up	1.235	1.268	1.25	0.901	0.995	0.878
P59266	Fat storage-inducing transmembrane protein 2	Fitm2	0.664	0.001484821	Down	0.838	0.813	0.956	1.262	1.324	1.341

Q8K093	Thyrotropin-releasing hormone-degrading ectoenzyme	Trhde	2.277	0.00151278	Up	1.315	1.126	1.266	0.612	0.571	0.445
P00848	ATP synthase subunit a	Mtatp6	0.757	0.001550183	Down	0.884	0.913	0.824	1.175	1.178	1.108
Q05920	Pyruvate carboxylase, mitochondrial	Pc	0.756	0.001552543	Down	0.843	0.876	0.783	1.107	1.071	1.131
Q8BZM1	Glomulin	Glmn	1.305	0.001560325	Up	1.177	1.154	1.184	0.842	0.906	0.946
Q91ZR1	Ras-related protein Rab-4B	Rab4b	1.316	0.001567844	Up	1.145	1.184	1.103	0.878	0.821	0.908
Q6WKZ8	E3 ubiquitin-protein ligase UBR2	Ubr2	1.341	0.00156819	Up	1.108	1.111	1.142	0.806	0.802	0.899
Q9D6S7	Ribosome-recycling factor, mitochondrial	Mrrf	0.734	0.001595989	Down	0.83	0.952	0.867	1.208	1.211	1.19
Q9CYH2	Peroxiredoxin-like 2A	Prxl2a	0.699	0.001596151	Down	0.876	0.833	0.766	1.126	1.181	1.233
Q9D2D9	Kelch domain-containing protein 8B	Klhdc8b	0.652	0.001623263	Down	0.849	0.856	0.764	1.183	1.367	1.236
Q9DCJ9	N-acetylneuraminate lyase	Npl	1.51	0.001624363	Up	1.179	1.305	1.281	0.759	0.872	0.863
P97821	Dipeptidyl peptidase 1	Ctsc	1.738	0.001724825	Up	1.352	1.255	1.255	0.68	0.694	0.848
Q91WR5	Aldo-keto reductase family 1 member C21	Akr1c21	1.372	0.001726482	Up	1.125	1.071	1.181	0.84	0.77	0.852
Q91YM2	Rho GTPase-activating protein 35	Arhgap35	0.751	0.001738095	Down	0.905	0.802	0.835	1.13	1.098	1.157
P48410	ATP-binding cassette sub-family D member 1	Abcd1	0.717	0.001767367	Down	0.864	0.837	0.909	1.124	1.255	1.263
Q9CWS0	N(G),N(G)-dimethylarginine	Ddah1	1.313	0.001774668	Up	1.201	1.19	1.28	0.918	0.893	0.984
P61924	Coatomer subunit zeta-1	Copz1	1.334	0.001778788	Up	1.188	1.169	1.178	0.849	0.953	0.847
P16675	Lysosomal protective protein	Ctsa	1.369	0.001798405	Up	1.228	1.214	1.352	0.966	0.92	0.885
Q9CPQ8	ATP synthase subunit g, mitochondrial	Atp5mg	0.735	0.00180975	Down	0.911	0.824	0.902	1.162	1.262	1.165
O35435	Dihydroorotate dehydrogenase (quinone), mitochondrial	Dhodh	0.748	0.001812001	Down	0.865	0.955	0.929	1.285	1.175	1.214
O70370	Cathepsin S	Ctss	1.602	0.001812216	Up	1.348	1.478	1.35	0.804	0.968	0.835
Q8BH86	D-glutamate cyclase, mitochondrial	Dglucy	0.748	0.001842107	Down	0.858	0.902	0.825	1.087	1.203	1.167
O09174	Alpha-methylacyl-CoA racemase	Amacr	0.746	0.001871545	Down	0.878	0.86	0.844	1.148	1.233	1.08
P19258	Protein Mpv17	Mpv17	0.683	0.00189662	Down	0.908	0.895	0.841	1.277	1.401	1.193
P21107	Tropomyosin alpha-3 chain	Tpm3	0.752	0.001900827	Down	0.81	0.792	0.786	1.138	1.002	1.037
Q5U458	DnaJ homolog subfamily C member 11	Dnajc11	0.728	0.001933182	Down	0.962	0.831	0.865	1.226	1.202	1.223
Q8R0Y8	Mitochondrial coenzyme A transporter SLC25A42	Slc25a42	0.676	0.002001413	Down	0.754	0.825	0.91	1.206	1.238	1.238
P61358	60S ribosomal protein L27	Rpl27	1.339	0.002069224	Up	0.937	0.996	0.916	0.752	0.704	0.672
Q8QZX0	Serine/threonine-protein kinase SBK1	Sbk1	0.758	0.002097957	Down	0.844	0.852	0.807	1.025	1.14	1.139
P34928	Apolipoprotein C-I	Apoc1	1.533	0.002098784	Up	1.53	1.44	1.482	0.863	1.05	0.991
Q9D6U8	Protein FAM162A	Fam162a	0.732	0.002128398	Down	0.882	0.969	0.913	1.173	1.306	1.295
Q99J39	Malonyl-CoA decarboxylase, mitochondrial	Mlycd	0.749	0.002162556	Down	0.869	0.851	0.948	1.133	1.232	1.195
Q9WVA2	Mitochondrial import inner membrane translocase subunit Tim8	Timm8a1	0.714	0.002175413	Down	0.808	0.92	0.808	1.138	1.188	1.224
O70571	[Pyruvate dehydrogenase (acetyl-transferring)] kinase isozyme 4,	Pdk4	0.738	0.002202556	Down	0.955	0.982	0.883	1.216	1.345	1.259
Q91VS7	Microsomal glutathione S-transferase 1	Mgst1	0.727	0.002214326	Down	0.903	1.021	0.993	1.268	1.382	1.364
Q60692	Proteasome subunit beta type-6	Psmb6	1.342	0.00225564	Up	1.219	1.269	1.221	0.886	0.997	0.88
P12790	Cytochrome P450 2B9	Cyp2b9	0.519	0.002309547	Down	0.68	0.675	0.633	1.38	1.055	1.392
Q5QGU6	Receptor-transporting protein 3	Rtp3	0.758	0.002324929	Down	0.786	0.863	0.793	1.099	1.107	1.017
Q91WC0	Actin-histidine N-methyltransferase	Setd3	1.318	0.00241564	Up	1.093	1.098	1.206	0.818	0.887	0.872
P50149	Guanine nucleotide-binding protein G(t)	Gnat2	0.701	0.002461738	Down	1.176	1.118	1.152	1.642	1.496	1.778
Q8R2Y8	Peptidyl-tRNA hydrolase 2, mitochondrial	Ptrh2	0.738	0.002461912	Down	0.936	0.912	0.976	1.253	1.199	1.373
Q9R0N0	Galactokinase	Galk1	1.31	0.002531195	Up	1.152	1.067	1.188	0.85	0.842	0.909

P03893	NADH-ubiquinone oxidoreductase chain 2	Mtnd2	0.646	0.00258781	Down	0.871	0.887	0.828	1.197	1.481	1.327
Q2VLH6	Scavenger receptor cysteine-rich type 1 protein M130	Cd163	1.903	0.00260529	Up	1.345	1.194	1.418	0.649	0.81	0.62
Q80W47	WD repeat domain phosphoinositide-interacting protein 2	Wipi2	1.307	0.002694151	Up	1.129	1.224	1.075	0.898	0.859	0.865
P02104	Hemoglobin subunit epsilon-Y2	Hbb-y	1.519	0.002710622	Up	1.101	1.151	1.214	0.73	0.704	0.848
O09172	Glutamate--cysteine ligase regulatory subunit	Gclm	1.354	0.002714279	Up	1.037	1.124	1.106	0.855	0.81	0.748
Q61576	Peptidyl-prolyl cis-trans isomerase FKBP10	Fkbp10	1.345	0.002752041	Up	1.149	1.244	1.276	0.923	0.951	0.854
Q91VZ6	Stromal membrane-associated protein 1	Smap1	1.315	0.002756241	Up	1.126	1.152	1.162	0.92	0.892	0.804
Q8R0W0	Epiplakin	Eppk1	1.353	0.00277044	Up	1.255	1.261	1.186	0.887	0.988	0.861
Q9ET66	Peptidase inhibitor 16	Pi16	1.404	0.002773175	Up	1.189	1.203	1.358	0.942	0.857	0.872
Q3UPH1	Protein PRRC1	Prrc1	1.402	0.002788461	Up	1.3	1.272	1.169	0.929	0.92	0.82
Q99P30	Peroxisomal coenzyme A diphosphatase NUDT7	Nudt7	0.746	0.002792572	Down	0.844	0.981	0.919	1.242	1.199	1.239
P62075	Mitochondrial import inner membrane translocase subunit	Timm13	0.728	0.002805038	Down	0.8	0.761	0.85	1.059	1.185	1.067
Q8BFQ8	Glutamine amidotransferase-like class 1 domain-	Gatd1	1.335	0.002827803	Up	1.119	1.148	1.119	0.829	0.917	0.791
P14430	H-2 class I histocompatibility antigen, Q8 alpha chain	H2-Q8	0.767	0.002850194	Down	0.794	0.706	0.705	0.958	0.94	0.975
Q8BGS2	BolA-like protein 2	Bola2	1.393	0.002859218	Up	1.154	1.071	1.076	0.858	0.775	0.736
Q8C1A5	Thimet oligopeptidase	Thop1	1.339	0.002862273	Up	1.181	1.107	1.193	0.927	0.812	0.861
Q9Z0M5	Lysosomal acid lipase/cholesteryl ester hydrolase	Lipa	1.471	0.002930145	Up	1.291	1.235	1.312	0.784	0.869	0.956
Q8R2K4	TAF6-like RNA polymerase II p300/CBP-associated factor-associated factor 65 kDa	Taf6l	0.687	0.002952228	Down	0.936	0.948	0.808	1.37	1.304	1.244
P29452	Caspase-1	Casp1	1.449	0.002982141	Up	1.037	1.087	1.034	0.655	0.73	0.794
Q9DCZ4	MICOS complex subunit Mic26	Apoo	0.67	0.003052409	Down	0.822	0.917	0.829	1.348	1.15	1.336
Q9DAT5	Mitochondrial tRNA-specific 2-thiouridylase 1	Trmu	0.757	0.003084419	Down	0.94	0.924	0.86	1.12	1.249	1.228
P15119	Mast cell protease 2	Mcpt2	1.449	0.003105331	Up	1.322	1.291	1.411	0.984	0.833	0.961
Q9R1B9	Slit homolog 2 protein	Slit2	0.655	0.003205204	Down	0.905	0.755	0.828	1.164	1.336	1.299
P56382	ATP synthase subunit epsilon, mitochondrial	Atp5f1e	0.716	0.00325447	Down	0.904	0.877	0.882	1.356	1.134	1.227
Q9QZ49	UBX domain-containing protein 8	Ubxn8	0.66	0.003323111	Down	0.815	0.7	0.802	1.171	1.263	1.078
Q9CXV1	Succinate dehydrogenase [ubiquinone] cytochrome b small subunit,	Sdhd	0.704	0.00332498	Down	0.904	0.882	0.881	1.208	1.403	1.176
Q9D164	FXYD domain-containing ion transport	Fxyd6	1.407	0.003345912	Up	1.021	1.024	1.153	0.703	0.778	0.792
P23953	Carboxylesterase 1C	Ces1c	1.352	0.003380211	Up	1.193	1.167	1.091	0.851	0.91	0.791
Q61830	Macrophage mannose receptor 1	Mrc1	1.413	0.003402834	Up	1.142	1.132	1.251	0.795	0.909	0.79
P24452	Macrophage-capping protein	Capg	1.593	0.003429141	Up	1.276	1.337	1.386	0.759	0.959	0.792
Q60997	Deleted in malignant brain tumors 1 protein	Dmbt1	0.553	0.003523445	Down	0.933	0.943	0.796	1.755	1.37	1.709
Q99MZ7	Peroxisomal trans-2-enoyl-CoA reductase	Pecr	0.731	0.003643362	Down	0.849	0.919	0.952	1.195	1.188	1.337
B1AR13	CDGSH iron-sulfur domain-containing protein 3, mitochondrial	Cisd3	0.716	0.00367074	Down	0.834	0.868	0.883	1.212	1.305	1.092
Q640L3	Cell cycle progression protein 1	Ccpg1	0.75	0.003694466	Down	0.837	0.852	0.963	1.142	1.179	1.215
Q6P9S0	Protein MTSS 2	Mtss2	0.759	0.00371732	Down	0.927	0.875	0.884	1.225	1.231	1.084
Q9CQ91	NADH dehydrogenase [ubiquinone] 1 alpha subcomplex subunit 3	Ndufa3	0.756	0.003722188	Down	0.948	0.992	0.927	1.219	1.204	1.371

Q9WTX5	S-phase kinase-associated protein 1	Skp1	1.327	0.003914532	Up	1.176	1.143	1.161	0.943	0.802	0.878
P53690	Matrix metalloproteinase-14	Mmp14	0.719	0.003947423	Down	0.902	0.908	0.784	1.198	1.262	1.146
Q8R1S0	Ubiquinone biosynthesis monooxygenase COQ6, mitochondrial	Coq6	0.71	0.004020761	Down	0.798	0.894	0.93	1.147	1.284	1.263
E9Q4F7	Ankyrin repeat domain-containing protein 11	Ankrd11	0.687	0.004053216	Down	0.954	0.795	0.822	1.287	1.173	1.28
Q9CWW6	Peptidyl-prolyl cis-trans isomerase NIMA-interacting 4	Pin4	1.424	0.004070782	Up	1.193	1.317	1.175	0.902	0.782	0.904
P62960	Y-box-binding protein 1	Ybx1	1.312	0.004128437	Up	1.163	1.108	1.235	0.914	0.926	0.832
P70697	Uroporphyrinogen decarboxylase	Urod	1.32	0.004145497	Up	1.25	1.181	1.15	0.859	0.978	0.876
O55234	Proteasome subunit beta type-5	Psmb5	1.427	0.004150178	Up	1.341	1.259	1.408	0.843	0.984	0.982
Q9CXW4	60S ribosomal protein L11	Rpl11	1.321	0.004150264	Up	1.124	1.016	1.055	0.84	0.831	0.748
Q505D7	Optic atrophy 3 protein homolog	Opa3	0.769	0.004256243	Down	0.93	0.848	0.858	1.136	1.213	1.078
P21460	Cystatin-C	Cst3	0.726	0.004354442	Down	0.894	0.928	0.975	1.204	1.409	1.237
Q8VEA4	Mitochondrial intermembrane space import and assembly	Chchd4	0.647	0.004532467	Down	0.869	0.848	0.749	1.128	1.378	1.308
Q9D7S7	60S ribosomal protein L22-like 1	Rpl22l1	1.768	0.00466798	Up	1.152	1.187	1.281	0.583	0.81	0.655
Q7TQK1	Integrator complex subunit 7	Ints7	1.332	0.004693076	Up	1.117	1.164	1.075	0.91	0.83	0.779
Q64302	Transmembrane 4 L6 family member 1	Tm4sf1	0.764	0.004693127	Down	0.912	0.911	0.816	1.096	1.214	1.144
Q9D0L4	AarF domain-containing protein kinase 1	Adck1	0.689	0.004701133	Down	0.852	0.94	0.752	1.214	1.264	1.212
Q9R0B6	Laminin subunit gamma-3	Lamc3	1.484	0.004821366	Up	0.968	1.038	1.089	0.761	0.707	0.617
P70404	Isocitrate dehydrogenase [NAD] subunit gamma 1, mitochondrial	Idh3g	0.767	0.004852508	Down	0.863	0.861	0.896	1.193	1.039	1.184
P54731	FAS-associated factor 1	Faf1	1.302	0.004870499	Up	1.097	1.135	1.151	0.904	0.79	0.904
Q9ERP3	Tripartite motif-containing protein 54	Trim54	0.535	0.004931165	Down	0.627	0.838	0.767	1.215	1.555	1.403
P54869	Hydroxymethylglutaryl-CoA synthase,	Hmgcs2	0.628	0.004932829	Down	1.039	0.897	1.043	1.393	1.607	1.746
Q9CQR2	40S ribosomal protein S21	Rps21	1.415	0.005009755	Up	1.285	1.168	1.077	0.872	0.776	0.846
Q8CI04	Conserved oligomeric Golgi complex subunit 3	Cog3	1.32	0.005021561	Up	1.224	1.126	1.148	0.813	0.942	0.894
P62077	Mitochondrial import inner membrane translocase subunit Tim8	Timm8b	0.663	0.005040027	Down	0.77	0.787	0.861	1.272	1.065	1.309
Q7TNS2	MICOS complex subunit Mic10	Micos10	0.679	0.005100417	Down	0.874	0.85	0.788	1.339	1.09	1.27
P03888	NADH-ubiquinone oxidoreductase chain 1	Mtnd1	0.728	0.005153701	Down	0.965	0.857	0.802	1.202	1.243	1.16
P01921	H-2 class II histocompatibility antigen, A-D beta chain	H2-Ab1	0.721	0.005280728	Down	0.505	0.466	0.532	0.752	0.688	0.644
P36371	Antigen peptide transporter 2	Tap2	0.732	0.005498432	Down	0.664	0.623	0.577	0.894	0.868	0.783
P51141	Segment polarity protein dishevelled homolog	Dvl1	0.617	0.005521712	Down	0.815	0.861	0.68	1.209	1.197	1.411
Q9CR98	Protein FAM136A	Fam136a	0.705	0.005569744	Down	0.888	0.8	0.934	1.352	1.2	1.166
P18293	Atrial natriuretic peptide receptor 1	Npr1	0.764	0.005762154	Down	0.893	0.884	0.986	1.121	1.256	1.239
Q7TT37	Elongator complex protein 1	Elp1	1.301	0.005793794	Up	1.102	1.105	1.172	0.917	0.791	0.889
P17809	Solute carrier family 2, facilitated glucose transporter member 1	Slc2a1	1.335	0.005937007	Up	1.272	1.249	1.244	0.964	1.012	0.844
Q9D2U9	Histone H2B type 3-A	Hist3h2ba	0.754	0.006082359	Down	0.554	0.644	0.6	0.758	0.842	0.784
Q8BRC6	Cilia- and flagella-associated protein 91	Maats1	0.676	0.00615923	Down	0.829	0.773	0.691	1.242	1.113	1.039
Q921M7	CYFIP-related Rac1 interactor B	Cyrib	1.548	0.006186596	Up	1.236	1.305	1.252	0.88	0.882	0.688
Q61324	Aryl hydrocarbon receptor nuclear	Arnt2	0.736	0.006350464	Down	0.92	0.96	0.881	1.363	1.136	1.254
O70622	Reticulon-2	Rtn2	0.617	0.006411751	Down	0.743	0.933	0.722	1.408	1.281	1.2
P08101	Low affinity immunoglobulin gamma	Fcgr2	2.461	0.006437906	Up	1.629	1.404	1.455	0.791	0.436	0.597

Q80TY0	Formin-binding protein 1	Fnbp1	1.321	0.006667252	Up	1.111	1.096	1.152	0.857	0.767	0.918
Q9D385	ADP-ribosylation factor-like protein 2-binding	Arl2bp	1.34	0.007187353	Up	1.17	1.071	0.976	0.776	0.786	0.838
O88491	Histone-lysine N-methyltransferase, H3 lysine-36 specific	Nsd1	1.526	0.007219957	Up	1.167	1.217	1.19	0.755	0.907	0.68
Q60596	DNA repair protein XRCC1	Xrcc1	1.306	0.007554527	Up	1.118	1.087	1.032	0.908	0.78	0.791
Q9Z172	Small ubiquitin-related modifier 3	Sumo3	1.336	0.007616842	Up	1.108	1.178	1.093	0.887	0.755	0.888
P13439	Uridine 5'-monophosphate synthase	Umps	1.372	0.00790067	Up	1.143	1.087	1.083	0.709	0.834	0.871
Q91ZM2	SH2B adapter protein 1	Sh2b1	0.631	0.007943307	Down	0.818	0.834	0.694	1.206	1.101	1.413
Q61247	Alpha-2-antiplasmin	Serpinf2	1.768	0.008153852	Up	1.241	1.462	1.559	0.657	0.881	0.872
Q9EP73	Programmed cell death 1 ligand 1	Cd274	0.626	0.008258624	Down	0.53	0.718	0.569	1.007	0.993	0.903
Q7TNG8	Probable D-lactate dehydrogenase, mitochondrial	Ldhd	0.756	0.008359884	Down	0.835	0.972	0.97	1.205	1.174	1.293
Q9JIY5	Serine protease HTRA2, mitochondrial	Htra2	0.731	0.008495813	Down	0.904	0.76	0.844	1.239	1.112	1.082
P49717	DNA replication licensing factor MCM4	Mcm4	1.567	0.008646548	Up	0.927	1.159	1.124	0.767	0.657	0.624
P28656	Nucleosome assembly protein 1-like 1	Nap1l1	1.366	0.008689737	Up	1.272	1.096	1.06	0.891	0.806	0.812
Q6ZQ73	Cullin-associated NEDD8-dissociated	Cand2	0.731	0.008804237	Down	0.869	0.92	0.944	1.308	1.328	1.101
P56394	Cytochrome c oxidase copper chaperone	Cox17	0.679	0.009136309	Down	0.813	0.84	0.724	1.1	1.329	1.072
Q8BQU3	Succinate dehydrogenase assembly factor 3, mitochondrial	Sdhaf3	0.701	0.009272833	Down	0.771	0.742	0.888	1.151	1.035	1.239
P09541	Myosin light chain 4	Myl4	0.739	0.009329779	Down	0.961	1.058	0.873	1.273	1.248	1.391
Q9CPR1	RWD domain-containing protein 4	Rwdd4	1.309	0.00935139	Up	1.133	1.07	1.175	0.841	0.945	0.795
Q8K4M5	COMM domain-containing protein 1	Commd1	1.325	0.009373785	Up	1.204	1.065	1.171	0.828	0.949	0.819
Q08093	Calponin-2	Cnn2	1.393	0.00948556	Up	0.958	0.877	0.937	0.743	0.59	0.657
P19182	Interferon-related developmental regulator	Ifrd1	1.709	0.009564992	Up	1.344		1.513	0.938	0.765	0.804
Q9Z130	Heterogeneous nuclear ribonucleoprotein D-like	Hnrnpdl	1.307	0.009683157	Up	1.195	1.286	1.098	0.851	0.934	0.953
Q62422	Osteoclast-stimulating factor 1	Ostf1	1.307	0.009839741	Up	1.132	1.1	1.091	0.775	0.942	0.826
Q9DBM2	Peroxisomal bifunctional enzyme	Ehhadh	0.713	0.010204489	Down	0.955	0.832	0.892	1.337	1.315	1.103
Q9CPX6	Ubiquitin-like-conjugating enzyme	Atg3	1.347	0.010244311	Up	1.118	1.235	1.056	0.789	0.921	0.821
Q3TVI8	Pre-B-cell leukemia transcription factor-interacting protein 1	Pbxip1	0.748	0.010551136	Down	0.877	1.074	0.911	1.298	1.229	1.299
Q9CPX8	Cytochrome b-c1 complex subunit 10	Uqcr11	0.698	0.010741216	Down	0.904	0.863	0.946	1.124	1.45	1.314
P00397	Cytochrome c oxidase subunit 1	Mtco1	0.755	0.010880521	Down	1.025	0.903	0.911	1.183	1.373	1.203
Q80SW1	S-adenosylhomocysteine hydrolase-like protein 1	Ahcyl1	1.352	0.010908609	Up	1.23	1.111	1.253	0.809	0.981	0.868
P62849	40S ribosomal protein S24	Rps24	1.361	0.011598577	Up	0.955	0.928	0.923	0.724	0.595	0.743
P97500	Myelin transcription factor 1-like protein	Myt1l	1.316	0.011618208	Up	1.009	0.988	1.16	0.793	0.753	0.853
P19137	Laminin subunit alpha-1	Lama1	1.517	0.01181739	Up	1.197	1.163	1.068	0.681	0.684	0.895
P99024	Tubulin beta-5 chain	Tubb5	1.307	0.011824288	Up	1.162	1.05	1.072	0.751	0.887	0.874
Q99JR5	Tubulointerstitial nephritis antigen-like	Tinagl1	1.327	0.011837655	Up	1.226	1.212	1.259	0.848	1.049	0.889
Q91YN5	UDP-N-acetylhexosamine	Uap1	1.316	0.011891498	Up	1.132	1.149	1.208	0.993	0.836	0.822
Q8BUY5	Complex I assembly factor TIMMDC1,	Timmdc1	0.706	0.011962541	Down	0.899	0.723	0.835	1.247	1.174	1.058
P36993	Protein phosphatase 1B	Ppm1b	1.338	0.012311532	Up	1.273	1.063	1.171	0.812	0.939	0.871
Q6ZWY3	40S ribosomal protein S27-like	Rps27l	1.336	0.012334441	Up	1.006	0.962	1.001	0.753	0.818	0.652
Q80XA6	RalBP1-associated Eps domain-containing	Reps2	0.534	0.012432923	Down	0.66	0.626	0.622	1.381	0.886	1.309
Q60952	Centrosome-associated protein CEP250	Cep250	0.757	0.012452781	Down	0.979	0.845	0.948	1.27	1.28	1.11

E9PZM4	Chromodomain-helicase-DNA-binding protein 2	Chd2	0.712	0.012473169	Down	0.405	0.429	0.404	0.657	0.574	0.507
Q923X1	Adhesion G protein-coupled receptor L4	Adgrl4	0.764	0.012542336	Down	0.872	0.9	0.886	1.222	1.023	1.234
Q9CXZ1	NADH dehydrogenase [ubiquinone] iron-sulfur protein 4, mitochondrial	Ndufs4	0.757	0.012602201	Down	0.897	0.847	0.907	1.251	1.032	1.22
Q3UHB1	5'-nucleotidase domain-containing protein 3	Nt5dc3	0.721	0.0126883	Down	0.9	1.018	0.795	1.182	1.29	1.291
O55026	Ectonucleoside triphosphate	Entpd2	0.587	0.013230875	Down	0.782	0.693	0.647	1.206	0.978	1.433
Q60870	Receptor expression-enhancing protein 5	Reep5	0.76	0.0132388	Down	0.917	0.924	0.905	1.289	1.057	1.267
A2AR02	Peptidyl-prolyl cis-trans isomerase G	Ppig	1.434	0.013426124	Up	1.142	1.304	0.998	0.851	0.792	0.758
Q9CZB0	Succinate dehydrogenase cytochrome b560 subunit, mitochondrial	Sdhc	0.732	0.013685542	Down	0.874	0.918	0.857	1.278	1.043	1.296
P97311	DNA replication licensing factor MCM6	Mcm6	1.398	0.014037357	Up	1.223	1.126	1.118	0.862	0.91	0.708
A2AQ19	RNA polymerase-associated protein RTF1 homolog	Rtf1	1.301	0.014092774	Up	1.066	1.065	1.039	0.914	0.785	0.738
Q8BGR9	Ubiquitin-like domain-containing CTD phosphatase 1	Ublcp1	1.324	0.014510327	Up	1.228	1.249	1.11	1.01	0.856	0.844
Q3UN70	Myelin regulatory factor-like protein	Myrfl	0.625	0.015027011	Down	0.573	0.549	0.78	1.095	0.993	0.956
Q5U4D9	THO complex subunit 6 homolog	Thoc6	1.311	0.015201939	Up	1.049	1.158	0.985	0.844	0.853	0.737
Q61771	Kinesin-like protein KIF3B	Kif3b	1.365	0.015417122	Up	1.104	1.101	1.084	0.823	0.689	0.898
P06800	Receptor-type tyrosine-protein phosphatase C	Ptprc	1.866	0.015549905	Up	1.25	1.096	1.316	0.729	0.476	0.758
Q8CH40	Nucleoside diphosphate-linked moiety X motif 6	Nudt6	0.723	0.015742146	Down	0.845	0.88	0.794	1.21	1	1.273
P63280	SUMO-conjugating enzyme UBC9	Ube2i	1.327	0.015810941	Up	1.19	1.108	1.186	0.908	0.764	0.954
P27512	Tumor necrosis factor receptor superfamily member 5	Cd40	1.382	0.016249174	Up	1.234	1.123	1.329	1.009	0.829	0.83
P01901	H-2 class I histocompatibility antigen, K-B alpha chain	H2-K1	0.733	0.016696405	Down	0.69	0.905	0.776	1.086	1.047	1.102
P52912	Nucleolysin TIA-1	Tia1	1.833	0.017033489	Up	1.276	1.289	1.257	0.516	0.67	0.899
Q8R3Q0	Store-operated calcium entry-associated regulatory factor	Saraf	0.735	0.017231496	Down	0.88	0.829	0.903	1.206	1.028	1.32
Q8BGY7	Protein FAM210A	Fam210a	0.756	0.017346115	Down	0.777	0.886	0.8	1.211	0.993	1.054
O35343	Importin subunit alpha-3	Kpna4	1.323	0.017461741	Up	1.069	1.076	1.098	0.885	0.704	0.863
Q8BGF7	PAN2-PAN3 deadenylation complex catalytic subunit Pan2	Pan2	1.342	0.017707801	Up	1.049	1.217	1.132	0.943	0.759	0.83
Q5I012	Putative sodium-coupled neutral amino acid transporter 10	Slc38a10	0.694	0.017846752	Down	0.798	0.905	0.922	1.254	1.087	1.439
Q4G0F8	Ubinuclein-1	Ubn1	1.408	0.018313297	Up	1.034	1.117	0.836	0.7	0.717	0.705
O70493	Sorting nexin-12	Snx12	1.309	0.018549881	Up	1.028	1.179	1.255	0.825	0.939	0.88
Q8BIG7	Catechol O-methyltransferase domain-containing	Comtd1	0.723	0.018868468	Down	0.936	0.707	0.887	1.195	1.149	1.155
P54227	Stathmin	Stmn1	1.328	0.019083538	Up	1.047	1.069	1.1	0.918	0.709	0.794
Q9CWU6	Ubiquinol-cytochrome-c reductase complex assembly factor 1	Uqcc1	0.755	0.020054963	Down	0.817	0.863	0.863	1.142	0.978	1.25
O08692	Neutrophilic granule protein	Ngp	0.712	0.020067987	Down	0.876	0.882	0.895	1.43	1.053	1.243
Q9CQS4	Solute carrier family 25 member 46	Slc25a46	0.64	0.020130976	Down	0.984	0.652	0.848	1.228	1.323	1.333
Q08AU7	Myeloid-associated differentiation marker-like protein 2	Myadml2	1.359	0.020613821	Up	1.18	1.265	1.054	0.962	0.839	0.773
Q3TCJ1	BRISC complex subunit Abraxas 2	Abraxas2	1.39	0.02068791	Up	1.178	1.09	1.231	0.929	0.708	0.88
Q9CQN3	Mitochondrial import receptor subunit TOM6 homolog	Tomm6	0.764	0.021066876	Down	0.798	0.891	0.917	1.259	1.028	1.122

P15864	Histone H1.2	H1-2	0.553	0.021543761	Down	0.317	0.329	0.316	0.556	0.746	0.437
Q8BWU5	Probable tRNA N6-adenosine threonylcarbamoyltransferase	Osgep	1.356	0.021698068	Up	1.077	1.164	1.348	0.958	0.805	0.884
Q8C878	NEDD8-activating enzyme E1 catalytic	Uba3	1.355	0.021903261	Up	1.096	1.169	1.148	0.921	0.707	0.89
Q8R1M2	Histone H2A.J	H2aj	0.514	0.023123871	Down	0.205	0.134	0.166	0.39	0.346	0.246
A2A5Z6	E3 ubiquitin-protein ligase SMURF2	Smurf2	0.737	0.023704515	Down	0.843	1.027	0.898	1.102	1.301	1.354
Q6EDY6	F-actin-uncapping protein LRRC16A	Carmil1	0.425	0.023737056	Down	0.653	0.692	0.704	1.046	2.281	1.491
Q9D735	Telomerase RNA component interacting RNase	Trir	0.721	0.024869208	Down	0.582	0.761	0.676	0.995	0.969	0.837
P99028	Cytochrome b-c1 complex subunit 6,	Uqcrh	0.572	0.025199363	Down	0.663	0.686	0.771	1.413	0.9	1.391
P84228	Histone H3.2	H3c2	0.677	0.025352691	Down	0.25	0.228	0.207	0.384	0.35	0.278
P61290	Proteasome activator complex subunit 3	Psme3	1.325	0.025834796	Up	1.266	1.214	1.248	1.024	0.999	0.791
Q08288	Cell growth-regulating nucleolar protein	Lyar	0.765	0.027239429	Down	0.708	0.733	0.868	0.968	0.946	1.104
Q8C3S2	Transport and Golgi organization protein 6 homolog	Tango6	0.469	0.027602799	Down	0.51	0.524	0.531	1.478	0.719	1.14
P10922	Histone H1.0	H1-0	0.687	0.028890901	Down	0.307	0.258	0.282	0.465	0.432	0.336
Q9Z2Q6	Septin-5	Septin5	1.579	0.028993108	Up	1.458	1.242	1.108	0.993	0.734	0.685
Q9D8Y1	Transmembrane protein 126A	Tmem126a	0.769	0.030195172	Down	0.757	0.927	0.915	1.133	1.033	1.214
P47713	Cytosolic phospholipase A2	Pla2g4a	1.451	0.030464557	Up	0.959	0.881	1.189	0.609	0.711	0.768
P16254	Signal recognition particle 14 kDa protein	Srp14	1.321	0.030736101	Up	1.034	0.903	1.185	0.787	0.827	0.749
C0HKE9	Histone H2A type 1-P	Hist1h2ap	0.564	0.030841063	Down	0.202	0.211	0.252	0.471	0.426	0.283
Q8CIG8	Protein arginine N-methyltransferase 5	Prmt5	1.389	0.030870241	Up	1.193	1.126	1.212	0.885	0.69	0.967
Q6NXH8	Methyltransferase-like protein 25	Mettl25	0.431	0.031442734	Down	0.136	0.116	0.127	0.399	0.303	0.177
P60603	Reactive oxygen species modulator 1	Romo1	0.726	0.032335197	Down	0.834	0.96	0.738	1.018	1.256	1.215
Q8C6I2	Succinate dehydrogenase assembly factor 2, mitochondrial	Sdhaf2	0.757	0.032757563	Down	0.882	0.82	0.957	1.007	1.233	1.273
P22907	Porphobilinogen deaminase	Hmbs	1.322	0.03325205	Up	1.001	1.164	1.096	0.917	0.706	0.844
Q9JLH8	Tropomodulin-4	Tmod4	0.718	0.035361429	Down	0.673	0.827	0.968	1.164	1.082	1.189
P08043	Zinc finger protein 2	Zfp2	1.448	0.0353792	Up	0.757	1	0.931	0.726	0.554	0.576
P62806	Histone H4	H4c1	0.748	0.035420076	Down	0.262	0.273	0.282	0.398	0.391	0.303
P48725	Pericentrin	Pcnt	0.677	0.036303707	Down	0.82	0.653	0.874	0.978	1.156	1.333
Q9DCC7	Isochorismatase domain-containing protein 2B	Isoc2b	0.712	0.036605594	Down	0.882	0.728	0.854	1.04	1.372	1.05
Q9EQ28	DNA polymerase delta subunit 3	Pold3	0.729	0.0366233	Down	0.753	0.834	0.657	1.045	1.148	0.887
Q8C3Q5	Protein shisa-7	Shisa7	0.711	0.038180437	Down	0.761	0.922	0.98	1.264	1.068	1.416
P48453	Serine/threonine-protein phosphatase 2B catalytic subunit beta isoform	Ppp3cb	1.311	0.040909212	Up	1.024	1.23	1.344	0.989	0.881	0.874
P49935	Pro-cathepsin H	Ctsh	1.352	0.042191125	Up	1.178	1.103	1.216	0.808	1.039	0.74
Q9CR09	Ubiquitin-fold modifier-conjugating enzyme 1	Ufc1	1.36	0.042519451	Up	1.14	1.082	1.201	0.708	1.009	0.799
Q99KC7	Small cell adhesion glycoprotein	Smagp	0.732	0.046799938	Down	0.948	0.775	0.86	0.976	1.235	1.317
Q8C0L6	Peroxisomal N(1)-acetyl-spermine/spermidine	Paox	1.312	0.047217752	Up	1.058	1.363	1.061	0.921	0.921	0.811
P56213	FAD-linked sulfhydryl oxidase ALR	Gfer	0.733	0.047320949	Down	0.672	0.873	0.88	0.999	1.249	1.061
Q08376	Zinc finger and BTB domain-containing protein 14	Zbtb14	0.726	0.047512781	Down	0.83	0.797	0.808	0.957	1.365	1.03
P17665	Cytochrome c oxidase subunit 7C,	Cox7c	0.734	0.047820056	Down	0.872	0.864	0.886	1.344	0.956	1.274
P63073	Eukaryotic translation initiation factor 4E	Eif4e	1.303	0.048617734	Up	1.03	1.11	1.216	0.722	0.932	0.921

Q8BHJ5	F-box-like/WD repeat-containing protein TBL1XR1	Tbl1xr1	1.326	0.04932054	Up	1.131	1.182	1.148	0.854	0.722	1.035
Q5RJH6	Protein SMG7	Smg7	1.333	0.049432926	Up	1.182	1.027	1.171	0.972	0.701	0.862
